# Measures of empathy and compassion: A scoping review

**DOI:** 10.1371/journal.pone.0297099

**Published:** 2024-01-19

**Authors:** Cassandra Vieten, Caryn Kseniya Rubanovich, Lora Khatib, Meredith Sprengel, Chloé Tanega, Craig Polizzi, Pantea Vahidi, Anne Malaktaris, Gage Chu, Ariel J. Lang, Ming Tai-Seale, Lisa Eyler, Cinnamon Bloss

**Affiliations:** 1 Centers for Integrative Health, Department of Family Medicine, University of California, San Diego, San Diego, California, United States of America; 2 Clarke Center for Human Imagination, School of Physical Sciences, University of California, San Diego, San Diego, California, United States of America; 3 Department of Psychiatry, University of California, San Diego, San Diego, California, United States of America; 4 San Diego State University/University of California San Diego Joint Doctoral Program in Clinical Psychology, San Diego, San Diego, California, United States of America; 5 T. Denny Sanford Institute for Empathy and Compassion, University of California, San Diego, San Diego, California, United States of America; 6 T. Denny Sanford Center for Empathy and Technology, University of California, San Diego, San Diego, California, United States of America; 7 Human Factors, Netherlands Organisation for Applied Scientific Research (TNO), Soesterberg, The Netherlands; 8 U.S. Department of Veteran Affairs, VA Boston Healthcare System, Boston, Massachusetts, United States of America; 9 Department of Psychiatry, Boston University Chobanian & Avedisian School of Medicine, Boston, Massachusetts, United States of America; 10 Compassion Clinic, San Diego, California, United States of America; 11 VA San Diego Center of Excellence for Stress and Mental Health, San Diego, California, United States of America; 12 Herbert Wertheim School of Public Health and Human Longevity Science, University of California, San Diego, San Diego, California, United States of America; 13 Departments of Family Medicine and Medicine (Bioinformatics), School of Medicine, University of California, San Diego, San Diego, California, United States of America; 14 T. Denny Sanford Center for Empathy and Compassion Training in Medical Education, University of California, San Diego, San Diego, California, United States of America; Ankara University Faculty of Medicine: Ankara Universitesi Tip Fakultesi, TURKEY

## Abstract

Evidence to date indicates that compassion and empathy are health-enhancing qualities. Research points to interventions and practices involving compassion and empathy being beneficial, as well as being salient outcomes of contemplative practices such as mindfulness. Advancing the science of compassion and empathy requires that we select measures best suited to evaluating effectiveness of training and answering research questions. The objective of this scoping review was to 1) determine what instruments are currently available for measuring empathy and compassion, 2) assess how and to what extent they have been validated, and 3) provide an online tool to assist researchers and program evaluators in selecting appropriate measures for their settings and populations. A scoping review and broad evidence map were employed to systematically search and present an overview of the large and diverse body of literature pertaining to measuring compassion and empathy. A search string yielded 19,446 articles, and screening resulted in 559 measure development or validation articles reporting on 503 measures focusing on or containing subscales designed to measure empathy and/or compassion. For each measure, we identified the type of measure, construct being measured, in what context or population it was validated, response set, sample items, and how many different types of psychometrics had been assessed for that measure. We provide tables summarizing these data, as well as an open-source online interactive data visualization allowing viewers to search for measures of empathy and compassion, review their basic qualities, and access original citations containing more detail. Finally, we provide a rubric to help readers determine which measure(s) might best fit their context.

## Introduction

Historically, psychological assessment has overwhelmingly focused on measuring human struggles, difficulties, and pathologies. However, converging evidence indicates that positive emotions and prosocial qualities are just as important for improving overall well-being as stress, depression, and anxiety are to detracting from health and well-being [1]. Across fields—from medicine, mental health care, and education to economics, business and organizational development—there is a growing emphasis on investigating prosocial constructs such as compassion and empathy [2].

Compassion, or the heartfelt wish to reduce the suffering of self and others, promotes social connection and is an important predictor of overall quality of life [2] and well-being [3]. Empathy, or understanding and vicariously sharing other people’s positive emotions, is related to prosocial behaviors (e.g., helping, giving, emotional support), positive affect, quality of life, closeness, trust, and relationship satisfaction [4]. Compassion and empathy improve parenting [5], classroom environments [6], and teacher well-being [7]. Compassionate love toward self and others is associated with disease outcomes as well, such as increased long-term survival rates in patients with HIV [8]. Self-compassion refers to being gentle, supportive, and understanding toward ourselves in instances of perceived failure, inadequacy, or personal suffering [9]. Research indicates that self-compassion appears to reduce anxiety, depression, and rumination [10], and increase psychological well-being and connections with others [11, 12]. Both compassion and self-compassion appear to protect against stress [13] and anxiety [10].

In healthcare professionals, empathy is associated with patient satisfaction, diagnostic accuracy, adherence to treatment recommendations, clinical outcomes, clinical competence, and physician retention [14–16]. Importantly, it is also linked to reduced burnout, medical errors, and malpractice claims [17]. However, evidence indicates that empathy declines during medical training and residency [18–20]. This may present an opportunity to improve many aspects of healthcare by identifying ways to maintain or enhance empathy during medical training. It is also important to note that while empathy is beneficial for patients, the effects on healthcare professionals are more complicated. A distinction can be drawn between *positive* empathy and/or compassion versus *over-empathizing*, which can lead to what has been termed “compassion fatigue” and/or burnout.

Disentangling these relationships through scientific investigation requires selecting measures and instruments capable of capturing these nuances. In addition, growing evidence that empathy and compassion can be improved through training [21, 22] relies on selection or development of measures that can assess the effectiveness of such training. While empathy and compassion training for healthcare professionals has shown positive outcomes, it still requires improvement. For example, in a recent systematic review, only 9 of 23 empathy education studies in undergraduate nursing samples demonstrated practical improvements in empathy [23]. Another systematic review of 103 compassion interventions in the healthcare context [24] identified a number of limitations such as focusing on only a single domain of compassion; inadequately defining compassion; assessing the constructs exclusively by self-report; and not evaluating retention, sustainability, and translation to clinical practice over time: all related to how compassion and empathy are conceptualized and measured. The researchers recommend that such interventions should “be grounded in an empirically-based definition of compassion; use a competency-based approach; employ multimodal teaching methods that address the requisite attitudes, skills, behaviors, and knowledge within the multiple domains of compassion; evaluate learning over time; and incorporate patient, preceptor, and peer evaluations” (p. 1057). Improving conceptualization and measurement of compassion and empathy are crucial to advancing effective training.

### Conceptualizing compassion and empathy

Compassion and empathy are complex constructs, and therefore challenging to operationalize and measure. Definitions of compassion and empathy vary, and while they are often used interchangeably, they are distinct constructs [25]. Like many other constructs, both compassion and empathy can be conceptualized at state and/or trait levels: people can have context-dependent experiences of empathy or compassion (i.e., state), or can have a general tendency to be empathic or compassionate (i.e., trait). The constructs of empathy and compassion each have multiple dimensions: affective, cognitive, behavioral, intentional, motivational, spiritual, moral and others. In addition to their multidimensionality, compassion and empathy are crowded by multiple adjacent constructs with which they overlap to varying degrees, such as kindness, caring, concern, sensitivity, respect, and a host of behaviors such as listening, accurately responding, patience, and so on.

Strauss et al. [26] conducted a systematic review of measures of compassion, and by combining the definitions of compassion among the few existing instruments at the time, proposed five elements of compassion: recognizing suffering, understanding the universality of human suffering, feeling for the person suffering, tolerating uncomfortable feelings, and motivation to act/acting to alleviate suffering. Gilbert [27] proposed that compassion consists of six attributes: sensitivity, sympathy, empathy, motivation/caring, distress tolerance, and non-judgement.

Likewise, empathy has been conceptualized as having at least four elements (as measured by the Interpersonal Reactivity Index [28] for example): perspective-taking (i.e., taking the point of view of others), fantasy (i.e., imagining or transposing oneself into the feelings and actions of others), empathic concern (i.e., accessing other-oriented feelings of sympathy or concern) and personal distress (i.e., or unease in intense interpersonal interactions). Early work by Wiseman [29] used a concept analysis approach identifying four key domains of empathy: seeing the world the way others see it, understanding their feelings, being non-judgmental, and communicating or expressing that understanding. Other conceptualizations of empathy [30] include subdomains of affective reactivity (i.e., being emotionally affected by others), affective ability (i.e., others tell me I’m good at understanding them), affective drive (i.e., I try to consider the other person’s feelings), cognitive drive (i.e., trying to understand or imagine how someone else feels), cognitive ability (i.e., I’m good at putting myself in another person’s shoes), and social perspective taking. De Waal and Preston [31] propose a “Russian doll” model of empathy, in which evolutionary advances in empathy layer one on top of the next, resulting in their definition of empathy as “emotional and mental sensitivity to another’s state, from being affected by and sharing in this state to assessing the reasons for it and adopting the other’s point of view” (p. 499).

Compassion is conceptualized as generally positive, and “more is better” in terms of health and well-being. Empathy on the other hand can lead to positive outcomes such as empathic concern, compassion, and prosocial motivations and behaviors, whereas unregulated empathic distress can be aversive, decrease helping behaviors, and lead to burnout [32]. Compassion and empathy also appear to differ in underlying brain structure [33] as well as brain function [34]. Terms such as “compassion fatigue” are more accurately characterized as empathy fatigue, and some evidence indicates that compassion can actually counteract negative aspects of empathy [35].

When assessing compassion and empathy, it is often important to measure their opposites, or constructs that present barriers to experiencing and expressing compassion or empathy. Personal distress, for example, can be confused for empathy but in fact is a “self-focused, aversive affective reaction” to encountering another person’s suffering, accompanied by the desire to “alleviate one’s own, but not the other’s distress” [36, p.72]. Personal distress is viewed as a barrier to true compassion, and experienced chronically, is associated with burnout (i.e. exhaustion, cynicism, and inefficacy due to feeling frenetic/overloaded, underchallenged/indifferent, or worn-out/neglected [37]).

Other constructs that have been measured as barriers to compassion include lack of empathy or empathy impairment, apathy, coldness, judgmental attitudes toward specific populations or conditions, and fear of compassion. In sum, compassion and empathy are not so much singular constructs as multi-faceted collections of cognitions, affects, motivations and behaviors. When researchers or program evaluators consider the best ways to assess empathy and compassion, they must often attend to measuring these constructs as well.

Past systematic reviews focused on measurement of empathy and compassion sought to (1) review definitions [26, 38]; (2) evaluate measurement methods [39]; (3) assess psychometric properties [40]; (4) provide quality ratings [26, 41, 42]; and/or (5) recommend gold standard measures [26, 43]. To our knowledge, this review is the first scoping review focused on capturing the wide array of instruments measuring empathy, compassion, and adjacent constructs.

We conducted a scoping review and broad evidence map (as opposed to a systematic review or meta-analysis) for several reasons. Whereas systematic reviews attempt to collate empirical evidence from a relatively smaller number of studies pertaining to a focused research question, scoping reviews are designed to employ a systematic search and article identification method to answer broader questions about a field of study. As such, this scoping review provides a large and diverse map of the available measures across this family of constructs and measurement methodology, with the primary goal of aiding researchers and program evaluators in selecting measures appropriate for their setting.

Another unique feature of this scoping review is a data visualization that we have developed to help readers navigate the findings. This interactive tool is called the Compassion and Empathy Measures Interactive Data Visualization (CEM-IDV) (https://imagination.ucsd.edu/compassionmeasures/).

The aims of this scoping review were achieved, including 1) identifying existing measures of empathy and compassion, 2) providing an overview of the evidence for validity of these measures, and 3) providing an online tool to assist researchers and program evaluators in searching for and selecting the most appropriate instruments to evaluate empathy, compassion, and/or adjacent constructs, based on their specific context, setting, or population.

## Methods

The objective of this project was to capture all peer-reviewed published research articles that were focused on developing, or assessing the psychometric properties of, instruments measuring compassion and empathy and overlapping constructs, such as self-compassion, theory of mind, perspective taking, vicarious pain, caring, the doctor-patient relationship, emotional cues, sympathy, tenderness and emotional intelligence. We included only articles that were specifically focused on measure development or validation, and therefore did not include articles that may have developed idiosyncratic ways of assessing compassion or empathy in service to conducting experiments. We included self-report assessments, observational ratings or behavioral coding schemes, and tasks. This review was conducted according to the PRISMA statement for scoping reviews [44]. The population, concept, and context (PCC) for this scoping review were 1) population: adults and children, 2) concepts: compassion and empathy, and 3) context: measures/questionnaires for English-speaking populations (behavioral measures and tasks in all languages).

### Eligibility criteria

Articles were included if they focused on development or psychometric validation/evaluation of whole or partial scales, tasks, or activities designed to measure empathy, compassion, or synonymous or adjacent constructs. Conference proceedings and abstracts as well as grey-literature were excluded from this review, as were articles in languages other than English or reporting on self-report scales that were in languages other than English. Behavioral tasks or observational measures that were conducted in languages other than English, but were reported in English and could be utilized in an English-speaking context, were included. Papers were excluded if they were in a language other than English, did not include human participants, or did not focus on reporting on development or psychometric validation of measures of compassion, empathy, or adjacent constructs.

### Information sources

To identify the peer-reviewed literature reporting on the psychometric properties of measures of empathy and compassion, the following databases were searched: PubMed, Embase, PsychInfo, CINAHL, and Sociological Abstracts. See Table 1 to review the search terms and strategy applied for each database. All databases were searched in October 2020 and again in May 2023 by a reference librarian trained in systematic and scoping reviews at the University of California, San Diego library.

**Table 1:** Databases and search strings.

Database	*Search Terms*			Number of Articles
	Empathy	Testing	Measurements	Total
**PubMed:**	("Empathy"[Mesh]) OR ("Empath*"[tiab]) OR ("compassion*"[tiab]) OR ("self-compassion"[tiab]) OR ("loving-kindness"[tiab]) OR ("sympathy"[tiab]) OR ("bedside manner"[tiab]) OR ("metta"[tiab]) OR ("karuna"[tiab]) OR ("ubuntu"[tiab]) OR ("emotional cue*"[tiab])	(“Psychometrics”[Mesh]) OR (“reproducibility of results”[Mesh]) OR (“Validation studies as topic”[Mesh]) OR (“bias”[Mesh]) OR (“observer variation”[Mesh]) OR ("Selection Bias"[Mesh]) OR (“diagnostic errors”[Mesh]) OR (“dimensional measurement accuracy”[Mesh]) OR (“predictive value of tests”[Mesh]) OR (“discriminant analysis”[Mesh]) OR ("psychometric*"[tiab]) OR ("reliabil*"[tiab]) OR ("valid*"[tiab]) OR ("reproducib*"[tiab]) OR ("bias"[tiab]) OR ("Factor Analysis, Statistical"[Mesh]) OR ("Biomarkers"[Mesh:NoExp]) OR ("biomarker"[tiab]) OR ("Magnetic Resonance Imaging"[Mesh]) OR ("electroencephalography"[MeSH Terms]) OR ("fMRI"[tiab]) OR ("electroencephalography"[tiab]) OR ("EEG"[tiab]) OR ("Functional Neuroimaging"[Mesh]) OR ("Autonomic Nervous System"[Mesh]) OR ("Parasympathetic Nervous System"[Mesh]) OR ("Sympathetic Nervous System"[Mesh]) OR ("Hormones"[Mesh]) OR ("Neurotransmitter Agents"[Mesh]) OR ("task"[tiab]) OR ("implicit"[tiab]) OR ("functional magnetic resonance imaging"[tiab]) OR ("respiratory sinus arrhythmia"[tiab]) OR ("rsa"[tiab]) OR ("vagal tone"[tiab]) OR ("heart rate variability"[tiab]) OR ("hrv"[tiab]) OR ("autonomic nervous system"[tiab]) OR ("parasympathetic nervous system"[tiab]) OR ("sympathetic nervous system"[tiab]) OR ("hormones"[tiab]) OR ("immune*"[tiab]) OR ("neurophysiolog*"[tiab]) OR ("neurobiolog*"[tiab])	("scale"[tiab]) OR ("Surveys and Questionnaires"[Mesh]) OR ("instrument"[tiab]) OR ("scale"[tiab]) OR ("subscale*"[tiab]) OR ("questionnaire*"[tiab]) OR ("modeling"[tiab]) OR (" Models, Psychological"[Mesh]) OR ("Interviews as Topic"[Mesh]) OR ("interview*"[tiab]) OR ("investigation*"[tiab]) OR ("blood "[Subheading]) OR ("analys*"[tiab]) OR ("analyz*"[tiab]) OR ("paradigm*"[tiab]) OR ("intervention*"[tiab]) OR ("measure*"[tiab]) OR ("Brain Mapping"[MeSH]) OR ("Neuroimaging"[Mesh])	6,829
**Embase:**	(empathy/exp) OR (compassion/exp) OR (kindness/exp) OR (sympathy/exp) OR (empath*:ti,ab) OR (compassion*:ti,ab) OR (self-compassion:ti,ab) OR (loving-kindness:ti,ab) OR (sympathy:ti,ab) OR (bedside manner:ti,ab) OR (metta:ti,ab) OR (karuna:ti,ab) OR (ubuntu:ti,ab) OR (emotional cue*:ti,ab)	(psychometry/exp) OR (reproducibility/exp) OR (validationstudy/exp) OR (statisticalbias/exp) OR (selectionbias/exp) OR (observervariation/exp) OR (diagnosticerror/exp) OR (dimensionalmeasurementaccuracy/exp) OR (predictivevalue/exp) OR (discriminantanalysis/exp) OR (psychometric*:ti,ab) OR (reliabil*:ti,ab) OR (valid*:ti,ab) OR (reproducib*:ti,ab) OR (bias:ti,ab) OR (factoranalysis/exp) OR (biologicalmarker/exp) OR (biomarker:ti,ab) OR (nuclearmagneticresonanceimaging/exp) OR (electroencephalography/exp) OR (fMRI:ti,ab) OR (electroencephalography:ti,ab) OR (EEG:ti,ab) OR (functionalneuroimaging/exp) OR (autonomicnervoussystem/exp) OR (cholinergicsystem/exp) OR (adrenergicsystem/exp) OR (hormonesandhormoneanalogs/exp) OR (agentsinteractingwithtransmitter,hormoneordrugreceptors/exp) OR (task:ti,ab) OR (implicit:ti,ab) OR (functionalmagneticresonanceimaging:ti,ab) OR (respiratorysinusarrhythmia:ti,ab) OR (rsa:ti,ab) OR (vagustone/exp) OR (vagaltone:ti,ab) OR (heartratevariability:ti,ab) OR (hrv:ti,ab) OR (parasympatheticnervoussystem:ti,ab) OR (sympatheticnervoussystem:ti,ab) OR (hormones:ti,ab) OR (immun*:ti,ab) OR (neurophysiolog*:ti,ab) OR (neurobiolog*:ti,ab)	(scale:ti,ab) OR (questionnaire/exp) OR (survey/exp) OR (instrument:ti,ab) OR (scale:ti,ab) OR (subscale*:ti,ab) OR (questionnaire*:ti,ab) OR (modeling:ti,ab) OR (psychologicalmodel/exp) OR (interview/exp) OR (interview*:ti,ab) OR (investigation*:ti,ab) OR (analys*:ti,ab) OR (analyz*:ti,ab) OR (paradigm*:ti,ab) OR (intervention*:ti,ab) OR (measure*:ti,ab) OR (brainmapping/exp) OR (neuroimaging/exp) OR (functionalneuroimaging/exp)	9,315
**CINAHL:**	(MH "Empathy") OR (MH "Compassion") OR (compassion*) OR (self-compassion) OR (loving-kindness) OR (sympathy) OR (“bedside manner”) OR (metta) OR (karuna) OR (ubuntu) OR (“emotional cue*”)	(MH "Measurement Issues and Assessments+") OR (MH "Reproducibility of Results") OR (MH "Validation Studies") OR (MH "Bias Research+") OR (MH "Diagnostic Errors+") OR (MH "Predictive Value of Tests") OR (MH "Discriminant Analysis") OR ("psychometric*") OR ("reliabil*") OR ("valid*") OR ("reproducib*") OR ("bias ") OR (MH "Factor Analysis") OR (MH "Biological Markers+") OR ("biomarker") OR (MH "Magnetic Resonance Imaging+") OR (MH "Electroencephalography") OR ("fMRI") OR ("electroencephalography") OR ("EEG") OR (MH "Neuroradiography+") OR (MH "Autonomic Nervous System+") OR (MH "Hormones+") OR (MH "Neurotransmitter Agents+") OR ("task") OR ("implicit ") OR ("functional magnetic resonance imaging") OR ("respiratory sinus arrhythmia ") OR ("rsa") OR ("vagal tone") OR ("heart rate variability") OR ("hrv ") OR ("autonomic nervous system") OR ("parasympathetic nervous system ") OR ("sympathetic nervous system ") OR ("hormones ") OR ("immune*") OR ("neurophysiolog*") OR ("neurobiolog*")	(MH "Instrument by Type+") OR (MH "Surveys+") OR (MH "Surveys+") OR ("questionnaire") OR ("instrument") OR ("scale") OR ("subscale") OR (MH "Models, Psychological+") OR (modeling:ti,ab) OR (MH "Interviews+") OR ("interview*") OR ("investigation*") OR ("analys*") OR ("analyz*") OR ("paradigm*") OR (MH "Paradigms") OR ("intervention*") OR ("measure*") OR (MH "Brain Mapping")	3,595; limited to academic journals = 3,257
**PsychINFO:**	MAINSUBJECT.EXACT(Empathy) OR MAINSUBJECT.EXACT.EXPLODE(Sympathy) OR AB(empathy) OR TI(empathy) OR AB(compassion*) OR TI(compassion*) OR AB(self-compassion) OR TI(self-compassion) OR AB(loving-kindness) OR TI(loving-kindness) OR AB(sympathy) OR TI(sympathy) OR AB(“bedside manner”) OR TI(“bedside manner”) OR AB(metta) OR TI(metta) OR AB(karuna) OR TI(karuna) OR AB(ubuntu) OR TI(ubuntu) OR AB(“emotioonal cue*”) OR TI(“emotional cue*”)	MAINSUBJECT.EXACT.EXPLODE(Psychometrics) OR MAINSUBJECT.EXACT(Statistical Validity) OR MAINSUBJECT.EXACT(Experimenter Bias) OR MAINSUBJECT.EXACT.EXPLODE(Test Bias) OR MAINSUBJECT.EXACT(Predictive Validity) OR AB(measurement instruments) OR TI(measurement instruments) AB(psychometric*) OR TI(psychometric*) OR AB(reliabil*) OR TI(reliabil*) OR AB(valid*) OR TI(valid*) OR AB(reproducib*) OR TI(reproducib*) OR AB(bias) OR TI(bias) OR MAINSUBJECT.EXACT.EXPLODE(Factor Analysis) OR MAINSUBJECT.EXACT.EXPLODE(Biological Markers) OR AB(biomarker) OR TI(biomarker) OR MAINSUBJECT.EXACT.EXPLODE(Magnetic Resonance Imaging) OR MAINSUBJECT.EXACT.EXPLODE(Functional Magnetic Resonance Imaging) OR MAINSUBJECT.EXACT.EXPLODE(Electroencephalography) OR AB(electroencephalography) OR TI(electroencephalography) OR AB(EEG) OR TI(EEG) OR MAINSUBJECT.EXACT.EXPLODE(Autonomic Nervous System) OR MAINSUBJECT.EXACT.EXPLODE(Hormones) OR MAINSUBJECT.EXACT.EXPLODE(Neurotransmitters) OR AB(task) OR TI(task) OR AB(implicit) OR TI(implicit) OR AB(functional magnetic resonance imaging) OR TI(functional magnetic resonance imaging) OR AB(fMRI) OR TI(fMRI) OR AB(respiratory sinus arrhythmia) OR TI(respiratory sinus arrhythmia) OR AB(rsa) OR TI(rsa) OR AB(vagal tone) OR TI(vagal tone) OR AB(heart rate variability) OR TI(heart rate variability) OR AB(hrv) OR TI(hrv) OR AB(autonomic nervous system) OR TI(autonomic nervous system) OR AB(parasympathetic nervous system) OR TI(parasympathetic nervous system) OR AB(sympathetic nervous system) OR TI(sympathetic nervous system) OR AB(hormones) OR TI(hormones) OR AB(immune*) OR TI(immune*) OR AB(neurophysiolog*) OR TI(neurophysiolog*) OR AB(neurobiolog*) OR TI(neurobiolog*) OR AB(neural plasticity) OR TI(neural plasticity)	MAINSUBJECT.EXACT.EXPLODE(Test Types) OR AB(questionnaire*) OR TI(questionnaire*) OR AB(instrument) OR TI(instrument) OR AB(scale) OR TI(scale) OR AB(subscale*) OR TI(subscale*) OR MAINSUBJECT.EXACT.EXPLODE(Mental Models) OR AB(modeling) OR TI(modeling) OR AB(interview*) OR TI(interview*) OR AB(investigation*) OR TI(investigation*) OR AB(analys*) OR TI(analys*) OR AB(analyz*) OR TI(analyz*) OR AB(paradigm*) OR TI(paradigm*) OR AB(intervention*) OR TI(intervention*) OR AB(brain regions) OR TI(brain regions) OR MAINSUBJECT.EXACT(Neuroanatomy) OR MAINSUBJECT.EXACT(Stereotaxic Atlas)	6,199; exclude dissertations and books = 5,056
**Soc Abstracts:**	MAINSUBJECT.EXACT.EXPLODE(Empathy) OR MAINSUBJECT.EXACT(Compassion) OR (AB(empathy) OR TI(empathy)) OR (compassion) OR (self-compassion) OR (loving-kindness) OR (AB(sympathy) OR TI(sympathy)) OR (bedside manner) OR (metta) OR (karuna) OR (ubuntu) OR (emotional cue)	MAINSUBJECT.EXACT(Psychometric Analysis) OR SUBJECT(Psychometric properties) OR MAINSUBJECT.EXACT.EXPLODE(Validity) OR MAINSUBJECT.EXACT.EXPLODE(Statistical Bias) OR MAINSUBJECT.EXACT(Test Bias) OR SUBJECT(Bias) OR (AB(predictive validity) OR TI(predictive validity)) OR (AB(psychometric*) OR TI(psychometric*)) OR (AB(reliabil*) OR TI(reliabil*)) OR (AB(valid*) OR TI(valid*)) OR (reproducib*) OR (bias) OR MAINSUBJECT.EXACT.EXPLODE(Factor Analysis) OR MAINSUBJECT.EXACT.EXPLODE(Biological Factors) OR (biomarker) OR (magnetic resonance imaging) OR (functional magnetic resonance imaging) OR SUBJECT(Electroencephalography) OR (electroencephalography) OR (EEG) OR SUBJECT(Nervous system) OR SUBJECT(Autonomic nervous system) OR SUBJECT(Parasympathetic nervous system) OR (nervous system) OR MAINSUBJECT.EXACT.EXPLODE(Hormones) OR SUBJECT(Neurotransmitters) OR (neurotransmitters) OR (task) OR (fmri) OR (AB(respiratory sinus arrhythmia OR rsa) OR TI(respiratory sinus arrhythmia OR rsa)) OR (vagal tone) OR (AB (heart rate variability OR hrv) OR TI (heart rate variability OR hrv)) OR (hormones) OR (immune*) OR (neurophysiolog*) OR (neurobiolog*) OR SUBJECT(Neurophysiology/Neurophysiological) OR (neural plasticity)	MAINSUBJECT.EXACT.EXPLODE(Measures (Instruments)) OR MAINSUBJECT.EXACT(Surveys) OR MAINSUBJECT.EXACT(Interviews) OR MAINSUBJECT.EXACT.EXPLODE(QuesTIonnaires) OR (AB(questionnaire*) OR TI(questionnaire*)) OR (AB(survey*) OR TI(survey*)) OR (AB(subscale*) OR TI(subscale*)) OR (AB(scale*) OR TI(scale*)) OR (AB(instrument*) OR TI(instrument*)) OR (AB(model*) OR TI(model*)) OR (AB(interview*) OR TI(interview*)) OR (AB(invesTIgaTIon) OR TI(invesTIgaTIon)) OR (AB(analys*) OR TI(analys*)) OR (AB(analyz*) OR TI(analyz*)) OR (AB(paradigm*) OR TI(paradigm*)) OR MAINSUBJECT.EXACT.EXPLODE(Paradigms) OR (brain mapping) OR MAINSUBJECT.EXACT.EXPLODE(Neurology) OR SUBJECT(Brain)	6,314; excluding dissertations, magazines, & books = 4,662

### Screening

Abstracts of the articles identified through the search were uploaded to Covidence [45, 46]. Covidence is a web-based collaboration software platform that streamlines the production of systematic and other literature reviews. Each article was screened by two reviewers and any conflicts reviewed in team meetings until the team reached 90% agreement. Thereafter, one screener included or excluded each abstract.

### Full text screening

After articles were screened in, full text for all articles tagged as “Measure Development/Validation” were uploaded to the system. The project coordinator (MS) reviewed all articles that were included to ensure that they were tagged appropriately and that all articles reporting on development or validation of measures or assessments of psychometric properties were included in this review.

### Reviewing

Each article was reviewed for its general characteristics and psychometric evaluation/validation data reported. General data extracted from each article included: the article title, full citation, abstract, type of study, the name of the scale/assessment/measure, the author’s definition of the construct(s) being measured (if stated), the specific purpose of the scale (context and population, such as “a scale for measuring nurses’ compassion in patient interactions”), whether the measure was conceptualized as assessing state or trait (or neither or both); whether the scale was self-report, peer-report, or expert observer/coder; the validation population, number, gender proportion, and location; and any reviewer notes.

See Table 2 for the psychometric data extracted from each article. In this scoping review we did *not* evaluate or record/analyze the *results* of the psychometric evaluations or validations. We only recorded whether or not they had been completed. Because some members of the team did not have enough experience/training to properly identify psychometric evaluations or assessments, data extraction was completed using two data extraction forms (i.e., one for general data and one for psychometric data) constructed in Survey Planet [47]. A group of four experienced coders completed both the general and psychometric data extraction forms, and a group of six less experienced coders completed only the general data extraction form with an experienced coder completing the psychometric data extraction form.

**Table 2:** Definitions of psychometric properties.

Psychometric Property	Definition
**Reliability**	
Internal Consistency	The strength of relationships between the items in the measure that tap the construct
Test Re-test Reliability	Test the resulting items again to see if/how they measure the same thing over time.
Inter-rater Reliability	The correlation or relationship between two different raters responses
**Validity**	
Content Validity	Assesses whether a test is representative of all aspects of the construct.
Construct Validity	The extent to which the measured variable appears to be an adequate measure of the conceptual variable.
Convergent Validity	The extent to which a measured variable is found to be related to other measured variables designed to measure the same or similar conceptual variables.
Divergent/Discriminant Validity	The extent to which a measured variable is found to be unrelated to other measured variables designed to measure different conceptual characteristics, or the extent to which a measured variable discriminates between groups in a sample.
Predictive Validity	The extent to which a self-report measure correlates with (predicts) a future behavior.
**Other Psychometrics**	
Factor Analysis/Principal Component Analysis	The extent which a scale falls into sub-categories
Confirmatory Factor Analysis	A set of factors (facets or subscales) has already been identified for the measure. The researcher investigates whether these factors “hold up” in another new sample, or whether a different factor structure is a better fit.
Structural Equation Modeling	Combines factor analysis and multiple regression analysis to analyze the structural relationship between measured variables and latent constructs
Control/Correlation with Social Desirability	The extent to which the measure correlates with a scale of social desirability.
Other Biased Responding or Lie Scale	Whether the measure includes, or the degree of correlation has been assessed with, items or scales intended to detect biased, dishonest or random responding.

Once the data were extracted, they were reviewed by the research coordinator or principal investigator and combined into a spreadsheet. After combining, the answers were reviewed by a team of four additional reviewers to ensure that the information extracted was correct. These four reviewers received additional training on how to confirm that the appropriate information was extracted from the article as well as how to clean the information in a systematic way.

## Results

### Systematic literature search

**Fig 1:**
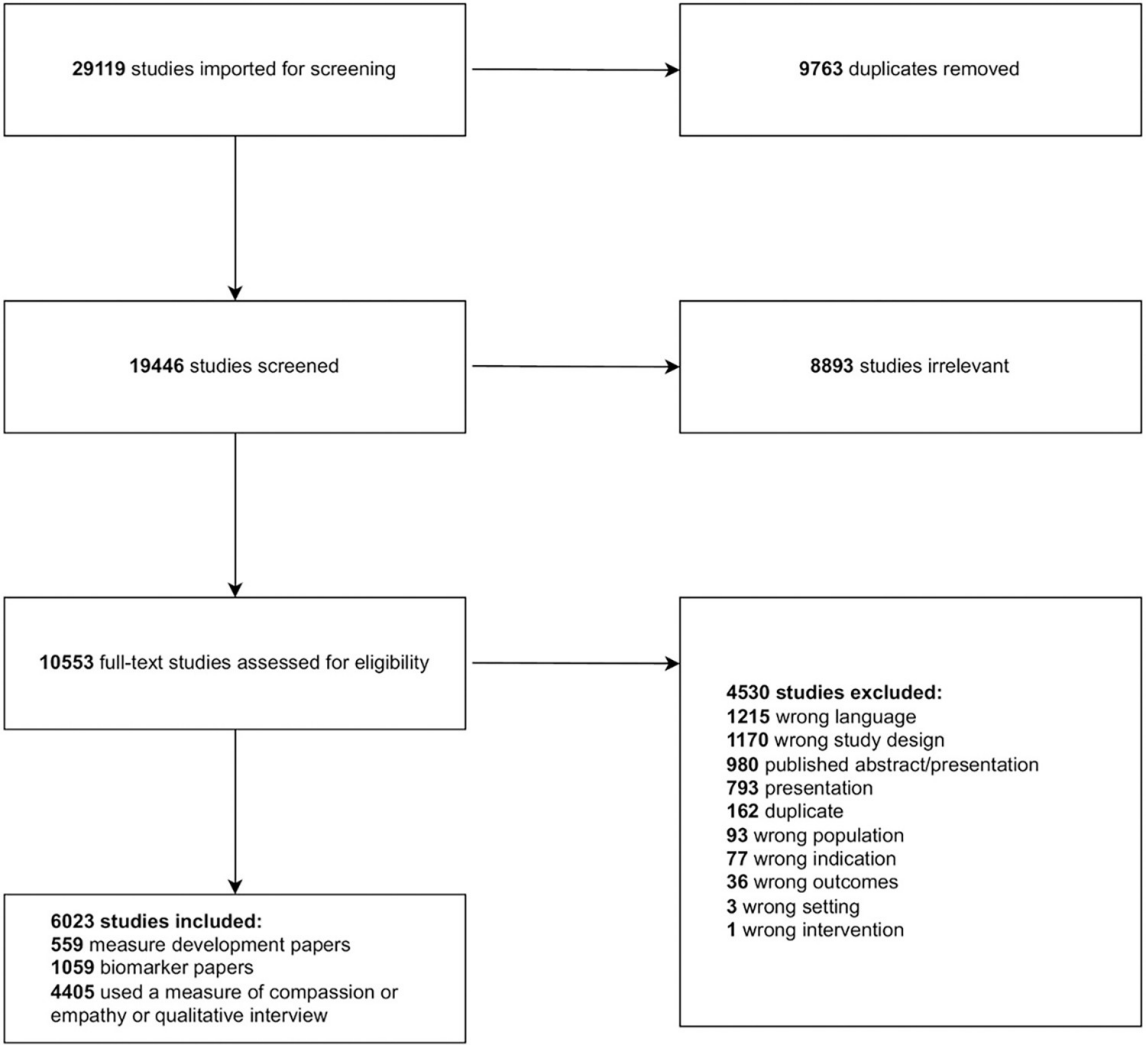
Article screening flow diagram.

A total of 29,119 articles were identified and 9,673 duplicates were removed, resulting in 19,446 titles/abstracts screened for eligibility (Fig 1). A total of 10,553 full-text articles were assessed for inclusion based on the criteria previously described. A total of 6,023 articles were included in the final sample. Of these articles, 559 reported on the development or validation of a measure of empathy and/or compassion, 1,059 identified biomarkers of empathy and/or compassion, and 3,936 used a measure or qualitative interview of empathy or compassion in the respective study. This scoping review reports on the 559 measure development/validation articles.

### Measure development and validation studies

An overview of the 503 measures of empathy or compassion that were developed, validated, or psychometrically evaluated in the 559 articles can be found in the S1 Table. The majority of the studies (*n* = 181) used a student population for development and/or validation. Student populations included undergraduate students, nursing students, and medical students. A total of 136 studies used samples of general, healthy adults (18 and older). Eighty-three (83) studies developed and/or validated a measure using health care workers, mostly comprising physicians and nursing staff. A total of 66 studies reported on a combined sample of populations such as clinicians and patients. There were 63 studies that used a patient population (e.g., cancer patients, surgical patients). A total of 34 studies used samples of individuals in other specific professions (e.g., military personnel), 32 used youth and adolescent samples (5–18 years old), 18 included older adults/aging populations, while 28 used samples in mental health care related professions (e.g., therapists). Nine studies used samples in other specific populations (e.g., spouses of depressed patients).

The number of possible psychometric assessments was 13 (see list below), and the total types of psychometric assessments reported for each measure ranged from 0 to 12. On average, each measure reported four types of psychometric assessments being completed. The measures with the highest number of psychometric assessments reported included the Interpersonal Reactivity Index (IRI) and the Self-Compassion Scale (SCS) with 12 psychometric assessments each. All scales with eight or more psychometric assessments reported in the articles we located can be found in S2 Table.

In regards to the type of psychometric assessments reported, a total of 409 studies assessed internal consistency, 342 used construct validity, 316 used factor analysis or principal component analysis, 299 assessed convergent validity, 218 used confirmatory factor analysis, 187 evaluated content validity, 165 tested for discriminant/divergent validity, 108 assessed test re-test reliability, 71 measured interrater reliability, 69 tested for predictive validity, 68 used structural equation modeling, 38 controlled for or examined correlations with social desirability, and 6 used a biased responding assessment or “lie” scale. Eighty studies performed other advanced statistics.

### Measures of empathy and compassion

A total of 503 measures of compassion and empathy were identified in the literature. S3 Table is sorted alphabetically by the name of the measure, and includes a description of each measure, year developed, type of measure, subscales (if applicable), administration time (if provided), number of items, sample items, and response set. The majority of the scales were developed in the past decade (since 2013). Most of the measures identified were self-report scales (412 scales). Fifty-three (53) were peer/corollary report measures (descriptions of target individuals’ thoughts, feelings, motives, or behaviors), and 38 were behavioral/expert coder measures (someone who has been trained to assess target’s thoughts, feelings motives or behaviors). There were 370 measures with subscales and 133 measures without subscales. The number of items of each scale varied widely from 1 item to 567 items. The average number of items was 32 (SD = 45.2) and the median was 21 items. Most authors did not report on the estimated time it would take to complete the measure.

### Interactive data visualization

**Fig 2:**
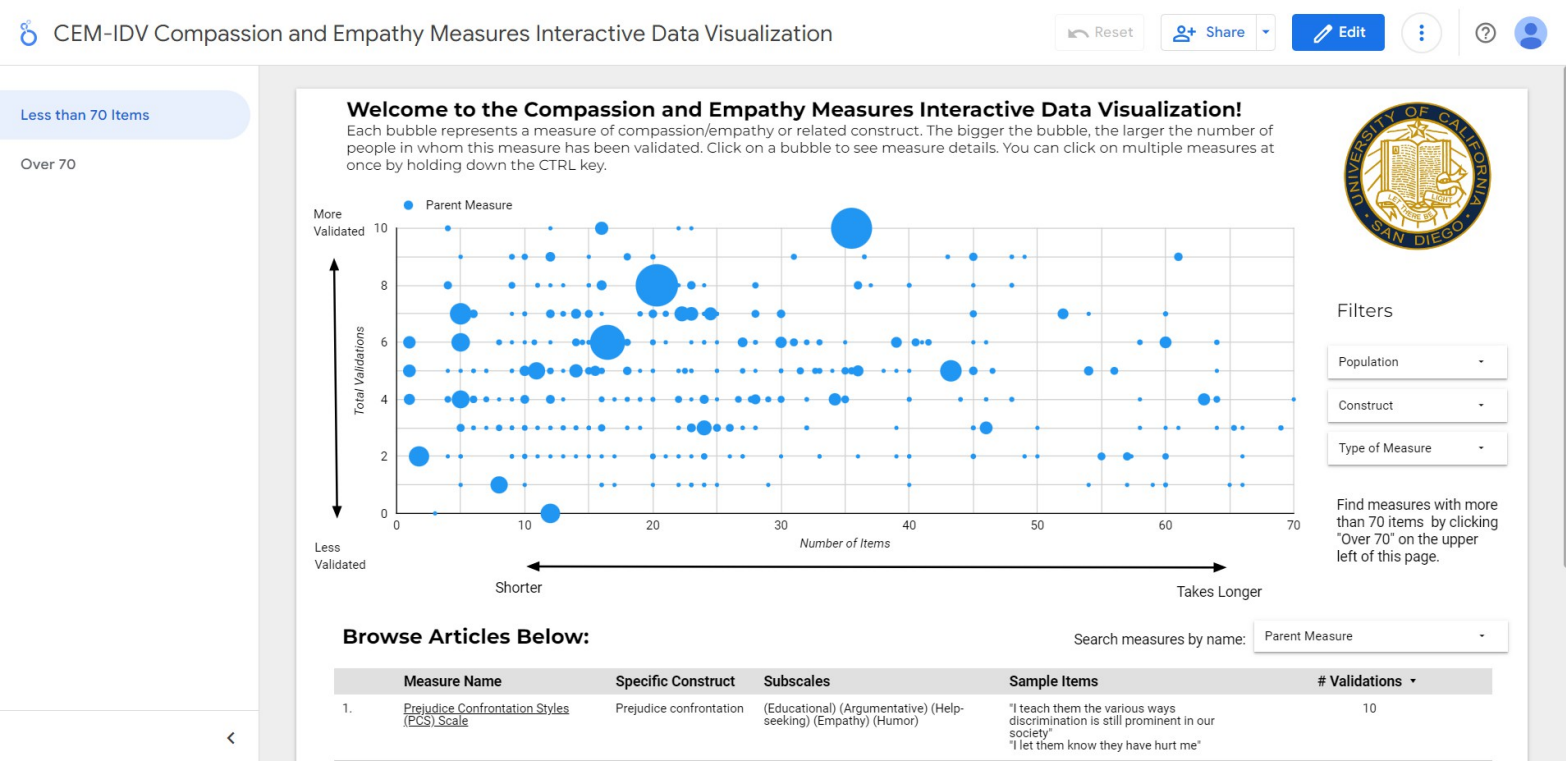
Interactive data visualization.

Data visualizations are graphical representations of data designed to communicate key aspects of complex datasets [48]. Interactive data visualizations allow users to search, filter, and otherwise manipulate views of the data, and are increasingly being used for healthcare decision making [49]. We used Google Data Studio to create an online open-access interactive data visualization (Fig 2) displaying the results of this scoping review. Access it at: https://imagination.ucsd.edu/compassionmeasures/

The purpose of this Compassion and Empathy Measures Interactive Data Visualization (CEM-IDV) is to assist health researchers and program evaluators in selecting appropriate measures of empathy and compassion based on a number of parameters, as well as learning more about how these constructs are currently being conceptualized. Visualization parameters include: number of types of psychometric assessments completed (1–12) on the y-axis, number of items on the x-axis (with measures with over 70 items appearing on a separate display, not shown in Fig 2), and the bubble size indicating the number of participants in the validation studies. Search filters include Population in which the measure has been validated (e.g. students, healthcare workers, general adults), Construct (e.g. empathy, compassion, caring, self-compassion), and Type of Measure (e.g. self-report, behavioral/expert coder). Users can also search measures by name of the parent measure. For example, there are multiple versions of the Jefferson Scale of Empathy (JSE) (e.g., for physicians, for nurses, for medical students). To retrieve all articles reporting on any version of the JSE, one would search for the parent measure (i.e., “Jefferson Scale of Empathy”). If a measure does not have multiple versions (for example, the Griffith Empathy Measure), this search would yield all articles on that single version.

## Discussion

A robust science of compassion and empathy relies on effective measures. This scoping review examined the broad literature of peer-reviewed published research articles that either developed, or assessed the psychometric properties of, instruments measuring compassion and empathy. The review also includes overlapping and related constructs such as self-compassion, theory of mind, perspective-taking, vicarious pain, caring, the doctor-patient relationship, emotional cues, sympathy, tenderness, and emotional intelligence.

Our review indicates that the field of measuring compassion and empathy is maturing. Strides have been made in recent years in conceptualization, definition, and assessment of compassion and empathy. Since the time of earlier critical reviews of measurement of compassion and empathy, several measures have gained more psychometric support: S2 Table shows that 34 measures have been subjected to 9 or more types of psychometric validation. Multiple measures in this review demonstrate consistent reliability and validity along with many other strengths.

Newer measures align more closely with experimental, theoretical and methodological advances in understanding the various components of compassion and empathy. For example, the newer Empathic Expressions Scale [50] recognizes that actual empathy *behaviors* are different from cognitive and affective aspects of empathy. In another example, increasing understanding of the role of warmth and affection as an important component of empathy has led to the development of the Warmth/Affection Coding System (WACS) [51]. That measure also includes both micro- and macro-social observations, recognizing that implicit and explicit behaviors are important for assessment.

As measurement becomes more precise, assessments have also reflected increasing understanding of the differences between compassion and empathy, and the interaction between the two. For example, the Compassion Scale [52] subscales include kindness, common humanity, mindfulness and indifference (reverse-scored), whereas the family of the Jefferson Scale(s) of Empathy include compassion as well as “standing in the patient’s shoes” and “understanding the client’s perspective.” Recognizing recent research on how compassion could temper consequences of empathic distress such as burnout, it becomes important for researchers and program evaluators to not only avoid conflating the two, but also measure both separately.

Empathy and compassion in specific circumstances for specific populations have also been developed, such as the Body Compassion Questionnaire [53] with clear relevance for adolescents and young adults, as well as those with eating and body-dysmorphic disorders, or the modified 5-Item Compassion Measure [54] created specifically for patients to assess provider compassion during emergency room visits.

In our review, we included self-report assessments, peer/corollary observational measures, and behavioral tasks/expert coder measures, for adults and children in English-speaking populations. A discussion of the utility of each of these types of measures follows, along with a rubric for measure selection that researchers and program evaluators can use with the assistance of the tables and/or CEM-IDV online tool.

### Self-report measures

The vast majority of measures of empathy and compassion are self-report measures (surveys, questionnaires, or items asking people to report on their own compassion and empathy). While perhaps the most efficient way to assess large numbers of participants, historically self-assessments of compassion and empathy have been riddled with challenges. Over a decade ago, Gerdes et al. [38] in their review of the literature noted that:

In addition to a multitude of definitions, different researchers have employed a host of disparate ways to measure empathy (Pederson, 2009). A review of the literature pertaining to empathy reveals that as a result of these inconsistencies, conceptualisations and measurement techniques for empathy vary so widely that it is difficult to engage in meaningful comparisons or make significant conclusions about how we define and measure this key component of human behaviour. (pp. 2327).

While a 2007 systematic review of 36 measures of empathy identified eight instruments demonstrating evidence of reliability, internal consistency, and validity [40], a systematic review of 12 measures of empathy used in nursing contexts [41] revealed low-quality scores (scoring 2–8 on a scale of 14), concluding that none of the measures were both psychometrically and conceptually satisfactory.

Our scoping review did not assess psychometric robustness other than the *number* of psychometric assessments completed, but a 2022 systematic review of measures of compassion [26] continued to reveal low-quality ratings (ranging from 2 to 7 out of 14) due to poor internal consistency for subscales, insufficient evidence for factor structure and/or failure to examine floor/ceiling effects, test-retest reliability, or discriminant validity. They concluded that “currently no psychometrically robust self- or observer-rated measure of compassion exists, despite widespread interest in measuring and enhancing compassion towards self and others” (pp. 26).

Several issues have been identified as potentially explaining shortcomings of compassion and empathy measures. For example, definitions of compassion and empathy vary widely in scholarly and popular vernacular, which can lead to variability in respondents’ perceptions. In addition to issues of semantics, the vast majority of compassion and empathy measures are face valid, relying on questions such as “I feel for others when they are suffering,” or “When I see someone who is struggling, I want to help.” These questions can increase the risk for social desirability bias (i.e., the tendency to give overly positive self-descriptions either to others or within themselves) and other response biases. Indeed, feeling uncompassionate can be quite difficult to admit, requiring not only a large degree of self-reflection and insight, but also an ability to manage the cognitive dissonance, shame, or embarrassment that could accompany such an admission. This difficulty may be particularly true among healthcare professionals.

Using self-report measures to assess the impact of compassion-focused interventions can also be confounded by mere exposure and demand characteristics, particularly when compared to standard-of-care or wait-list controls. In other words, after spending eight-weeks learning about and practicing compassion, it is not surprising that one might more frequently endorse items with respect to compassion due to increased familiarity with the concept, or implicit desire to satisfy experimenters, as opposed to increased compassionate states or behaviors. On the other hand, interventions could paradoxically result in people more accurately rating themselves *lower* on these outcomes once they investigate more thoroughly their own levels of, and barriers to, compassion and empathy, potentially masking improvements.

### Peer/corollary and behavioral/expert coder measures

With increasing technological, statistical, and conceptual sophistication, we can innovate new measures that can increase validity by triangulating more objective measures with self-perceptions. In fact, multiple measures using observation and ratings by peers, patients, or trained/expert behavioral coders have been developed to do just that. We identified 61 measures utilizing observational measures or peer/corollary reports, some involving a spouse, friend, supervisor, client or patient completing a questionnaire, rating form or checklist regarding their observations of that person. These measures may also include ratings of a live or recorded interaction by someone who has been trained to assess, or is an expert in assessing, compassion or empathy behaviors. Compassion or empathy behaviors include verbalizations and signals such as eye contact, tone of voice, or body language. Similarly, qualitative coding of transcribed narratives, interactions, or responses to interview questions or vignettes can be conducted with human qualitative coders, which is increasingly supported by artificial intelligence.

These methods have the clear benefit of avoiding self-report biases and providing richer data for each individual (for use in admissions or competency exams for instance). However, they can be labor intensive, can introduce potential changes in behavior due to knowing one is being observed, and can introduce another layer of subjectivity on the part of the observer/rater (which can be overcome in part by measures of agreement between two or more raters). They also tend to have fewer psychometric assessments testing their validity or reliability than other measures.

### Behavioral tasks

Laboratory-based behavioral tasks have been useful for assessing empathy and compassion under controlled conditions while reducing self-report biases and taking less time than qualitative/observational measures. These lab protocols involve exposure to stimuli designed to induce empathy and compassion or related constructs. For example, respondents might view a video-recorded vignette that reliably results in responses to seeing another person who is suffering [55] or write a letter to a prison inmate who has committed a violent crime [56]. Game theory has been used to create tasks focused on giving people options to share with, withhold from, or penalize others with cash, points, or goods. These are used to assess prosocial behaviors and constructs adjacent to empathy and compassion such as altruism and generosity [57].

The association of these implicit measures of compassion and empathy with real-world settings or with subjective perceptions of empathy and compassion is unknown. A meta-analysis of 85 studies (*N* = 14,327) indicates that *self-report* cognitive empathy scores account for only approximately 1% of the variance in *behavioral* cognitive empathy assessments [58]. This finding could demonstrate the superiority of implicit measures and a rather damning verdict for the accuracy of self-perceptions, or could imply that these different types of measures are capturing very different constructs (a problem that exists across many psychosocial versus behavioral measures, see [59]).

### Selecting measures

Our review revealed that there is not one or even a few measures of empathy and compassion that are best across all situations. Rather than providing overarching recommendations, therefore, we emphasize that measurement is context-dependent. As such, we recommend a series of questions researchers and program evaluators might ask themselves when selecting a measure.

We encourage readers to use the online CEM-IDV as a decision-aid tool to identify the best measure for their specific needs. To select the most appropriate instrument(s), we offer the following questions (in a suggested order) to provide guidance:

1. Which **precise domains** of empathy, compassion, or adjacent constructs do you want to measure? For example, is it the participant’s experience of empathy, or a skill or behavior? See the “General Construct” dropdown menu. Because definitions of empathy, compassion and related constructs are often imprecise, investigate whether the sample items, factors, and authors’ definition of the construct matches the outcome or variable you actually want to measure.
2. What **measurement type** is best suited to answering your research/evaluation question, or what is feasible for your setting and sample size? For example, if you have limited time or a large sample size, you may prefer a self-report survey, whereas if you are concerned about self-report bias, you might consider a direct observation or behavioral task/expert coder measure. Use S1 Table to examine measures by type of measure, or use the “Type of Measure” filter in the CEM-IDV.
3. What **measure length,** number of items, or time it takes to complete the assessment is feasible for the study? Refer to the X-axis of the CEM-IDV tool.
4. What **population**(s) are you working with? Use the population filter to explore whether the measures you are considering have been validated in those populations.
5. Do you want to **differentiate** the domain you are measuring **from other adjacent constructs**, such as sympathy or altruism, or distinguish between empathy and compassion? Select and include measures of each construct in order to make this distinction.
  Finally, now that you have selected several candidate measures, ask:
6. How **valid and reliable** is the measure? Use S1 Table or the Y-axis of CEM-IDV tool to determine which psychometric assessments have been completed, and click on the measure in the table below to review the full text of the papers to discover the strength of those assessments, as well as familiarizing oneself with the recent literature on the measure. Evidence for the validity, factor structure, or length of measures is often hotly debated, and it can be that a measure has been improved or its interpretation cautioned by recent literature.

### Use case

For example, imagine you are conducting a study of emergency room outcomes, including number of admissions, time from registration to discharge, and patient satisfaction. You would like to include emergency-room healthcare-provider empathy and/or compassion as a potential predictor or mediator of outcomes. After reviewing the literature on the topic and the definitions, you decide that compassion is the specific domain you are most interested in (Question 1). Because you are aware of the limitations of self-report measures, you decide not to use a self-report measure. You recognize that peer-reports, behavioral tasks, or expert coders are not appropriate for the fast-paced environment and number of interactions, but decide that patient reports of provider compassion would be ideal (Question 2). You recognize that the questionnaire must be brief, given the existing measurement burden and limited time participants have (Question 3). The population is emergency room clinicians and patients (Question 4). In this case, you are not interested in differentiating compassion from other similar constructs because that is not relevant to the question you are trying to answer: whether emergency room physician compassion predicts or mediates patient outcomes (Question 5).

In this case, you might use the CEM-IDV tool to select the population “Patients” and the construct “Compassion.” Your search yields eight potential measures, and upon reviewing each, you find that the 5-item Compassion Scale [54] has sample items that reflect what you are hoping to measure and was validated with emergency room patients and their clinicians. It demonstrates good reliability and validity and is an excellent choice for your project.

### Strengths and limitations

This scoping review has several strengths. First, it covers a wide breadth of literature on ways to assess empathy, compassion, and adjacent constructs using different types of measures (i.e., self-report, peer/corollary report, and behavioral/expert coder). Second, the findings were integrated into an accessible interactive data visualization tool designed to help researchers/program evaluators identify the most suitable measure(s) for their context. Third, the review team included individuals with expertise in conducting reviews, with the project manager having received formal training in best practices for systematic reviews, and an experienced data librarian helping to develop the search string and conduct the literature search. Fourth, the literature search was conducted without a start date limitation, thus capturing all measures published prior to October 2020. Fifth, the review team employed a comprehensive consensus process to establish study inclusion/exclusion criteria and utilized state-of-the-art review software, Covidence, to support the process of screening and data extraction.

There are also several limitations to consider. First, our literature search was limited to five databases (i.e., PubMed, Embase, PsychInfo, CINAHL, and Sociological Abstracts), and excluded grey literature, conference proceedings/abstracts, and measures not written in English. We also included only articles specifically focused on development and/or psychometric validation of measures. Thus, it is possible we missed relevant measures. Second, although we captured how frequently a measure was validated and the types of available psychometric evidence for each measure, we did not review the quality of the evidence. Measures with greater numbers of psychometric assessments may not necessarily be the most appropriate in all contexts or for particular settings, and psychometric studies can lead to conflicting results/interpretations. Importantly, the number of psychometric assessments might be skewed in favor of older measures that have existed in the scientific literature longer, and allegiance biases are possible. Thus, we reiterate that readers would benefit most from using the questions recommended above when selecting measures. Third, this scoping review provides a static snapshot of available measures through October 2020 and does not include measures that may have been published after that time.

Finally, the scoping review does not identify gold-standard measures to use. While systematic reviews typically include quality assessments, scoping reviews do not. Rather, scoping reviews seek to present an overview of a potentially large and diverse body of literature pertaining to a topic. As such, this review did not evaluate the quality of design, appraise the strength of the evidence, or synthesize reliability or validity results for each study. It may therefore include multiple studies that may have weak designs, low power, or evidence inadequate to the conclusions drawn.

## Conclusion

Given the multitude of problems facing society (e.g., violence and war, social injustices and inequities, mental health crises), learning how to cultivate compassion and empathy towards self and others is one of the most pressing topics for science to address. Furthermore, studies of compassion, empathy, and adjacent constructs rely on the use of appropriate measures, which are often difficult to select due to inconsistent definitions and susceptibility to biases. Our scoping review identified and reviewed numerous measures of compassion, empathy, and adjacent constructs, extracting the qualities of each measure to create an interactive data visualization tool. This tool is intended to assist researchers and program evaluators in searching for and selecting the most appropriate instruments to evaluate empathy, compassion, and adjacent constructs based on their specific context, setting, or population. It does not replace reviewers’ own critical evaluation of the instruments.

How a construct is measured reflects how it is being defined and conceptualized. Reviewing the subscales/factors and individual items that make up each measure sheds light on how each of these measures conceptualizes empathy and compassion. Ongoing research by our team is using these subscales, factors and items across measures to construct a conceptual map of compassion and empathy, which will be reported in a future paper. In the meantime, a useful feature of the CEM-IDV is that the list of articles yielded by searches includes subscales and sample items from each measure/article. These allow for a snapshot of how each measure or its authors have defined the constructs being assessed.

Future directions for measurement of empathy and compassion should consider incorporating advances in measurement and technology, and strive to bring together two or more assessment methods such as self-report, peer or patient reports, expert observation, implicit tasks, and biomarkers/physiological data to provide a more well-rounded picture of compassion and empathy. Innovations such as voice analysis and automated facial expression recognition may hold promise. Brief measures dispersed across multiple time points such as ecological momentary assessment and daily experience sampling may be useful. In conjunction with mobile technology and wearables, artificial intelligence and machine-learning data processing, could facilitate these formerly labor and time-intensive assessment methods.

## Supporting information

S1 Table Measure populations and psychometric assessments. (PDF)

S2 Table Measures with 8+ psychometric assessments. (PDF)

S3 Table Measures of compassion and empathy. (PDF)
