# Supplementary material for: Measures of empathy and compassion: A scoping review
Source: PLoS One. 2024 Jan 19;19(1):e0297099. doi: 10.1371/journal.pone.0297099 (PMC10798632; doi:10.1371/journal.pone.0297099)
Supplement: S1 Table — (PDF) [file pone.0297099.s001.pdf]

| Instrument | Reference | Study Type | Study Population | Age (range [R] and/or Mean [M] with Standard Deviation [SD]) | Gender | Internal Consistency | Test Re-Test Reliability | Interratter Reliability | Content Validity | Construct Validity | Discriminant/Divergent Validity | Convergent Validity | Predictive Validity | Factor Analysis/Principal Component Analysis | Confirmatory Factor Analysis | Structural Equation Modeling | Control/Correlation with Social Desirability | Other Advanced Statistics | Other Biased Responding or Lie Scale | Total Psychometric Assessments |
|---|---|---|---|---|---|---|---|---|---|---|---|---|---|---|---|---|---|---|---|---|
| **Behavioural/Expert Coder** | | | | | | | | | | | | | | | | | | | | |
| Affective Sensitivity Scale [Unnamed] | Campbell et al. (1971) | Behavioural/Expert Coder | 240 Master's-level counseling/guidance students | N/A | N/A | X | X | | X | X | | X | X | | | | | | | 6 |
| Analog Task of Prosocial Helping (AToP-H) | Greenslade & Coggins (2022) | Behavioural/Expert Coder | 20 children with autism spectrum disorder (ASD) and 20 with typical development | with ASD: 49-79 months; TD: 37-77 months | 28 male, 12 female | X | | X | X | X | X | X | | | | | | | | 6 |
| Assessment of Motivational Interviewing Groups – Observer Scales (AMIGOS) | Wagner & Ingersoll (2017) | Behavioural/Expert Coder | Adults in 18 group therapy sessions rated by experts (total number not reported) | | N/a | X | | X | | | | X | | | | | | | | 3 |
| Barrett-Lennard Relationship Inventory Observer Form (BLRI Obs-40) | Bhatti et al. (2023) | Behavioural/Expert Coder | 19 counselors and 69 young people | counelor: M=45, SD=9; young people: M=13.7, SD=.8 | counselors: 16% male, 84% female; young people: 30.4% male, 66.7% female | X | X | X | | X | | X | | X | X | X | | | | 8 |
| Behavioral Assessment of Empathy | Stover et al. (1971) | Behavioural/Expert Coder | 51 mother-child play sessions rated by researchers | Children: R: 4-10 | Female (100%) Male (0%) | | | X | | X | | X | | | | | | | | 3 |
| Callous-Unemotional Problems and Impairment (CUPI) | Castagna & Waschubusch (2021) | Behavioural/Expert Coder | 265 children ages 4 to 14 years old | M=8.04, SD=2.07 (range 4-14) | 67.9% boys | X | | | | X | X | X | | X | | | | | | 5 |
| Collaborative Behaviors Observational Assessment Tool (CBOAT) | Cayir et al. (2020) | Behavioural/Expert Coder | 33 interprofessional health care providers | | | | | X | X | | | | | | | | | | | 2 |
| Contingency Management Competence Scale (CMCS) | Petry et al. (2010) | Behavioural/Expert Coder | 35 substance abuse clinicians, 181 patients receiving substance abuse treatment (N=216) | | Female (48.2%) Male (51.8%) | X | | X | X | X | X | X | X | X | | | | | | 8 |
| Edinburgh Social Cognition Test (ESCoT) | Baksh et al. (2021) | Behavioural/Expert Coder | 19 adults with a diagnosis of Asperger's Syndrome (AS) or High-Functioning Autism (HFA) according to established DSM-IV criteria | M = 38.47, SD = 15.63 | 12 males, 7 females | X | | X | X | X | X | X | X | | | | | | | 7 |
| Empathetic Understanding Assessment (EUA) | Coyne et al. (2007) | Behavioural/Expert Coder | 128 mother-child dyads (N=256) | | Female (77%) Male (23%) | | | X | | | | | | | | | | | | 1 |
| Empathic Counselor Response Scale (ECRS) | Bayne & Hankey (2020) | Behavioural/Expert Coder | 290 counselors | R: 17-74 | 84.9% female, 14.8% male | X | | X | X | X | X | X | | X | X | | | | | 8 |
| Empathic Understanding Rating Scale | Avery & Danish (1976) | Behavioural/Expert Coder | 150 therapist-client interactions rated by trained individuals | | N/a | | | X | | | | | | | | | | | | 1 |
| Empathy and Clarity Rating Scale (ECRS) | Terregino et al. (2019) | Behavioural/Expert Coder | 65 medical students | | N/a | | | X | X | | | | | | | | | | | 2 |
| Empathy Clinical Evaluation Exercise (ECEX) | Stackhouse et al. (2020) | Behavioural/Expert Coder | 11 veterinary students rated by independent evaluators | | N/a | | | X | | X | | | | | | | | | | 2 |
| Essential Properties of Yoga Questionnaire (EPYQ) | Park et al. (2018) | Behavioural/Expert Coder | 481 yoga students, instructors, and researchers (Sample 1a), 329 active-duty military and veterans (Sample 1b), 491 yoga instructors (Sample 2), 144 yoga students, instructors, researchers and active-duty military and veterans (Sample 3, Phase IV) (N=1445) | | Sample 1a: Female (90%) Male (10%) Sample 1b: Female (50%) Male (50%) Sample 2: Female (92%) Male (8%) Phase 4: Female (79%) Male (21%) | X | X | X | X | X | X | X | | X | X | | | | | 9 |
| Four Habits Coding Scheme (4HCS) | Krupat et al. (2006) | Behavioural/Expert Coder | 50 physicians | | Female (50%) Male (50%) | X | | X | | X | | X | | | | | | | | 4 |
| Global Interprofessional Therapeutic Communication Scale© (GITCS©) | | Behavioural/Expert Coder | | | | X | | X | X | X | | | | X | X | | | X | | 7 |
| | Campbell et al. (2022) | Behavioural/Expert Coder | 426 nursing students | | | X | | | | | | | | X | X | | | | | 3 |
| | Campbell & Aredes (2019) | Behavioural/Expert Coder | 592 unique crowd source viewers | | N/a | | | X | X | X | | | | | X | | | X | | 5 |
| Health Coaching Index (HCI) | Sohl et al. (2021) | Behavioural/Expert Coder | 42 trainees who completed a National Board for Health and Wellness Coaching-approved training program and coached a standardized patient | not indicated | 41 female | | | X | X | X | | X | | | | | | | | 4 |
| Helpful Responses Questionnaire (HRQ) | Miller et al. (1991) | Behavioural/Expert Coder | 190 adults | | Female (80.5%) Male (19.5%) | | | X | | | | | | | | | | | | 1 |
| Helpful Responses to Refugees Questionnaire (HRRQ) | Potocky & L Guskovict (2016) | Behavioural/Expert Coder | 90 service providers working with refugees | | N/a | X | | X | X | X | X | X | | | | | | | | 6 |
| Jefferson Scale for Physician Empathy for Observers (JSEO) | Mallory et al. (2021) | Behavioural/Expert Coder | 4 raters, 24 videos of 12 interns | not indicated | not indicated | X | | X | X | X | | X | | | | | | | | 5 |
| Kids' Empathic Development Scale (KEDS) | Reid et al. (2013) | Behavioural/Expert Coder | 220 children | | Female (48%) Male (52%) | X | | | | X | X | X | | X | | | | | | 5 |
| Measure of Expressed Empathy | Malin & Pos (2015) | Behavioural/Expert Coder | 30 patients with depression and their therapists (n=16) | R: 18-65 | Female (73.9%) Male (26.1%) | X | | | X | X | | X | X | X | X | X | | | | 8 |
| Multiple Mini-Interview (MMI) | | | | | | X | | X | X | X | X | X | X | X | | | | X | | 9 |
| | Callwood et al. (2020) | Behavioural/Expert Coder | 225 students who were offered and accepted a place on one of the nursing or midwifery programmes | 18-35 | | | | X | | X | | X | X | | | | | | | 4 |
| | Cox et al. (2015) | Behavioural/Expert Coder | 253 pharmacy school candidates | M = 22.17, SD = 3.41 | Female (63.5%) Male (36.5% | | | X | | | | | | | | | | | | 1 |
| | Callwood et al. (2014) | Behavioural/Expert Coder | 62 midwife students | | N/a | X | | | X | X | | | | | | | | | | 3 |
| | Sebok et al. (2014) | Behavioural/Expert Coder | 444 medical students | | N/a | X | | X | | | | | | | | | | X | | 3 |
| | Hecker et al. (2009) | Behavioural/Expert Coder | 110 veterinary medicine applicants | | Female (79.1%) Male (20.9%) | X | | | X | X | X | | | X | | | | X | | 6 |
| Parent Affective and Cognitive Empathy Scale (PACES) | Stern et al. (2015) | Behavioural/Expert Coder | 60 primariy caregivers and their children (N=120) | R: 7-57, M = 23.35, SD = 4.05 | Female (90%) Male (10%) | | | X | X | X | X | X | | | | | | X | | 6 |
| Performance of Empathic Expression Rating Scale (PEERS) | Gagen et al. (2017) | Behavioural/Expert Coder | 60 individuals diagnosed with schizophrenia, 51 healthy adult controls (N=111) | | N/a | X | | | X | X | X | X | | | | | | | | 5 |
| Physician-Caregiver Relationship Scales (PCRS) | Cox et al. (2008) | Behavioural/Expert Coder | 96 physican-parent dyads (family medicine or pediatrician physicians and parents of a child patients) (N=192) | | | X | | X | X | X | X | | X | | X | | | X | | 8 |
| Quality of Interactions Schedule (QuIS) | Young et al. (2022) | Behavioural/Expert Coder | 107 patients | not indicated | not indicated | | | | X | | | | | | | | | | | 1 |
| Screening, Brief Intervention and Referral to Treatment Checklist for Observation in Real-time (SCORe) | Vendetti et al. (2017) | Behavioural/Expert Coder | 76 providers in emergency departments, hospital outpatient/ambulatory clinics, and hospital inpatient settings rated by trained individuals | | N/a | | | | X | | | | | | | | | | | 1 |
| Social Cognition and Functioning (SCAF) | Olbert et al. (2013) | Behavioural/Expert Coder | 173 patients with schizophrenia | | N/a | | X | X | | | X | | X | | | | | X | | 5 |
| Staff-Patient Interaction Response Scale for Palliative Care Nursing (SPIRS-PCN) | Yates et al. (1998) | Behavioural/Expert Coder | 181 nurses | | N/a | X | X | X | | | | | | | | | | | | 3 |
| Tape Excerpt Response Procedure (TERPs) | Wallston & Weitz (1975) | Behavioural/Expert Coder | 56 undergraduate counseling students | mean = 28.4 | Female (64.3%) Male (35.7%) | | X | X | | X | | | | | | | | | | 3 |
| Taxonomy of Compassionate Physician Behaviours [Unnamed] | Cameron et al. (2015) | Behavioural/Expert Coder | 49 interactions between oncologists (n=23) and patients with advanced cancer | 18+ | Female (52.6%) Male (47.4%) | | | X | | | | | | | | | | | | 1 |
| Therapist Empathy Scale (TES) | Decker et al. (2014) | Behavioural/Expert Coder | 91 therapists | | Female (65%) Male (45%) | X | | X | X | X | X | X | | | X | | | | | 7 |
| Therapists' Systemic Response Rating Scale [Unnamed] | Kung (2000) | Behavioural/Expert Coder | 15 clients in therapy for marital distress | | N/a | | | X | | X | | | X | | | | | | | 3 |
| Truax Accurate Empathy Scale (AE) | Wenegrat (1974) | Behavioural/Expert Coder | 30 clients of therapists (n=12) rated by trained undergraduate students | | Female (64.3%) Male (35.7% | | | X | | | | | | X | | | | | | 2 |
| Warmth/Affection Coding System (WACS) | Prasad et al. (2023) | Behavioural/Expert Coder | 2 expert coders | not indicated | N/A | X | | X | | X | | X | | | | | | | | 4 |
| **Peer/Corollary Report** | | | | | | | | | | | | | | | | | | | | 0 |
| 5-item Compassion Measure | Sabapathi et al. (2019) | Peer/Corollary Report | 866 emergency department patients | R: 18-93 | Female (55%) Male (45%) | X | | | | | | X | | | X | | | | | 3 |
| 24-Item Rating Form of Clinical Competence [Unnamed] | Hojat et al. (2007) | Peer/Corollary Report | 4560 medical college graduates | | Female (28%) Male (72%) | X | | | | | | X | | X | | | | | | 3 |
| Adapted Health Communication Assessment Tool© (adapted HCAT©) | Baird et al. (2021) | Peer/Corollary Report | 48 senior nursing students | N/A | N/A | X | | X | X | X | | | | X | | | | | | 5 |
| Adapted SERVQUAL | Asiamah et al. (2021) | Peer/Corollary Report | 520 patients | N/A | Female (46%) Male (53%) | X | | | X | X | X | X | | X | X | | | | | 7 |
| Altruism Scale | Friedman et al. (2015) | Peer/Corollary Report | 300 people who inject drugs | N/A | N/A | X | | | | X | | X | | | | | | | | 3 |
| ASPIRE Coaching Fidelity Checklist (ACFC) | Damschroder et al. (2016) | Peer/Corollary Report | 481 patients from Veterans Affairs medical centers enrolled in a weight management intervention trial | | N/a | X | | X | | X | | | | X | | | | X | | 5 |

| Instrument | Reference | Study Type | Study Population | Age (range [R] and/or Mean [M] with Standard Deviation [SD]) | Gender | Internal Consistency | Test Re-Test Reliability | Interratter Reliability | Content Validity | Construct Validity | Discriminant/Divergent Validity | Convergent Validity | Predictive Validity | Factor Analysis/Principal Component Analysis | Confirmatory Factor Analysis | Structural Equation Modeling | Control/Correlation with Social Desirability | Other Advanced Statistics | Other Biased Responding or Lie Scale | Total Psychometric Assessments |
|---|---|---|---|---|---|---|---|---|---|---|---|---|---|---|---|---|---|---|---|---|
| Augmented Stanford Faculty Development Program Questionnaire (SFDPQ) | Owolabi (2014) | Peer/Corollary Report | 84 medical residents and medical students | | Female (23.5%) Male (76.5%) | X | | | X | X | | | | X | | | | | | 4 |
| Beech Brook Therapy Assessment Instrument | Layman et al. (2013) | Peer/Corollary Report | 6 low-income children | R: 9-12 | Female (33.33%) Male (66.66%) | | | X | X | X | | | | | | | | | | 3 |
| Behavior Change Counseling Index (BECCI) | Lane et al. (2005) | Peer/Corollary Report | 51 adults in Motivational Interviewing (MI) consultations, ratings of 132 simulated MI consultations | | N/a | X | | X | | X | | | | | | | | | | 3 |
| Brief Patient Satisfaction Scale [Unnamed] | Hojat et al. (2011) | Peer/Corollary Report | 535 patients with at least two physician office visits in prior 3 years | | Female (66%) Male (44%) | X | | | X | X | | X | | X | | | | | | 5 |
| Caring Nurse–Patient Interaction Scale (CNPI) | Cossette et al. (2005) | Peer/Corollary Report | 332 nursing students | | Female (83%) Male (17%) | X | | | X | X | | X | | | | | X | | | 5 |
| Caring Nurse-Patient Interaction - Short Scale (CNPI-Short Scale) | Cossette et al. (2006) | Peer/Corollary Report | 377 nursing students | | Female (80%) Male (20%) | | | | X | X | | X | | | | | | | | 3 |
| Charge Nurse Support Scale (CNSS) | McGilton (2003) | Peer/Corollary Report | | | Female (84%) Male (16%) | X | X | | X | X | | | | X | | | | | | 5 |
| Child and Adolescent Dispositions Scale (CADS) | Mikolajewski et al. (2014) | Peer/Corollary Report | 686 twin pairs (MZ and DZ) (N=1372) | | Female (50.7%) Male (49.3%) | X | | | | | | | | | X | | | | | 2 |
| Children's Concentration and Empathy Scale (CCES) | Golino et al. (2021) | Peer/Corollary Report | 273 students, 15 teachers | M=5.11 | not indicated | X | | | X | | | | | X | X | X | | X | | 6 |
| Compassion Focused Therapy Therapist Competence Rating Scale (CFT-TCRS) | Horwood et al. (2020) | Peer/Corollary Report | 20 clinicians | | N/a | | | | X | | | | | | | | | | | 1 |
| Countertransference Management Scale (CMS) | Pérez-Rojas et al. (2017) | Peer/Corollary Report | 286 therapy supervisors | | Female (75.2%) Male (24.8%) | X | | | X | X | | X | | X | | | | | | 5 |
| Dental Home Scale (DHS) | Rozier et al. (2019) | Peer/Corollary Report | 795 mothers (Development Study), 781 mothers (Validation Study) (N=1576) | | N/a | X | | | | | | X | | X | X | | | | | 4 |
| Dietrich Object Relations and Object Representations Scale (DOROS) | Porcerelli & Dietrich (1994) | Peer/Corollary Report | 150 individuals who lost a parent to death | | Female (51%) Male (49%) | | | X | X | | X | X | | X | | | | | | 5 |
| Emotional Intimacy Scale (EIS) | Sinclair & Dowdy (2005) | Peer/Corollary Report | 90 women with rheumatoid arthritis | | Female (100%) Male (0%) | X | X | | X | X | | | | X | | | | | | 5 |
| Empathy Construct Rating Scale (ECRS) | | | | | | X | | | X | X | X | X | | X | | | | X | | 7 |
| | Layton & Wykle (1990) | Peer/Corollary Report | | | Female (88%) Male (12%) | | | | | X | | | | | | | | | | 1 |
| | La Monica (1981) | Peer/Corollary Report | 300 nurses and graduate nursing students | 20+ | Female (100%) Male (0%) | X | | | X | X | X | X | | X | | | | X | | 7 |
| Faculty Evaluation Instrument [Unnamed] | Colletti et al. (2010) | Peer/Corollary Report | 31 medical residents | | N/a | X | | | X | X | | | | | X | | | | | 4 |
| Global Rating of Empathy Scale (GRE) | O'Connor et al. (2014) | Peer/Corollary Report | 163 final-year medical students | | Female (53.4%) Male (46.6%) | | | X | X | | | | | | | | | | | 2 |
| Griffith Empathy Measure (GEM) | | | | | | X | X | X | | X | X | X | | X | | | | X | | 8 |
| | Murphy (2019) | Peer/Corollary Report | 3496 children | | N/a | | | | | X | | | | | | | | X | | 2 |
| | Dadds et al. (2008) | Peer/Corollary Report | 155 Australian parents and their children (N=310) | | Female (44%) Male (56%) | X | X | X | | X | X | X | | X | | | | | | 7 |
| Interpersonal Quality of Family Planning Scale (IQFP) | Dehlendorf et al. (2018) | Peer/Corollary Report | 346 women receiving contraceptive counseling | | Female (100%) Male (0%) | X | | | | X | X | X | X | | | | | | | 5 |
| Inventory of Callous-Unemotional (ICU) Parent-Report Form | McDonald et al. (2018) | Peer/Corollary Report | 291 mother–child dyads recruited from community based intimate partner violence services (N=582) | | Female (47%) Male (53%) | X | | | | X | | X | | X | | | | | | 4 |
| Inventory of Callous-Unemotional Traits (ICU) | Willoughby et al. (2015) | Peer/Corollary Report | 1078 first-grade children | | Female (50%) Male (50%) | | | | | | | | X | | X | X | | | | 3 |
| Jefferson Scale of Empathy for Observers (JSEO) | Mallory et al. (2020) | Peer/Corollary Report | 45 medical interns | | N/a | X | | X | | | | X | | | | | | | | 3 |
| Kiersma-Chen Empathy Scale Patient Scale (KCES-PV) | Blakely et al. (2022) | Peer/Corollary Report | 94 second-year student pharmacists | not indicated | 38% male | X | X | X | | X | | X | | X | | | | | | 6 |
| Measure of Empathy in Early Childhood (MEEC) | Kimonis et al. (2023) | Peer/Corollary Report | 1226 parents | 25-34 | 694 male, 532 female | X | | | | | | | | X | X | | X | | | 4 |
| Nurse 5-item Compassion Measure | Roberts et al. (2022) | Peer/Corollary Report | 4311 | M=63, SD=18 | 60% female | X | | | | X | X | X | | X | X | X | | | | 7 |
| Objective Structured Clinical Evaluation (OSCE) | | | | | | X | | | X | X | | | | | | | | X | | 4 |
| | Lu et al. (2011) | Peer/Corollary Report | 316 social workers | | N/a | X | | | | | | | | | | | | X | | 2 |
| | Hodges & McIlroy (2003) | Peer/Corollary Report | 57 medical students | | N/a | X | | | X | X | | | | | | | | | | 3 |
| Observed Mindfulness Measure (OMM) | Bartlett et al. (2022) | Peer/Corollary Report | 681 adults | R: 18-65+ | 76% female | X | X | X | X | X | X | X | | X | X | | | | | 9 |
| Patient Centeredness Index (PCI) | Rose et al. (2022) | Peer/Corollary Report | 3622 patients | M=62, SD=13 | 63% female, 37% male | X | | | X | X | | X | | X | | | | | | 5 |
| Patient's Assessment of Quality Scale - Acute Care Version (PAQS-ACV) | Lynn et al. (2007) | Peer/Corollary Report | 1470 patients | | Female (50%) Male (50%) | | X | | X | X | | X | | X | | | | | | 5 |
| Peer Group Caring Interaction Scale (PGCIS) | Hughes (1998) | Peer/Corollary Report | 853 undergraduate nursing students | | Female (90.5%) Male (9.5%) | X | | | X | X | X | | | | | | | X | | 5 |
| Perceived Empathy Measure | Plank et al. (1996) | Peer/Corollary Report | 111 purchasing agents (Development Study), 568 purchasing agents (Validation Study) (N=679) | | Female (66.5%) Male (33.5%) | X | | | X | X | | | X | X | X | | | - | | 6 |
| Physician 5-item Compassion Measure | Roberts et al. (2022) | Peer/Corollary Report | 4240 | M=63, SD=18 | 60% female | X | | | | X | X | X | | X | X | X | | | | 7 |
| Schwartz Center Compassionate Care Scale (SCCCS) | | | | | | X | X | | X | X | | X | | X | X | | | X | | 8 |
| | Rodriguez & Lown (2019) | Peer/Corollary Report | 23 patients (Qualitative Study), 167 patients (Quantitative Study) (N=190) | | Psychometric sample: Female (74.7%) Male (25.3%)<br>Cognitive debriefing sample: Female (34.8%) Male (65.2%) | X | X | | X | X | | X | | X | | | | X | | 7 |
| | Lown et al. (2015) | Peer/Corollary Report | 801 patients who had been recently hospitalized for at least three days | | N/a | X | | | | | | X | | X | X | | | X | | 5 |
| | Lown et al. (2017) | Peer/Corollary Report | 501 recently hospitalized and non-hospitalized adults in Ireland and the U.S. | | Female (25.3%) Male (3.8%) Other (70.9%) | X | X | | | | | | | X | | | | X | | 4 |
| Screening Tool for Care Coordinator Assistants | Cottingham et al. (2014) | Peer/Corollary Report | 62 care coordinator assistant candidates | | Female (94%) Male (6%) | | | | X | X | | | X | | | | | | | 3 |
| Security of "I" Assessment (SofIA) | Huang & Berenbaum (2017) | Peer/Corollary Report | 195 undergraduate students (Study 1), 158 undergraduate students (Study 2) (N=353) | | Study 1: Female (61.3%) Male (38.7%)<br>Study 2: Female (69.3%) Male (30.7%) | | X | | | | | | | X | | | | | | 2 |
| Self-Care Counseling Rubric (SCCR) | Chen et al. (2019) | Peer/Corollary Report | 47 pharmacy students | | N/a | X | | X | | | | | | | | | | | | 2 |
| SERVPERF-R | Fogarty et al. (2000) | Peer/Corollary Report | 113 customers after a purchase at a retail store (Development Study), 212 customers after a purchase at a retail store (Validation Study) (N=325) | | N/a | X | | | | X | | | | | X | X | | | | 4 |
| Sinclair Compassion Questionnaire (SCQ) | Sinclair et al. (2021) | Peer/Corollary Report | phase 1: 303 patients<br>phase 2: 330 patients | phase 1: 73 ± 14 years<br>phase 2: 74 ± 14 years | phase 1: 180 (59·4%) female; 123 (40·6%) male<br>phase 2: 182 (55·0%) female; 148 (44·8%) male | X | X | | X | X | X | X | | X | X | | | | | 8 |
| Social Competence Interview | Ewart et al. (2002) | Peer/Corollary Report | 130 high school students | | N/a | X | X | | | X | | X | | | | X | | | | 5 |
| Social-Emotional Assets and Resilience Scale - Parent Form (SEARS-P) | Merrell et al. (2011) | Peer/Corollary Report | 2356 parents/guardians of children and adolescents | Children and adolescents ages 5-19 | Female (41%) Male (59%) | X | | X | | X | | X | | X | X | | | | | 6 |
| Social Skills Imporvement System Rating Scales Parent Form (SSiS) | Channell et al. (2023) | Peer/Corollary Report | 124 children with down syndrome | M = 11.61, SD =3.48 | 47.6% male, 52.4% female | | | | | X | X | X | | | | | | | | 3 |
| Solidarity Scale | Friedman et al. (2015) | Peer/Corollary Report | 300 people who inject drugs | | N/a | X | | | | X | | X | | | | | | | | 3 |
| Survey of Physician Humanistic Approaches to Patient Care | Glaser et al. (2007) | Peer/Corollary Report | | | Female (64%) Male (36%) | | | | | | X | X | | | | | | | | 2 |
| Unit Manager Support Scale (UMSS) | McGilton (2003) | Peer/Corollary Report | | | Female (84%) Male (16%) | X | X | | X | X | | | | X | | | | | | 5 |
| Visual CARE Measure 5Q | Place et al. (2016) | Peer/Corollary Report | 149 children, 220 parents (N=369) | | Children: Female (35%) Male (65%)<br>Parents: Female (59%) Male (41%) | X | | | X | | | | | | | | | | | 2 |

| Instrument | Reference | Study Type | Study Population | Age (range [R] and/or Mean [M] with Standard Deviation [SD]) | Gender | Internal Consistency | Test Re-Test Reliability | Interratter Reliability | Content Validity | Construct Validity | Discriminant/Divergent Validity | Convergent Validity | Predictive Validity | Factor Analysis/Principal Component Analysis | Confirmatory Factor Analysis | Structural Equation Modeling | Control/Correlation with Social Desirability | Other Advanced Statistics | Other Biased Responding or Lie Scale | Total Psychometric Assessments |
|---|---|---|---|---|---|---|---|---|---|---|---|---|---|---|---|---|---|---|---|---|
| Visual CARE Measure 10Q | Place et al. (2016) | Peer/Corollary Report | 149 children, 220 parents (N=369) | | Children: Female (35%) Male (65%)<br>Parents: Female (59%) Male (41%) | X | | | X | | | | | | | | | | | 2 |
| Visual CARE Measure 10Q Parent | Place et al. (2016) | Peer/Corollary Report | 149 children, 220 parents (N=369) | | Children: Female (35%) Male (65%)<br>Parents: Female (59%) Male (41%) | X | | | X | | | | | | | | | | | 2 |
| **Self-Report** | | | | | | | | | | | | | | | | | | | | 0 |
| 1-Item Brief Burnout Screening [Unnamed] | Kemper et al. (2019) | Self-Report | 3906 pediatric residents | | Female (72%) Male (28%) | | | | | X | | | | | | | | | | 1 |
| 2-Item Brief Burnout Screening [Unnamed] | Kemper et al. (2019) | Self-Report | 3906 pediatric residents | | Female (72%) Male (28%) | | | | | X | | | | | | | | | | 1 |
| 6-item brief short form Coping with Children's Negative Emotions Scale (CCNES) | King et al. (2022) | Self-Report | 938 parents | M=37.37, SD=6.9 | 556 female, 341 male, 2 non-binary/transgender | X | X | | X | X | | X | | X | X | X | | X | | 9 |
| 10-item Remembered Relationship with Parents (RRP10) Scale | Denollet et al. (2007) | Self-Report | 664 adults | | Female (52%) Male (48%) | X | | | | | | X | | X | | | | | | 3 |
| 15-item Mutualism Scale | Lin & Bates (2022) | Self-Report | 902 participants (study 1: 400; study 2: 502) | study 1: M=40, SD=12.54; study 2: M=36, SD=12.12 | study 1: 252 females, study 2: 381 females | X | X | | | X | X | | X | X | X | X | | | | 8 |
| 18-item short form Coping with Children's Negative Emotions Scale (CCNES) | King et al. (2022) | Self-Report | 937 parents | M=37.37, SD=6.8 | 556 female, 341 male, 2 non-binary/transgender | X | X | | X | X | | X | | X | X | X | | X | | 9 |
| 20-Item Toronto Alexithymia Scale (TAS-20) | Carnovale et al. (2021) | Self-Report | 1930 adults | M= 35.46, SD 12.55 (range 18-81) | 1063 women, 865 men | X | | | X | X | X | X | | X | X | | | | | 7 |
| 30-Item Rating Scale of Parent Satisfaction with Pediatric Neuropsychological Evaluations [Unnamed] | Bodin et al. (2007) | Self-Report | 117 parents/guardians of children at a children's hospital | | Patients: Female (45%) Male (55%) | X | | | X | X | X | X | | X | | | | | | 6 |
| Accountability Scale | Witvliet et al. (2022) | Self-Report | 1257 participants (study 1: 484 adults, study 2: 773 adults, study 3: 234 adults) | study 1: M=46.66, SD=17.23; study 2: M=44.42, SD=16.52; study 3: M=46.43, SD=16.32 | study 1: 241 female, 242 male, 1 other; study 2: 389 female, 376 male, 5 other; study 3: 131 female, 101 male, 2 other | X | X | | X | X | X | X | | X | X | | | | | 8 |
| Actions and Feelings Questionnaire (AFQ) | Williams et al. (2016b) | Self-Report | 278 adults (Study 1), 20 right-handed adults (Study 2) (N=298) | | Study 1: Females (68%) Males (32%)<br>Study 2: Females (50%) Males (50%) | X | X | | | | | X | | X | | | | | | 4 |
| Active Empathetic Listening (AEL) Scale | | | | | | X | | | | X | X | X | | X | X | X | | X | | 8 |
| | Gearhart et al. (2011) | Self-Report | 345 undergraduate students | | Female (55%) Male (45%) | X | | | | X | X | X | | | X | | | | | 5 |
| | Drollinger et al. (2006) | Self-Report | 151 salespeople | | Female (23%) Male (77%) | | | | | X | | X | | X | X | X | | X | | 6 |
| Ad Response Empathy (ARE) | Escalas & Stern (2003) | Self-Report | 22 undergraduate students | N/A | N/A | X | | | | X | | | | | | X | | X | | 4 |
| Ad Response Sympathy (ARS) | Escalas & Stern (2003) | Self-Report | 22 undergraduate students | N/A | N/A | X | | | | X | | | | | | X | | X | | 4 |
| Adolescent and Adult Mindfulness Scale (AAMS) | Droutman et al. (2018) | Self-Report | 588 older adolescents | R: 18-25 | Female (79%) Male (21%) | X | X | | | X | | X | X | | X | | | X | | 7 |
| Adolescent Attitudes towards Dementia Scale (A-ADS) | Griffiths et al. (2018) | Self-Report | 262 high school students | R: 14-18 | Female (59%) Male (41%) | X | | | X | X | | X | | X | | | | | | 5 |
| Adult-Adolescent Parenting Inventory (AAPI) | Lutenbacher (2001) | Self-Report | 225 low-income single mothers | | Female (100%) Male (0%) | X | | | | X | X | X | | X | | | X | | | 6 |
| Affective and Cognitive Measure of Empathy (ACME) | | | | | | | X | | | X | X | | X | | | X | X | | | 6 |
| | Murphy et al. (2020) | Self-Report | | | Female (53%) Male (47%) | | X | | | X | X | | X | | | X | X | | | 6 |
| | Vachon & Lynam (2016) | Self-Report | 1287 undergraduate psychology students | | Female (48.5%) Male (51.5%) | | X | | | | | | | | | X | | | | 2 |
| Affective Forecasting Situation Questionnaire | Qian et al. (2020) | Self-Report | 272 medical students | | Female (75.7%) Male (24.3%) | X | | | X | | | X | | | | | | | | 3 |
| AIDS Attitude Scale | Froman & Owen (1997) | Self-Report | 21 practicing nurses (Study 1), 64 practicing nurses (Study 2) (N=85) | N/A | N/A | X | | | | X | X | | | | | | | | | 3 |
| AIDS Attitude Scale-Generic (AAS-G) | Froman & Owen (2001) | Self-Report | 318 adults | | Female (57%) Male (43%) | X | X | | X | X | | | | X | | | | | | 5 |
| Altruism Scale for Children (ASC) | Swank et al. (2020) | Self-Report | 576 elementary to high school students | R: 8-19, M = 11.52, SD = 2.58 | Female (35%) Male (62%) Other (3%) | X | X | | | X | | X | | X | X | | | | | 6 |
| Anasakti Scale [Unnamed] | Singh & Raina (2015) | Self-Report | 366 undergraduate, graduate, and research students in India | M = 23.55 | Female 64.5%, Male 35.5% | | | | | | | X | | X | | | | | | 2 |
| Appreciative Joy Scale (AJS) | Zeng et al. (2017) | Self-Report | 631 Chinese adults (Study 1), 281 Chinese adults (Study 2), 294 Chinese adults (Study 3), 255 American adults (Study 4), 301 Chinese undergraduate students (Study 5) (N=1762) | | Females (66.2%) Male (33.8%) | X | X | | X | X | | | | | X | | X | X | | 7 |
| Arab Muslim Perceptions of Perinatal Loss Care (AMPPLC) | Alaradi et al. (2022). | Self-Report | 79 Arab Muslim adults | M = 40.3 years, SD = 13.5 | Male (54.4%), female (45.6%) | X | | | | | | | | | | | | | | 1 |
| Arble Estimate of Selfobject Pursuits (AESOP) | Arble & Barnett (2017) | Self-Report | 738 undergraduate students | | Female (71%) Male (29%) | X | | | X | X | X | X | | X | X | | X | X | | 9 |
| ASP ["Expressions of Spirituality"] Questionnaire | Büssing et al. (2007) | Self-Report | 488 German hospital staff members | M = 49.2, SD = 13.9 | Female (63%) Male (37%) | X | | | X | X | | | | X | | | | | | 4 |
| Attitudes About Reality Scale (AAR) | Evans (2000) | Self-Report | 118 employees of a mental health agency | R: 21-81 | Female (75.4%) Male (24.6%) | | | | | X | | | | | | | | | | 1 |
| Attitudes Related to Trauma-Informed Care Scale (ARTIC) | Baker et al. (2021) | Self-Report | 1395 human services/health providers | R: 18-65+ | Female = 565<br>Male = 175<br>Other = 2<br>Transgender = 1 | X | X | | X | X | X | X | X | X | X | | | | | 9 |
| Attitudes toward Suicidal Patients | Aish et al. (2002) | Self-Report | 1010 medical staff members | | Female (71.8%) Male (28.2%) | | | | | | | | | | X | X | | | | 2 |
| Attitudes Toward Women with HIV/AIDS Scale | O'Hea et al. (2001) | Self-Report | 225 undergraduate psychology students | | Female (81%) Male (19%) | X | | | | X | | | | X | | | | | | 3 |
| Attribution Questionnaire-Substance Use Disorder (AQ-SUD) | Johnson-Kwochka et al. (2021) | Self-Report | 304 participants | M=37.2, SD=10.97 | 61.5% male | X | | | X | X | X | X | | X | | | | | | 6 |
| Awareness, Knowledge, Skills - General (ASK-G) | Domenech et al. (2022) | Self-Report | Phase 1: 13 experts, Phase 2: 204 adults | M=41.63, SD = 16.08 | 45.59% male, 50% female | X | | | X | X | X | X | | X | X | X | | | | 8 |
| Barrett-Lennard Relationship Inventory (BLRI) | Cramer (1986) | Self-Report | 335 undergraduate and graduate students | | Female (71.6%) Male (28.4%) | | | | | | | | | X | | | | X | | 2 |
| Barrett-Lennard Relationship Inventory - Empathy (BLRI-E) | Layton & Wykle (1990) | Self-Report | | | Female (88%) Male (12%) | | | | | X | | | | | | | | | | 1 |
| Barrett-Lennard Relationship Inventory for Receiver (B-L RI: OS-64) | Chen et al. (2022) | Self-Report | 1286 participants (658 native Chinese speakers, 330 native Spanish speakers, 298 nativ English speakers) | M=38, SD = 12.9 | (English speaking sample) 87.6% female, 12.1% male | | | | X | | | | | X | X | X | | X | | 5 |
| Barriers to Physician Compassion Questionnaire | Fernando & Consedine (2014) | Self-Report | 75 physicians | | N/a | X | | | | X | | X | | | | X | | X | | 5 |
| Basic Empathy Scale (BES) | | | | | | X | | | | X | X | X | | X | X | | | | | 6 |
| | McLaren et al. (2019) | Self-Report | 555 adolescents | | Female (64.1%) Male (35.9%) | | | | | X | | | | X | | | | | | 2 |
| | Peretti et al. (2019) | Self-Report | | | | | | | | | X | X | | X | | | | | | 3 |
| | Jolliffe & Farrington (2006) | Self-Report | 363 adolescents | M = 14.8 | Female (46.6%) Male (53.4%) | X | | | | X | | | | X | X | | | | | 4 |
| Body Compassion Questionnaire (BCQ) | Beadle et al. (2021) | Self-Report | study 1: 728 individuals, study 2: 27 female psychology students, 45 female psychology students | study 1: M = 28.38, SD = 11.92; study 2: M=21.88; study 3: M=27.87, SD=13.44 | study 1: 127 males, 592 females, 9 other/rather not say; study 2: 27 females; study 3: 45 females | X | X | X | X | X | X | X | | X | X | X | | | | 10 |
| Body Compassion Scale (BCS) | | | | | | X | | | X | X | | X | | X | X | | X | X | | 8 |

| Instrument | Reference | Study Type | Study Population | Age (range [R] and/or Mean [M] with Standard Deviation [SD]) | Gender | Internal Consistency | Test Re-Test Reliability | Interratter Reliability | Content Validity | Construct Validity | Discriminant/Divergent Validity | Convergent Validity | Predictive Validity | Factor Analysis/Principal Component Analysis | Confirmatory Factor Analysis | Structural Equation Modeling | Control/Correlation with Social Desirability | Other Advanced Statistics | Other Biased Responding or Lie Scale | Total Psychometric Assessments |
|---|---|---|---|---|---|---|---|---|---|---|---|---|---|---|---|---|---|---|---|---|
| | Van Niekerk et al. (2023) | Self-Report | 513 women | M = 29.84, SD = 10.88 | 100% women | X | | | | X | | X | | X | X | | | | | 5 |
| | Altman et al. (2020) | Self-Report | study 1: 662 undergraduates<br>study 2: 256 undergraduates | study 1: 20.49 mean age, SD=3.86 years<br>study 2: 24.26 mean age, SD=4.16years | study 1: Female (70.8%)<br>study 2: Female (72.1%) | X | | | | | | | | X | X | | | | | 3 |
| | Altman et al. (2017) | Self-Report | 662 undergraduate students (Study 1), 256 undergraduate psychology students (Study 2) (N=918) | | Female (71.2%) Male (28.8%) | X | | | X | X | | X | | X | X | | X | X | | 8 |
| Body Image–Acceptance and Action Questionnaire (BIAAQ) | Basarkod et al. (2018) | Self-Report | 538 adults (Sample 1), 762 adults (Sample 2) (N=1300) | | Female (55.7%) Male (44.3%) | X | | | | X | | | | X | | | | | | 3 |
| Body-Image Contextualized Adaptation of the Self-Compassion Scale (BI-SCS) | Ullrich-French & Cox (2022) | Self-Report | 467 students | M = 20.4, SD = 4.86 | 68% female, 31% male | X | | | | X | | X | X | X | X | | | | | 6 |
| Bolton Compassion Strengths Indicators (BCSIs) | Durkin et al. (2020) | Self-Report | 421 preregistration undergraduate nursing students | M = 29, SD = 1=8.3 | 90.7% female | X | X | | X | X | X | X | X | X | X | | | | | 9 |
| Breaking Bad News (BBN) Assessment Tool [Unnamed] | Vermylen et al. (2019) | Self-Report | 10 medical students | | Female (0%) Male (100%) | | | X | | | | | | | | | | X | | 2 |
| Brief Assessment of Social Skills - Dementia (BASS-D) | Kelly & McDonald (2020) | Self-Report | 26 diagnosed with dementia, 28 healthy older adults an (N=54) | M = 81.38, SD = 9.39 | N/a | | | | | X | X | X | | | | | | | | 3 |
| Brief Dimensional Apathy Scale (b-DAS) | Radakovic et al. (2020) | Self-Report | 53 ALS patients, 53 caregivers/partners/relatives (N=106) | | N/a | X | X | | | | X | X | | | | | | | | 4 |
| Burnout Measure - Short Version | Ahmed et al. (2022) | Self-Report | 375 physicians | 52.43 mean age | Female (42.4%) | | | | | X | X | | | X | X | | | | | 4 |
| Burns-Specific Patient-Reported Experience Measure (PREM) [Unnamed] | Hodgkinson et al. (2019) | Self-Report | 27 burn patients | | N/a | X | | | | | | | | | | | | | | 1 |
| Calm Compassionate Care Scale (CCCS) | Kemper et al. (2015) | Self-Report | 213 healthcare professionals | | Female (73%) Male (27%) | X | | | | | | X | | | | | | | | 2 |
| Care Receiver View of Caregiver Role Enactment Scale | Tetz et al. (2006) | Self-Report | 123 patient-caregiver dyads (N=246) | | Female (65%) Male (35%) | | | | X | X | | | | X | | | | | | 3 |
| Caring Assessment for Care Givers | Steele-Moses et al. (2011) | Self-Report | 54 nurses | | N/a | X | X | | X | X | X | X | | X | | | | | | 7 |
| Caring Assessment Tool (CAT) | Duffy et al. (2007) | Self-Report | 557 hospitalized adults | | N/a | X | | | X | | | | | | | | | | | 2 |
| Caring Assessment Tool - Version V (CAT-V) | Sim et al. (2019) | Self-Report | 476 nurses | | N/a | X | | | | X | | X | | X | X | | | | | 5 |
| Caring Behavior Coding Scheme (CBCS) | Mårtensson et al. (2021) | Self-Report | 19 undergraduate nursing students | range: 21-31 | 4 men, 15 women | X | | X | X | | | | | | | | | | | 3 |
| Caring Behaviors Inventory (CBI) | Coulombe et al. (2002) | Self-Report | 211 hospital patients discharged from a surgical unit | | Female (49.6%) Male (50.4%) | X | | | | | | X | | | | | | X | | 3 |
| Caring Behaviors Inventory - Short Form (CBI-24) | Wu et al. (2006) | Self-Report | 362 hospitalized patients (Study 1), 64 patients and 42 nurses (Test-Retest Study) (N=468) | | Female (60.6%) Male (39.4%) | X | X | | | X | | X | | X | | | | | | 5 |
| Caring Culture Survey | Mensik et al. (2019) | Self-Report | 640 direct and non-direct care staff | | N/a | X | | | | | | | | | X | | | | | 2 |
| Caring Dimensions Inventory (CDI) | | | | | | X | | X | X | X | | | | X | X | X | | | | 7 |
| | Watson (2003) | Self-Report | 38 nursing lecturers | | N/a | | | X | | | | | | | | | | | | 1 |
| | Lea et al. (1998) | Self-Report | 1452 nurses and nursing students | | Female (86.4%) Male (13.6%) | | | | | X | | | | X | X | X | | | | 4 |
| | Watson & Lea (1997) | Self-Report | 1430 nurses | | Female (88.8%) Male (11.2%) | X | | | X | X | | | | | | | | | | 3 |
| Caring Dimensions Inventory - 35-Item Version (CDI-35) | Watson et al. (2001) | Self-Report | 168 Scottish nursing students | | N/a | X | | | | | | | | X | X | | | | | 3 |
| Caring Efficacy Scale (CES) | Reid et al. (2015) | Self-Report | 2000 nurses | | N/a | X | | | | X | | | | X | X | | | | | 4 |
| Caring for Bliss Scale | Rudaz et al. (2020) | Self-Report | 205 adults (Study 1), 692 adults (Study 2) (N=897) | | | X | X | | | | | X | | | X | | | | | 4 |
| Caring Scale for Institutionalized Elders | Hwang et al. (2017) | Self-Report | 8 experts (Study 1a, content validity), 11 institution-dwelling older adults (Study 1b, item analysis), 300 institution-dwelling older adults (Study 1c, EFA), 216 institution-dwelling older adults (Study 1d, CFA), 216 institution-dwelling older adults (Study 2) (N=751) | R: 60-97 | 1. N/a<br>2. N/a<br>3. Female (60.3%) Male (39.7%)<br>4. Female (54.2%) Male (45.8%) | X | X | | X | | | X | | X | X | | | | | 6 |
| Carkhuff Scale | Layton & Wykle (1990) | Self-Report | | | Female (88%) Male (12%) | | | | | X | | | | | | | | | | 1 |
| Carolina Opinions on Care of Older Adults (COCOA) | Hollar et al. (2011) | Self-Report | 160 medical students | R: 21-34 | Female (50.5%) Male (49.5%) | X | | | X | X | | X | | X | | X | | | | 6 |
| Child Care Worker Interview | Ross & Hoeltke (1985) | Self-Report | 95 child care workers | | N/a | | | | X | | | X | X | | | | | | | 3 |
| Childhood Narcissism Scale (CNS) | Thomaes et al. (2008) | Self-Report | 300 Dutch children (Study 1), 1020 Dutch children (Study 2), 49 American children (Study 3), 302 Dutch children (Study 4), 238 Dutch children (Study 5), 280 Dutch children (Study 6) (N=2189) | R: 8-14 | 6 studies:<br>M (51%) F (49%)<br>M (51%) F (49%)<br>M (53%) F (47%)<br>M (57%) F (43%); M (54%) F (46%)<br>M (47%) F (53%)<br>M (55%) F (45%) | X | X | | | X | | | | | X | | X | | | 5 |
| Child-Related Values Survey (CRVS) | Sockloskie (1990) | Self-Report | 436 undergradudate psychology students | | Female (20%) Male (80%) | X | | | | X | X | X | | X | X | X | X | | X | 9 |
| Clinical Skills Assessment (CSA) | van Zanten et al. (2005) | Self-Report | 7746 doctors | M = 30.5, SD = 5.3 | Female (42%) Male (58%) | | | | | | | | | | | | | | | 0 |
| Clinician and Group Consumer Assessment of Healthcare Providers and Systems Survey (CG-CAHPS) | Roberts et al. (2019) | Self-Report | 6493 patients from an outpatient clinic | M = 60, SD = 15 | Female (65.3%) Male (34.7%) | X | | | | | | X | | X | X | | | | | 4 |
| Clinician Associative Stigma Scale (CASS) | Yanos et al. (2017) | Self-Report | 472 clinicians who work with people with serious mental illnesses | | Female (72%) Male (26%) Other (1%) | X | | | | X | X | X | | X | | | | | | 5 |
| Cognitive, Affective, and Somatic Empathy Scale (CASES) | | | | | | X | | | | X | X | X | X | X | X | | | | | 7 |
| | Raine et al. (2022) | Self-Report | 2604 community participants | M=36.95, SD=11.54 | 51.5% female | X | | | | X | X | X | | X | X | | | | | 6 |
| | Raine & Chen (2018) | Self-Report | 428 children | R: 11-12 | N/a | X | | | | X | X | X | X | | | | | | | 5 |
| Compassion Competence Scale (CCS) | Gordon et al. (2022) | Self-Report | 35 aged care workers | not indicated | not indicated | X | X | X | X | | | | | | | | | | | 4 |
| Compassion Fatigue Self-Test (CFST) | Jenkins & Baird (2002) | Self-Report | 104 sexual assault and/or domestic violence counselors | | N/a | X | | | | X | X | X | | X | | | | | | 5 |
| Compassion Motivation and Action Scales (CMAS) for Compassion | Steindl et al. (2021) | Self-Report | 621 adults | M=43.55, SD=14.68 | 81.3% females, 18.7% male | X | | | | X | X | X | X | X | X | | | | | 7 |
| Compassion Motivation and Action Scales (CMAS) for Self-Compassion | Steindl et al. (2021) | Self-Report | 622 adults | M=43.55, SD=14.69 | 81.3% females, 18.7% male | X | | | | X | X | X | X | X | X | | | | | 7 |
| Compassion Scale | Martins et al. (2013) | Self-Report | 310 adults | | Female (66.8%) Male (33.2%) | X | | | | X | | | | X | | | | | | 3 |
| Compassion Scale (CS) | Pommier et al. (2020) | Self-Report | 465 adults (Study 1), 510 adults (Study 2), 80 adults (Study 3), 1394 adults (Study 4), 172 adults (Study 5), 913 adults (Study 6) (N=3534) | | Study 1 - Female (65%) Male (35%)<br>Study 2 - Female (53%) Male (47%)<br>Study 3 - Female (46%) Male (54%)<br>Study 4 - Female (65%) Male (35%)<br>Study 5 - Female (72%) Male (28%)<br>Study 6 - Female (45%) Male (55%) | X | X | | X | X | X | X | | X | X | X | X | | | 10 |
| Compassionate Care Assessment Tool (CCAT)© | Burnell & Agan (2013) | Self-Report | 250 medical/surgical inpatients | | Female (47%) Male (53%) | X | | | X | X | | X | | X | | | | | | 5 |
| Compassionate Engagement and Action Scales (CEAS) | Murfield et al. (2021) | Self-Report | 189 family carers of older adults | M=59.4, SD=11.5 | 87.7% female, 11.7% male | | | | | | | | | X | X | | | | | 2 |
| Compassionate Love Scale (CLS) | Sprecher & Fehr (2005) | Self-Report | 354 undergraduate students | | Female (65.3%) Male (34.7%) | X | | | | X | | X | | X | | | X | | | 5 |
| Compassionate Love Scale for Humanity - Short Form (CLS-H-SF) | | | | | | X | | | X | X | X | X | | X | X | | X | X | | 9 |

| Instrument | Reference | Study Type | Study Population | Age (range [R] and/or Mean [M] with Standard Deviation [SD]) | Gender | Internal Consistency | Test Re-Test Reliability | Interratter Reliability | Content Validity | Construct Validity | Discriminant/Divergent Validity | Convergent Validity | Predictive Validity | Factor Analysis/Principal Component Analysis | Confirmatory Factor Analysis | Structural Equation Modeling | Control/Correlation with Social Desirability | Other Advanced Statistics | Other Biased Responding or Lie Scale | Total Psychometric Assessments |
|---|---|---|---|---|---|---|---|---|---|---|---|---|---|---|---|---|---|---|---|---|
| | Williams et al. (2022) | Self-Report | 225 undergraduate paramedicine students | M = 21 | 65.8% female | X | | | | | | | | X | | | | | | 2 |
| | Chiesi et al. (2020) | Self-Report | 790 undergraduate students | | Female (66%) Male (34%) | X | | | X | X | | X | | | | | | X | | 5 |
| Complementary Measure of Psychotherapy Measure (COMPO) | Chui et al. (2021) | Self-Report | study 1: 10 graduate students/early career psychologists and 35 experienced therapists; study 2: 264 adults currently in outpatient psychotherapy and 571 adults; study 3: 31 clients seeking psychotherapy for depression and 9 therapists | study 2: development sample M= 36.1, SD 12.7, validation sample M= 32.9, SD 13.3; study 3: clients M=34.7, SD = 9.13 | study 1: 25 females; study 2: development 207 female, 52 male, 5 other; validation 442 female, 122 male, 6 other; study 3: 24 female | X | X | | X | X | X | X | X | X | X | X | | | | 10 |
| Comprehensive Object Relations Profile (CORP) | Burke et al. (1986) | Self-Report | 10 neurotic patients, 10 borderline patients, and 10 schizophrenic patients (N=30) | Ten subjects were tested in each of three diagnostic groups: neurotic, borderline, a schizophrenic. The neurotic patients were selected consecutively upon admission to the Emergency and Psychotherapy Program. The borderline and schizophrenic patients were selected consecutively upon admission to the Partial Hospitalization Program. | Female (57%) Male (43%) | | X | | X | | X | | X | | | | | | | 4 |
| Comrey Personality Scales (CPS) | | | | | | X | | | X | X | | | | X | X | | | X | | 6 |
| | Rushton & Irwing (2009) | Self-Report | | | Female (51%) Male (49%) | X | | | | | | | | X | X | | | | | 3 |
| | Caprara et al. (2001) | Self-Report | | | Female (63%) Male (37%) | X | | | X | X | | | | | X | | | X | | 5 |
| | Forbes et al. (1974) | Self-Report | 752 American and British undergraduate students and university employees | | Female (68.2%) Male (31.9%) | | | | | | | | | X | | | | | | 1 |
| Connection Index (CI12) | Puder et al. (2022) | Self-Report | 50 medical residents | N/A | 76 male, 53 female | X | X | | | X | | | | X | | | | | | 4 |
| Connor-Davidson Resilience Scale 10 (CD-RISC-10) | Heritage et al. (2021) | Self-Report | 708 nursing students | M=26.43 | 86% female | X | | | X | | | | | X | X | | | | | 4 |
| Consultation and Relational Empathy (CARE) Measure | | | | | | X | | X | X | X | | X | | X | X | | | X | | 8 |
| | Keulen et al. (2020) | Self-Report | 84 orthopedic patients | 20-88 | 44 female | X | | | | | | | | X | | | | | | 2 |
| | Bikker et al. (2017) | Self-Report | 943 adults attending consultations with sexual health nurses (n=20) | M = 28.8 | Female (68%) Male (32%) | X | | | | X | | X | | | | | | | | 3 |
| | Bikker et al. (2015) | Self-Report | 774 adult patients from GP practices | 16 and older | Female (57.8%) Male (42.2%) | X | | | | X | | X | | | | | | | | 3 |
| | Chen et al. (2015) | Self-Report | 158 medical students | | Female (39%) Male (61%) | X | | | X | X | | X | | | X | | | | | 5 |
| | Kersten et al. (2012) | Self-Report | 213 pain patients | M = 66.8, SD = 8.3 | Female (58%) Male (42%) | X | | | | | | | | X | | | | X | | 3 |
| | Mercer et al. (2008) | Self-Report | 1582 patients being treated by anaesthetists (n=31) | | Patients: Female (60.1%) Male (39.9%) | X | | X | | | | | | | | | | | | 2 |
| | Mercer & Murphy (2008) | Self-Report | 1015 patients rating outpatient medical consultants (n=25) in Scotland | | Female (64.8%) Male (35.2%) | X | | X | | X | | | | X | | | | | | 4 |
| | Mercer et al. (2004) | Self-Report | 20 Scottish medical outpatients (high economic deprivation) (Study 1), 13 Scottish medical outpatients (low economic deprivation) (Study 2), 10 Scottish medical outpatients (high economic deprivation) (Study 3) (N=43) | | Female (60.6%) Male (39.4%) | | | | X | X | | | | | | | | | | 2 |
| Consumer Orientation Questionnaire (SCOI) | Saunders & Munro (2000) | Self-Report | 136 undergraduate psychology students | | Female (72.8%) Male (27.2%) | X | X | | | X | X | X | | | | | X | | | 6 |
| Counselor Behavior Analysis (CBA) | | | | | | X | | X | X | X | X | X | X | | X | | | | | 8 |
| | Waugh et al. (2021) | Self-Report | 24 grdauate students and PhD-Level psychologists | M = 29.36, SD = 8.23 | not indicated | | | X | X | | | | | | | | | | | 2 |
| | Gabbard et al. (1986) | Self-Report | 44 practicing counselors | | N/a | X | | X | | X | X | X | X | | X | | | | | 7 |
| Curtin Vicarious Response Scales (CVRS) | Curtin & Hammond (2012) | Self-Report | 664 Irish dental clinicians | | Female (59.6%) Male (40.4%) | X | | | | X | | X | | X | | | | | | 4 |
| Decentering Scale for Sport (DSS) | Zhang et al. (2016) | Self-Report | 1255 athletic persons | | Study 1: Female 37.1%, Male 62.9% Study 2: Female 50.2%, Male 49.8% Study 3: Female 41.5%, Male 58.5% Study 4; Female 46.4%, Male 53.6% Study 5: Female 40.4%, Male 59.6% | X | | | X | X | X | X | | X | X | | | | | 7 |
| Defending Behaviors Scale (DBS) | Lambe & Craig (2020) | Self-Report | 572 early adolescents | | Female (50%) Male (49%) Other (1%) | X | X | | X | X | X | X | X | X | | X | | | | 9 |
| Dental Beliefs Survey - Revised (DBS-R) | Kvale et al. (2004) | Self-Report | 202 dental patients | | Female (77%) Male (23%) | X | | | | X | | | | X | X | | | | | 4 |
| Dentist-Patient Relationship Skills Assessment [Unnamed] | Orsini & Jerez (2014) | Self-Report | 444 undergraduate dental students | | N/a | X | | | X | X | | | | X | | | | | | 4 |
| Diabetes-Specific Self-Compassion Scale (SCS-Dp) | Tanenbaum et al. (2020) | Self-Report | 198 parents of youth with Type-1 Diabetes | M = 44, SD = 8.9 | Female (88%) Male (12%) | | | | | X | X | X | | | X | | | | | 4 |
| Driving While Impaired Therapeutic Educator Inventory (DTEI) | DeMuro et al. (2011) | Self-Report | 569 adults | M = 46.39 | Female (61.9%) Male (38.1%) | X | | | | X | | | | X | | | | | | 3 |
| DSM-5 Levels of Personality Functioning Questionnaire (DLOPFQ) | | | | | | X | | X | X | X | X | X | | | | | | | | 6 |
| | Waugh et al. (2021) | Self-Report | 29 grdauate students and PhD-Level psych | M = 29.36, SD = 8.23 | not indicated | | | X | X | | | | | | | | | | | 2 |
| | Huprich et al. (2018) | Self-Report | 140 outpatient psychiatry and internal medicine clinicians | | Female (73.5%) Male (26.5%) | X | | | | X | X | X | | | | | | | | 4 |
| | Nelson et al. (2018) | Self-Report | 140 patients who have been in treatment at outpatient psychiatry and internal medicine clinics | 18+ | Patients: Female (73.5%) Male (26.5%) | | | | | | | X | | | | | | | | 1 |
| Dymond's Test of Insight and Empathy | Lindgren & Robinson (1953) | Self-Report | 60 undergraduate psychology students | | N/a | | | | X | | | | | | | | | | | 1 |
| ECRC Altruism Scale | Bhatta et al. (2021) | Self-Report | 366 older adults free from significant physical and psychological health problems | M= 86.17, SD = 4.53 | 68% female | X | | | X | X | X | X | X | X | X | X | | | | 9 |
| Effects of Meditation (EOM) Scale | Skipper et al. (2015) | Self-Report | 254 individuals currently practicing meditation | R: 20-91 | N/a | X | | | | X | | | X | | X | | | | | 4 |
| Eight-Item Toronto Alexithymia Scale (TAS-8) | Williams & Gotham (2021) | Self-Report | 743 verbal autistic adults and 721 general population controls | autistic adults M=30.91, SD=7.02; control M=30.92, SD=13.01 | autistic sample: 36.5% male, 63.5% female; control 35.1% male, 64.9% female | | | | | X | X | X | | X | X | | | | | 5 |
| Emotional Accuracy Research Scale (EARS) | Geher et al. (2011) | Self-Report | 124 undergraduate students | | Female (58%) Male (42%) | X | | | | | X | X | X | | | | | | | 4 |
| Emotional Intelligence (EI) Instrument | Carrothers et al. (2000) | Self-Report | 147 medical school applicants | | Female (56%) Male (44%) | X | | | X | X | | X | | | | | | | | 4 |
| Emotional Intelligence Inventory (EII) | | | | | | X | X | X | X | | | X | | X | | | | | | 6 |
| | Tapia & Marsh (2006) | Self-Report | | | Female (64%) Male (36%) | X | | | | | | X | | | | | | | | 2 |
| | Singh (2004) | Self-Report | 263 managers | | Female (27.4%) Male (72.6%) | | | X | | | | | | | | | | | | 1 |
| | Tapia (2001) | Self-Report | 111 Mexican high school students | | Female (37%) Male (63%) | X | X | | X | | | | | X | | | | | | 4 |

| Instrument | Reference | Study Type | Study Population | Age (range [R] and/or Mean [M] with Standard Deviation [SD]) | Gender | Internal Consistency | Test Re-Test Reliability | Interratter Reliability | Content Validity | Construct Validity | Discriminant/Divergent Validity | Convergent Validity | Predictive Validity | Factor Analysis/Principal Component Analysis | Confirmatory Factor Analysis | Structural Equation Modeling | Control/Correlation with Social Desirability | Other Advanced Statistics | Other Biased Responding or Lie Scale | Total Psychometric Assessments |
|---|---|---|---|---|---|---|---|---|---|---|---|---|---|---|---|---|---|---|---|---|
| Emotional Intelligence Scale (EIS) | | | | | | X | | | | | | X | X | | X | | | | | 4 |
| | Tapia & Marsh (2006) | Self-Report | | | Female (64%) Male (36%) | X | | | | | | X | | | | | | | | 2 |
| | Hakanen (2004) | Self-Report | 153 undergraduate students | M = 25, SD = 4.4 | Female (54.2%) Male (45.8%) | | | | | | | | X | | X | | | | | 2 |
| Emotional Logic Development Profile (ELDP) | Zahra et al. (2016) | Self-Report | 118 adults | | Female (62.7%) Male (25.4%) Other (11.9%) | X | | | | X | | | | | | | | | | 2 |
| Emotional Response Questionnaire (ERQ) | Everson et al. (2018) | Self-Report | 460 nursing students | | N/a | X | | | | | | | | | X | X | | | | 3 |
| Emotional Skills and Competence Questionnaire (ESCQ) | Takšić et al. (2009) | Self-Report | 381 high school students | R: 14-19 | N/a | X | | | X | X | X | X | X | X | X | | | | | 8 |
| Empathetic Care Scale (ECS) | Lamberton et al. (2015) | Self-Report | 28 healthcare paraprofessionals (Study 1), 138 healthcare paraprofessionals (Study 2), 125 healthcare paraprofessionals (Study 3), 16 healthcare paraprofessionals (Study 4), 18 healthcare paraprofessionals (Study 5) (N=325) | Study 2: M = 46.3 | Female (92.8%) Male (7.2%) Female (88.6%) Male (11.4%) 3rd, 4th, 5th study sex information N/A | | X | | | X | X | X | X | X | X | | X | X | | 9 |
| Empathic Communication Coding System (ECCS) | Bylund & Makoul (2002) | Self-Report | 100 patient encounters with general internists (n=20) rated by researchers | | Female (20%) Male (80%) | | | | X | | | | | | | | | X | | 2 |
| Empathic Experience Scale (EES) | | | | | | X | | | X | X | X | X | | X | X | X | X | | | 9 |
| | Suwinyattichaiporn et al. (2021) | Self-Report | study 1: 151 undergraduate students; study 2: 535 undergraduate students; study 3: 659 undergraduate participants | study 2: M=21.22; study 3: M=19.54 | study 1: 69 men, 81 women; study 2: 320 female, 215 male; study 3: 338 men, 319 women) | X | | | X | X | X | X | | X | X | X | | | | 8 |
| | Innamorati et al. (2019) | Self-Report | 921 Italian adults (Study 1), 504 Italian adults (Study 2) (N=1425) | 18+ | Female (53.8%) Male (46.2%) | X | | | | | X | X | | X | X | X | X | | | 7 |
| Empathic Expressions Scale (EES) | | Self-Report | | | | | | | | | | | | | | | | | | 0 |
| Empathic Tendency Scale (ETS) | Koçak & Onen (2013) | Self-Report | 720 student teachers | | Female (71.3%) Male (30.1%) | X | X | | | | | | | X | X | | | | | 4 |
| Empathy Accuracy (EA) Task | Vilas et al. (2021) | Self-Report | study 1: 16 targets, 59 perceivers; study 2: 71 participants | study 1: M=19.02, SD=0.61; study 2: M=15.26, SD=1.28 | study 1: (targets: 7 males, 9 females) (perceivers: 50 females, 9 males); study 2: 37 males, 34 females | | | | | X | | X | | | | | | | | 2 |
| Empathy Assessment Index (EAI) | | | | | | X | X | | X | | | X | | X | X | | | | | 6 |
| | Gerdes et al. (2011) | Self-Report | 312 undergraduate and graduate students | | Female (83%) Male (17%) | X | X | | X | | | X | | X | | | | | | 5 |
| | Lietz et al. (2011) | Self-Report | 773 adults | | Female (74.4%) Male (25.6%) | X | X | | | | | X | | | X | | | | | 4 |
| Empathy Assessment Scale (EAS) | Malakcioglu (2022) | Self-Report | 651 students | M=20.34, SD=2.67 | 53.6% female, 46.4% male | X | X | | X | X | | X | | X | X | | | | | 7 |
| Empathy Components Questionnaire (ECQ) | Batchelder et al. (2017) | Self-Report | 101 undergraduate students and university staff | | Female (65.34%) Male (34.66%) | | | | | | | X | | X | X | | | | | 3 |
| Empathy-Enhancing Virtual Evolving Environment (EEVEE) | Jackson et al. (2015) | Self-Report | 19 adults | | N/a | | | | | | | | | | | | | X | | 1 |
| Empathy for Action Pain Picture Database (EPSS-Action_Picture) | Meng et al. (2023) | Self-Report | 70 adults | M=22.66, SD=2.17 | 36 females | X | | | X | | | | | | | | | | | 2 |
| Empathy for Action Pain Video Database (EPSS-Action_Video) | Meng et al. (2023) | Self-Report | 70 adults | M=23.23, SD=2.15 | 34 females | X | | | X | | | | | | | | | | | 2 |
| Empathy for Face Pain Picture Database (EPSS-Face) | Meng et al. (2023) | Self-Report | 70 paid volunteers | M=22.29, SD=2.53 | 35 women | X | | | X | | | | | | | | | | | 2 |
| Empathy for Limb Pain Picture Database (EPSS-Limb) | Meng et al. (2023) | Self-Report | 70 paid volunteers | M=25.34, SD=1.34 | 35 women | X | | | X | | | | | | | | | | | 2 |
| Empathy for Pain Scale (EPS) | Giummarra et al. (2015) | Self-Report | 161 pain patients, 245 adult controls (Study 1), 59 adults (Study 2) (N=465) | | Study 1: Female (56%) Male (44%) Study 2: Female (78%) Male (22%) | X | | | X | X | X | X | X | X | X | | | | | 8 |
| Empathy for Voice Pain Database (EPSS-Voice) | Meng et al. (2023) | Self-Report | 70 adults | M=22.89, SD=2.32 | 35 women | X | | | X | | | | | | | | | | | 2 |
| Empathy Gradient Questionnaire (EGQ) | Hollar (2017) | Self-Report | 161 undergraduate students and educational professionals | R: 18-60+ | Female (48.4%) Male (51.6%) | X | | | | | | | | X | X | | | | | 3 |
| Empathy Index (EI) | | | | | | X | | | | | | X | | X | X | | | X | | 5 |
| | Murphy et al. (2018) | Self-Report | 389 adults | | Female (52.5%)Male (47.5%) | | | | | | | | | | X | | | X | | 2 |
| | Jordan et al.(2016) | Self-Report | 95 adults recruited through MTurk | M = 36.52 | Female (51.6%) Male (48.4%) | X | | | | | | X | | X | | | | | | 3 |
| Empathy Measure for Parents Analog Task - Emotion Audio (EMPAT-EA) | Gonzalez & Rodriguez (2021) | Self-Report | study 1: 142 childless undergraduate students; study 2: 212 parents | study 1: M=18.77, SD=1.56; study 2: mothers M=30.44, SD=5.8, fathers M=32.65, SD=6.75 | study 1: 59% female; study 2: 119 mothers, 93 fathers | X | | | X | X | | X | | X | | | | | | 5 |
| Empathy Measure for Parents Analog Task - Emotion Scripts (EMPAT-ES) | Gonzalez & Rodriguez (2021) | Self-Report | study 1: 142 childless undergraduate students; study 2: 212 parents | study 1: M=18.77, SD=1.56; study 2: mothers M=30.44, SD=5.8, fathers M=32.65, SD=6.75 | study 1: 59% female; study 2: 119 mothers, 93 fathers | X | | | X | X | | X | | X | | | | | | 5 |
| Empathy Questionnaire for Children and Adolescents (EmQue-CA) | Overgaauw et al. (2017) | Self-Report | 1250 children/adolescents | 10 or older | Female (50%) Male (50%) | X | | | X | X | | X | | X | X | | | | | 6 |
| Empathy Quotient (EQ) | | | | | | X | X | | | X | X | X | X | X | X | | X | X | | 10 |
| | Jones et al. (2022) | Self-Report | 215 adult individuals without concomitant intellectula disability | M=31.18, SD=10.45 | 118 male | | | | | X | X | | X | | | | | | | 3 |
| | Peretti et al. (2019) | Self-Report | | | | | | | | | X | X | | X | | | | | | 3 |
| | Wright & Skagerberg (2012) | Self-Report | 5186 adults recruited through Qualtrics | online Qualtrics population | Female (59%) Male (41%) | X | | | | | X | | | | | | | | | 2 |
| | Allison et al. (2011) | Self-Report | 658 individuals with an autism spectrum diagnosis, 1375 family members of individuals with autism spectrum diagnosis, and 3344 typical adult controls (N=5377) | | Female (60.7%) Male (39.3%) | X | | | | | | | | | X | | | X | | 3 |
| | Nettle (2007) | Self-Report | 277 adults (Study 1), 195 adults (Study 2), 76 non-heterosexual adults (Study 3) (N=548) | people online, international | Study 1: Female (62.5%) Male (37.5%), Study 2: Female (67.7%) Male (32.3%). Study 3. Female (29%) Male (71%) | | | | | | | | | X | | | | | | 1 |
| | Lawrence et al. (2004) | Self-Report | 52 mental health professionals and staff (Study 1), 172 adults (Study 2) (N=224) | Study 1: M = 32.5 | Study 1: Female (52.8%) Male (47.2%) Study 2: Female (61.4%) Male (38.6%) Test group: Female (48.4%) Male (51.6%) | | X | | | X | | X | | X | | | X | | | 5 |
| Empathy Quotient - Child (EQ-C) | Auyeung et al. (2009) | Self-Report | 1256 parents of typically developing children | parents of typically developing children | N/a | X | X | | | | | | | | | | | | | 2 |
| Empathy Quotient - Short (EQ-Short) | Wakabayashi et al. (2006) | Self-Report | 1761 English undergraduate students | R: 18.2–26.3, M = 21.0, SD = 2.58 | Female (59%) Male (41%) | X | | | X | | | X | | X | | | | | | 4 |
| Empathy Scale for Social Workers (ESSW) | King & Holosko (2012) | Self-Report | 271 social workers | | N/a | X | | | X | X | X | X | | X | | | | | | 6 |
| Empathy Selection Task | | | | | | X | X | | | X | | X | X | | | | | X | | 6 |
| | Ferguson & Inzlicht (2022) | Self-Report | sample 1: 47 general adult population; sample 2: 42 undergraduate students | study 1: M age = 35.9 years study 2: M age = 19 years | study 1: 18 female, 29 male study 2: 34 female, 8 male | X | X | | | | | | | | | | | | | 2 |
| | Cameron et al. (2019) | Self-Report | 56 adults recruited through MTurk (Study 1), 47 adults recruited through MTurk (Study 2), 196 adults recruited through MTurk (Study 3) (N=299) | | Female (56.5%) Male (43.5%) | | | | | X | | X | X | | | | | X | | 4 |

| Instrument | Reference | Study Type | Study Population | Age (range [R] and/or Mean [M] with Standard Deviation [SD]) | Gender | Internal Consistency | Test Re-Test Reliability | Interratter Reliability | Content Validity | Construct Validity | Discriminant/Divergent Validity | Convergent Validity | Predictive Validity | Factor Analysis/Principal Component Analysis | Confirmatory Factor Analysis | Structural Equation Modeling | Control/Correlation with Social Desirability | Other Advanced Statistics | Other Biased Responding or Lie Scale | Total Psychometric Assessments |
|---|---|---|---|---|---|---|---|---|---|---|---|---|---|---|---|---|---|---|---|---|
| Empathy Test | Layton & Wykle (1990) | Self-Report | | | Female (88%) Male (12%) | | | | | X | | | | | | | | | | 1 |
| Empathy Towards People with Intellectual Disabilities Questionnaire (EMP-ID) | Collins et al. (2017) | Self-Report | 194 employed carers | | Female (74%) Male (26%) | X | X | | X | X | | X | | | | | X | | | 6 |
| English Youth Self-Report Version of the ICU | Hitti et al. (2021) | Self-Report | 265 middle schoolers | 11-15 | female (52%) | X | | | | X | X | X | | | | | | X | | 5 |
| Equanimity Barriers Scale (EBS) | | | | | | X | | X | X | X | X | X | | X | X | | | | | 8 |
| | Weber & Lowe (2021) | Self-Report | study 1 = 453 students and staff; study 2 = 108 undergraduate students; study 3 = 302 undergraduate students | study 1: M=30.55, SD=11.26; study 2: M=28.79, SD=10.58; study 3: M=30.24, SD=9. 52 | study 1: 20.2% male, 79.1% female; study 2: 32.4% male, 64.8% female; study 3: 21.2% male, 77.8% female | X | | | X | X | X | X | | X | X | | | | | 7 |
| | Weber & Lowe (2018) | Self-Report | 453 undergraduate health and psychology students and online social media respondents (Study 1), 108 undergraduate health students (Study 2), 302 undergraduate health and psychology students (Study 3) (N=863) | | S1: Female (79.1%) Male (20.2%) Unspecified (0.7%) S2: Female (64.8%) Male (32.4%), Unspecified (2.8%) S3: Female (77.8%) Male (21.2%) Gender fluid (1%) | X | | | | X | X | X | | X | X | | | | | 6 |
| Ethical Wills Assessment | Cohen-Mansfield et al. (2009) | Self-Report | 15 nursing home residents, 11 student volunteers at nursing homes (N=26) | | Female (59%) Male (41%) | | | | X | | | | | | | | | | | 1 |
| Ethnic Minority Patient-3 (EMP-3) | De Maesschalck et al. (2010) | Self-Report | 112 family physicians | | Female (36.6%) Male (60.7%) Did not respond (2.7%) | X | | | X | X | | | | X | | | | | | 4 |
| Evaluating Goelman's Five Dimensions of Emotional Intelligence [Unnamed] | Branscum et al. (2013) | Self-Report | 189 undergraduate students | | Female (49.7%) Male (50.3%) | X | | | X | X | X | X | X | | X | | | | | 7 |
| Everyday Multicultural Competencies/Revised Scale of Ethnocultural Empathy(EMC/RSEE) | Mallinckrodt et al. (2014) | Self-Report | 28 key campus administrators responsible for diversity programming, residence hall peer counselors, and counseling psychology doctoral students involved in undergraduate teaching (Development Study), 1278 undergraduate psychology students (Validation Study) (N=1306) | | N/a | X | | | | | X | X | | | X | | | | | 4 |
| Evidence-Based Practice Profile (EBP2) Questionnaire | McEvoy et al. (2010) | Self-Report | 526 students and academics (Development Sample), 105 allied health students and professionals (Validation Sample) (N=631) | | Female (66.7%) Male (33.3%) | X | X | | | | X | X | | X | | | | X | | 6 |
| Executive Function Index (EFI) | Spinella (2005) | Self-Report | 188 adults | | Female (57%) Male (43%) | X | | | | X | X | X | | X | | | | | | 5 |
| Experience Sampling Method (ESM) | Runyan et al. (2019) | Self-Report | 200 adults | | Female (65.7%) Male (34.3%) | X | | | | | | X | X | | | | | | | 3 |
| Eysenck Personality Inventory (EPI) | Kentle (1995) | Self-Report | 300 undergraduate students | M = 21.14, SD = 2.8 | Femal (50%) Male (50%) | | | | | | | X | | | | | | | | 1 |
| Eysenck Personality Questionnaire - Revised (EPQ-R) | Corulla (1987) | Self-Report | 307 undergraduate students | | Female (70%) Male (30%) | X | | | X | X | | X | | | | | | | X | 5 |
| Facets of Emotional Experiences in Everyday Life Scale (FEEELS) | Chung et al. (2022) | Self-Report | study 1: 55 participants; study 2: 107 participants; study 3: 229 participants | study 1: M=34.909, SD=9.536; study 2 M=36. 810, SD =10.609; study 3: M=36.486, SD: 10.353 | study 1: 51% males, 49% females; study 2: 51% males, 49% females, 40% males, 58% females, .5% genderqueer/gender fluid, .5% trans x man, . 5% trans x woman, .5% "rather not say" | X | | | X | X | X | X | | X | X | X | | | | 8 |
| Facilitative Interpersonal Skills for Child Therapy (FIS-C) | Bate & Tsakas (2022) | Self-Report | 10 mental health clinicians, either licensed or in training | M = 29.5, SD = 8.07 | 9 female, 1 male | X | | X | X | X | | X | | | | | | | | 5 |
| Family Nurse Caring Belief Scale (FNCBS) | Magri (2018) | Self-Report | 213 nurses | | Female (98.6%) Male (1.4%) | X | | | | | X | X | | X | X | | | | | 5 |
| Family Response Questionnaire (FRQ) | Cordingley et al. (2001) | Self-Report | 78 close relatives, partners, or friends currently living with a person with CFS | | Female (59%) Male (41%) | X | X | | X | X | | X | | | | | | X | | 6 |
| Fat Attitudes Assessment Toolkit (FAAT) | Cain et al. (2022) | Self-Report | study 1: 751 American adults (at least 18 years old). study 2: 390 American adults | study 1: median age 38.2; study two average age 31.47 | study 1: 369 women, 378 men, 4 other. study 2: 195 men and women | X | X | | X | X | X | X | | X | X | | X | | | 9 |
| Fears of Compassion for Self Scale | Geller et al. (2019) | Self-Report | 251 Canadian patients referred for eating disorder treatment (Clinical Sample), 314 Canadian female undergraduate psychology students (Student Sample) (N=565) | | Female (98.5%) Male (1%) Other (0.5%) | | | | | | | | | X | | X | | | | 2 |
| Fears of Compassion in Sport Scale (FCSS) | Zhang & McEwan (2023) | Self-Report | 792 athletes | study 1 (sample 1: M=27. 75, SD=8.94; sample 2: M=24.28, SD=7.79), study 2 M=22.87, SD=2. 33 | study 1 (sample 1: 167 male, sample 2: 131 male), study 2: 78 male | X | X | | X | X | X | X | X | X | X | | | | | 9 |
| Fears of Compassion Scales | Gilbert et al. (2011) | Self-Report | 222 undergraduate students, 59 therapists attending a three-day Compassion-Focused Therapy workshop (N=281) | | Female (77%) Male (23%) | X | | | X | X | X | X | | X | | | | | | 6 |
| Figley's Compassion Fatigue Scale - Revised | Adams et al. (2006) | Self-Report | 236 social workers | | Female (80%) Male (20%) | X | | | | | | X | | X | | | | | | 3 |
| Five Facet Mindfulness Questionnaire (FFMQ) | | | | | | X | | | | X | | X | | X | X | | | | | 5 |
| Five-Factor Narcissism Inventory - Short Form (FFNI-SF) | Sherman et al. (2015) | Self-Report | 166 undergraduate psychology students (Sample 1), 288 undergraduate students (Sample 2), 287 adults recruited through MTurk (Sample 3), 98 adults currently receiving psychological/psychiatric treatment (Sample 4) (N=839) | | Female (64.6%) Male (35.4%) | | | | | | | | | X | | | | X | | 2 |
| Forms of Responding to Self-Critical Thoughts Scale (FoReST) | White et al. (2020) | Self-Report | 254 adults (Study 1), 110 adults (Study 2) (N=364) | Study 1: M = 31 Study 2: M = 21.6 | Female (69.5%) Male (29.7%) Other (.8%) | X | | | X | | | X | | X | X | | | X | | 6 |
| Four-Dimensional Connectedness Scale (4DCS) | Huynh et al. (2012) | Self-Report | 463 Australian volunteer rural ambulance officers | | Female (51%) Male (49%) | X | | | | | X | | X | X | X | | | | | 5 |
| Four-Item Mentalising Index | Clutterbuck et al. (2021) | Self-Report | study 1: 669 English-speaking participants; study 2a: 1999 English-speaking adults; study 2b: 116 students; study 3a: 500 English-speaking participants; study 3b: 102 adults with a confirmed clinical diagnosis of autism & 183 non-autistic individuals | study 1b: M=36.39, SD=12.14; study 2a: M=35.77, SD=12.82; study 2b: range 18=-20; study 3a: M=37.29, SD=11.74; study 3b: M=41.74, SD=12.15 | study 1b: 342 females; study 2a: 1286 females; study 2b: 93 females; study 3a: 254 females; study 3b: 62 females) | X | X | | X | X | X | X | X | X | X | X | | | | 10 |
| General Anesthesia Satisfaction Questionnaire | Hawkins et al. (2014) | Self-Report | 125 military persons | | Female (49.6%) Male (49.6%) Others (0.8%) | | | | | | | | | X | | | | | | 1 |
| Genuine Happiness Scale (GHS) | Rudaz et al. (2023) | Self-Report | 679 undergraduate students | M=20.23, SD=2.33 | 90% female, 9.7% male | X | X | | X | X | X | X | X | X | X | X | | | | 10 |
| Global Rating Form for ACGME | Silber et al. (2004) | Self-Report | 1295 medical residents | | N/a | X | | | | X | | | | X | | | | | | 3 |
| Grid Measure of Empathy [Unnamed] | Smail (1972) | Self-Report | 6 patients in group therapy, 1 therapist (N=7) | R: 22-37, M = 29 | Female (85.7%) Male (14.3%) | | | | | X | | X | | | | | | | | 2 |
| Groningen Reflection Ability Scale (GRAS) | Aukes et al. (2007) | Self-Report | 538 medical students and medical teachers (Study 1), 1029 medical students and medical teachers (Study 2) (N=1567) | | N/a | X | | | X | | | | | X | | | | | | 3 |
| Group-Analysis-Empathy 1 (GA-Em1) | Pavlović & Vlastelica (2008) | Self-Report | | | Female (59%)Male (41%) | X | | | X | X | | | | X | | | X | | | 5 |
| Group-Analysis-Empathy 2 (GA-Em2) | Pavlović & Vlastelica (2008) | Self-Report | | | Female (59%)Male (41%) | X | | | X | X | | | | X | | | X | | | 5 |
| Group Questionnaire (GQ) | Thayer (2014) | Self-Report | 290 patients in group psychological treatment | | Female (49%) Male (51%) | X | | | X | X | | X | | | X | | | | | 5 |
| Hate Crime Beliefs Scale (HCBS) | | Self-Report | | | | X | | | | | | X | | X | X | | | | | 4 |
| | Kehn et al. (2022) | Self-Report | 463 general adult popultion | age = 25.55 average | 340 female | X | | | | | | X | | X | X | | | | | 4 |

| Instrument | Reference | Study Type | Study Population | Age (range [R] and/or Mean [M] with Standard Deviation [SD]) | Gender | Internal Consistency | Test Re-Test Reliability | Interratter Reliability | Content Validity | Construct Validity | Discriminant/Divergent Validity | Convergent Validity | Predictive Validity | Factor Analysis/Principal Component [illegible] | Confirmatory Factor Analysis | Structural Equation Modeling | Control/Correlation with Social Desirability | Other Advanced Statistics | Other Biased Responding or Lie Scale | Total Psychometric Assessments |
|---|---|---|---|---|---|---|---|---|---|---|---|---|---|---|---|---|---|---|---|---|
| | Bacon et al. (2020) | Self-Report | 438 undergraduate students | | Female (82%) Male (18%) | X | | | | | | | | X | X | | | | | 3 |
| Hate Crime Beliefs Scale - UK (HCBS-UK) | Bacon et al. (2021) | Self-Report | study 1: 438 undergraduate students study 2: 134 general public | study 1: 20.79 mean age, SD = 4.14 study 2: 36.44 mean age, SD = 11.32 | study 1: 186 female; 22 male; 3 non binary; 1 transgender study 2: 102 female; 29 male; 3 gender fluid | | | | | X | | | | | | | | X | | 2 |
| Health Care Providers Confidence in FGM/C Care | Marea et al. (2021) | Self-Report | 534 health care providers | not indicated | 82.5% female, 12.7% male, 4.8% other/missing | X | | | X | X | X | X | | X | | | | | | 6 |
| Health Communication Assessment Tool© (HCAT©) | Campbell et al. (2013) | Self-Report | 350 nursing students | | Female (90%) Male (10%) | X | | X | | | | | | X | | | | | | 3 |
| Hogan Empathy Scale (HES) | | | | | | X | X | X | | X | X | X | X | X | | | | | | 8 |
| | Stosic et al. (2022) | Self-Report | 183 undergraduates | M=22.1, SD = 4.79 | 69 male, 113 female | | | | | X | X | X | X | | | | | | | 4 |
| | Froman & Peloquin (2001) | Self-Report | 320 therapy students | | Female (59%) Male (13%) Other (28%) | X | X | | | X | X | X | | X | | | | | | 6 |
| | Hornblow et al. (1977) | Self-Report | 30 Australian medical students | | Female (33.3%) Male (66.7%) | | | X | | | | X | | | | | | | | 2 |
| | Wallston & Weitz (1975) | Self-Report | 56 undergraduate counseling students | mean = 28.4 | Female (64.3%) Male (35.7%) | | X | | | X | | | | | | | | | | 2 |
| Hospice Volunteer as Patient Advocate (HVPA) Scale | Savery & Egbert (2010) | Self-Report | 136 hospice volunteers | | Female (80.8%) Male (18.5%) Other (0.7%) | | | | | | | | X | | | | | | | 1 |
| Hospital Nurse Interpersonal Empathy Questionnaire (HNEIQ) | Montazeri et al. (2020) | Self-Report | 250 nurses | | Female (80.8%) Male (19.2%) | X | X | X | X | X | | | | X | | | | | | 6 |
| Huddersfield Service User Pedagogy Scale | Tobbell et al. (2018) | Self-Report | 365 undergraduate health/social care students | | Female (81.9%) Male (18.1%) | X | | | | X | | | | | X | | | | | 3 |
| Human Empathetic Listening Test (HELT) | Gray et al. (1976) | Self-Report | 90 undergraduate students, graduate students, and telephone crisis workers | | N/a | X | | | X | X | | | | | | | | | | 3 |
| Human Factors Skills for Healthcare Instrument - Auxiliary Version (HuFSHI-A) | Lavelle et al. (2019) | Self-Report | 188 non-clinical professionals working in healthcare contexts | | Female (59%) Male (41%) | X | | | X | | | | | X | | | | X | | 4 |
| Humanised Care Assessment Tool (HCAT) | Galvin et al. (2018) | Self-Report | 10 nursing students | | Female (80%) Male (20%) | | | | X | | | | | | | | | | | 1 |
| Humanistic Relationship Importance Scale (HRIS) | Boscart et al. (2016) | Self-Report | 249 patients residing in five nursing facilities | patients residing in five nursing facilities | Female (57.5%) Male (42.5%) | X | X | | X | X | | | | X | | | | X | | 6 |
| Illness Invalidation Inventory (3*I) | Kool et al. (2014) | Self-Report | 6057 patients with rheumatic disease | | Female (88%) Male (12%) | | | | | | | | | | X | | | | | 1 |
| Impact of Cancer Scale - Version 2 (IOCv2) | Crespi et al. (2008) | Self-Report | 1188 early-stage breast cancer survivors | | Female (100%) Male (0%) | X | | | | X | X | X | | X | | | | X | | 6 |
| Implicit Relational Assessment Procedure (IRAP) | | | | | | X | | | | | | X | X | | | | | X | | 4 |
| | Bast (2015) | Self-Report | 81 Irish undergraduate and postgraduate students | | N/a | | | | | | | X | | | | | | | | 1 |
| | Bast et al. (2015) | Self-Report | 26 undergraduate students | | N/a | X | | | | | | | X | | | | | X | | 3 |
| Impulsiveness-Venturesomeness-Empathy Questionnaire [Unnamed] | Eysenck & Eysenck (1978) | Self-Report | 1189 adults | M = 24.63, SD = 9.00 | Female (66.2%) Male (33.8%) | X | | | | | | X | | | | X | | | X | 4 |
| Index of Empathetic Concern | Matthews et al. (1981) | Self-Report | 88 undergraduate students (Validation Sample), 230 twin pairs (Twin Sample, MZ and DZ) (N=548) | | Female (0%) Male (100%) | | | | | X | | X | | X | | | | | | 3 |
| Index of Responding | Cummings & Murray (1990) | Self-Report | 70 elementary and secondary teachers | | Female (50%) Male (50%) | X | | | | X | | | | X | | | | | | 3 |
| (Inner) Strength-Based Inventory (SBI) | Wongpakaran et al. (2020) | Self-Report | 556 medical students | | Female (51.2%) Male (48.8%) | | | X | | | | | | X | | | | | | 2 |
| Insanity Defense Attitude - Revised (IDA-R) Scale | Adjorlolo et al. (2017) | Self-Report | 253 undergraduate students | | Female (56%) Male (44%) | X | | | | X | X | | X | X | X | | | X | | 7 |
| Integrity Scale | Hart et al. (2022) | Self-Report | 309 students | M=19.01, SD=1.5 | 81.2% female | X | | | | X | | X | | X | X | | | | | 5 |
| Interaction with Disabled Persons Scale (IDP) | | | | | | X | | | | X | X | X | | X | X | | X | | | 7 |
| | Thomas et al. (2003) | Self-Report | 358 undergraduate students | R: 18-55, M = 22.4, SD = 6.2 | Female (68.2%) Male (31.8%) | X | | | | X | X | | | X | X | | X | | | 6 |
| | MacLean & Gannon (1995) | Self-Report | 343 undergraduate students | 17-above | Female (65%) Male (35%) | X | | | | | | X | | X | | | | | | 3 |
| Interconnectedness Scale | Yu et al. (2020) | Self-Report | 325 undergraduate and postgraduate students (Study 1, PCA), 581 undergraduate and postgraduate students (Study 1, CFA), 194 undergraduate and postgraduate students (Study 2), 170 undergraduate and postgraduate students (Study 3) (N=1270) | | study 1: PCA: Female 57.8% male 42.2%; CFA: Female 66.4% male 33.6% study 2: Female 56.2%, Male 43.8% study 3: female 63.1%, male 36.9% | | | | | | | X | | X | X | | | | | 3 |
| Intercultural Effectiveness Scale (IES) | Cui & Van Den Berg (1991) | Self-Report | 84 American business people working in China | | N/a | | | | | X | | | | | X | | | | | 2 |
| International Personality Item Pool (IPIP) | Miller (2009) | Self-Report | 382 adults (Sample 1), 455 adults (Sample 2) (N=837) | | Female (51.6%) Male (48.4%) | | | | | | | | | | X | X | | X | | 3 |
| Interpersonal and Social Empathy Index (ISEI) | Segal et al. (2013) | Self-Report | 450 undergraduate students | | Female (66%) Male (34%) | X | | | | X | | | | X | X | | | | | 4 |
| Interpersonal Communication Assessment Scale (ICAS) | Klakovich & Dela Cruz (2006) | Self-Report | 578 undergraduate and graduate nursing students | | Female (92%) Male (8%) | X | | | | X | | | | X | | | | | | 3 |
| Interpersonal Communication Competence Scale (ICCS) | | | | | | X | | | | X | | X | | | X | | | X | | 5 |
| | Hald et al. (2015) | Self-Report | 17 adults with ABI | | Female (35%) Male (65%) | X | | | | X | | | | | X | | | X | | 4 |
| | Rubin & Martin (1994) | Self-Report | 477 adults | | Female (52%) Male (48%) | X | | | | | | X | | | | | | | | 2 |
| Interpersonal Guilt Rating Scale –15 Self-Report (IGRS-15s) | Faccini et al. (2020) | Self-Report | 448 adults | 18 or older | Female (60.7%) Male (39.3%) | X | | | | X | | X | | | X | X | X | | | 6 |
| Interpersonal Reactivity Index (IRI) | | | | | | X | X | | X | X | X | X | X | X | X | X | X | X | | 12 |
| | Bonfils et al. (2022) | Self-Report | 218 undergraduate students who endorsed a high level of schizotypal traits | M = 19.9 (SD = 3.2) | 20.6% male, 79.4% female | X | | | | | | X | | | X | | | | | 3 |
| | Stosic et al. (2022) | Self-Report | 182 undergraduates | M=22.1, SD = 4.79 | 69 male, 113 female | | | | | X | X | X | X | | | | | | | 4 |
| | Murphy et al. (2020) | Self-Report | | | Female (53%) Male (47%) | X | | | X | X | | X | | | X | X | | | | 6 |
| | Rajput et al. (2020) | Self-Report | 100 dental students | M=24.45, SD=1.95 | 61 female, 39 male | X | X | | | X | | | | X | X | | | | | 5 |
| | Leinweber et al. (2019) | Self-Report | 705 midwives | | Female (100%) Male (0%) | X | | | X | X | | X | | X | | | | | | 5 |
| | Peretti et al. (2019) | Self-Report | | | | | | | | | X | X | | X | | | | | | 3 |
| | Chrysikou & Thompson (2016) | Self-Report | 417 adults recruited through MTurk | | Female (56.8%) Male (43.2%) | | | | | | | | | | X | | | X | | 2 |
| | Saleem et al. (2015) | Self-Report | 162 adults (Study 1), 177 adults (Study 2), 146 adults (Study 3) (N=485) | | Study 1 - Female (54%) Male (46%) Study 2 - Female (41%) Male (59%) Study 3 - Female (49%) Male (51%) | | | | | | X | X | | | | | X | | | 3 |
| | Alterman et al. (2003) | Self-Report | 241 opioid dependent patients undergoing methadone maintenance | | Female (18.3%) Male (81.7%) | X | | | | X | X | X | | X | X | | | | | 6 |
| | Yarnold et al. (1996) | Self-Report | 114 physicians, 95 undergraduate psychology students (N=209) | | N/a | | X | | | X | X | | | | X | | | | | 4 |
| | Thornton & Thornton (1995) | Self-Report | 206 undergraduate students | | Female (50.5%) Male (49.5%) | | | | | | | | | X | | | | | | 1 |
| | Gurtman (1992) | Self-Report | 279 undegraduate students | | Female (66%) Male (34%) | | | | X | X | | | | | | | | X | | 3 |

| Instrument | Reference | Study Type | Study Population | Age (range [R] and/or Mean [M] with Standard Deviation [SD]) | Gender | Internal Consistency | Test Re-Test Reliability | Interratter Reliability | Content Validity | Construct Validity | Discriminant/Divergent Validity | Convergent Validity | Predictive Validity | Factor Analysis/Principal Component Analysis | Confirmatory Factor Analysis | Structural Equation Modeling | Control/Correlation with Social Desirability | Other Advanced Statistics | Other Biased Responding or Lie Scale | Total Psychometric Assessments |
|---|---|---|---|---|---|---|---|---|---|---|---|---|---|---|---|---|---|---|---|---|
| Interpersonal Reactivity Index - Brief Form (B–IRI) | Ingoglia et al. (2016) | Self-Report | 1104 Italian adolescents | R: 14-30 | Female (62%) Male (38%) | X | | | | X | | | | X | X | | | | | 4 |
| Interpersonal Regulation Questionnaire (IRQ) | Williams et al. (2018) | Self-Report | 285 undergraduate students (Study 1), 347 undergraduate students (Study 2), 400 undergraduate students (Study 3), 787 undergraduate students (Study 4), 193 undergraduate students (Study 5) (N=2012) | | S1: Female (41.4%) Male (58.6%) S2: Female (47.8%) Male (52.2%) S3: Female (41%) Male (59%) s4: Female (46.4%) Male (53.6%) S5: Female 52.3%) Male (47.7%) | | X | | | X | X | X | X | X | X | | X | | | 8 |
| Interview Satisfaction Questionnaire (ISQ) | Grayson-Sneed et al. (2016) | Self-Report | 105 medical residents | | Female (40%) Male (60%) | X | | | | X | | X | | | X | | | | | 4 |
| Inventory of Beliefs about Wife Beating (IBWB) | Saunders et al. (1987) | Self-Report | 675 students (Study 1), 1091 adults (undergraduate students, nurses, physicians, men entering treatment for abusing women, women advocates for abused women, and individuals from a separate study on attitudes about woman abuse) (Study 2), 719 adults (undergraduate students, women advocates for abused women, and men entering treatment for abusing women) (Study 3) (N=2485) | | N/a | X | | | | X | | X | | X | | | X | | | 5 |
| Inventory of Cross-Cultural Sensitivity (ICCS) | Loo & Shiomi (1999) | Self-Report | 273 Japanese undergraduate teaching students (Sample 1), 211 Canadian undergraduate management students (Sample 2) (N=484) | | Japanese group: Female (69%) Male (31%) Canadian group: Female (42%) Male (58%) | X | | | | | | | | X | X | | | | | 3 |
| Inventory of Motivations for Palliative Care Volunteerism (IMPCV) | Claxton-Oldfield et al. (2011) | Self-Report | 141 undergraduate students (Study 1), 141 hospital palliative care volunteers (Study 2) (N=282) | | Female (80.1%) Male (19.9%) | X | | X | | X | | X | | | X | | | | | 5 |
| Inventory of Personality Organization (IPO) | Waugh et al. (2021) | Self-Report | 27 grdauate students and PhD-Level psychologists | M = 29.36, SD = 8.23 | not indicated | | | X | X | | | | | | | | | | | 2 |
| Jefferson Scale of Attitudes toward Physician–Nurse Collaboration (JSAPNC) | Ward et al. (2008) | Self-Report | 333 undergraduate students | | Female (85%) Male (15%) | X | | | | X | | | | X | | | | | | 3 |
| Jefferson Scale of Empathy (JSE) | | | | | | X | | | | X | | | | X | X | X | | | X | 6 |
| | Hojat et al. (2018) | Self-Report | 6009 osteopathic medicine students | | Female (47%) Male (53%) | X | | | | X | | | | X | X | X | | | X | 6 |
| | Stansfield et al. (2016) | Self-Report | 4797 medical students | | Female (49.5%) Male (50.5%) | X | | | | X | | | | X | X | | | | | 4 |
| | Hojat & LaNoue (2014) | Self-Report | 2612 medical students | | Female (51%) Male (49%) | X | | | | X | | | | X | X | X | | | | 5 |
| Jefferson Scale of Empathy - Health Profession Students (JSE-HPS) | | | | | | X | | X | | X | | | | X | X | X | | | | 6 |
| | Shekhawat et al. (2017) | Self-Report | 150 undergraduate students, interns, and postgraduate students | | N/a | | | X | | X | | | | X | | | | | | 3 |
| | Williams et al. (2013a) | Self-Report | 330 undergraduate paramedic students | | Female (65.2%) Male (34.8%) | X | | | | X | | | | X | X | | | | | 4 |
| | Fjortoft et al. (2011) | Self-Report | 187 pharmacy students | | N/a | X | | | | X | | | | | | X | | | | 3 |
| Jefferson Scale of Empathy - Law Students (JSE-LS) | | | | | | X | | | | X | | | | X | | | | | | 3 |
| | Spivak et al. (2018) | Self-Report | 3072 undergraduate law students | M = 21.14, SD = 2.8 | Female (72.8%) Male (27.2%) | X | | | | X | | | | X | | | | | | 3 |
| | Williams et al. (2016a) | Self-Report | 275 undergraduate law students | | Females (66.9%) Males (33.1%) | X | | | | X | | | | X | | | | | | 3 |
| Jefferson Scale of Empathy - Medical Student Version (JSE-MS) | Hojat & Gonnella (2015) | Self-Report | 2637 medical students | | Female (51%) Male (49%) | X | | | X | | | | | | | | | | | 2 |
| Jefferson Scale of Patient's Perception of Physician Empathy (JSPPPE) | | | | | | X | | | | | X | X | | X | | | | | | 4 |
| | Keulen et al. (2020) | Self-Report | 84 orthopedic patients | 20-88 | 44 female | X | | | | | | | | X | | | | | | 2 |
| | Glaser et al. (2007) | Self-Report | | | Female (64%) Male (36%) | | | | | | X | X | | | | | | | | 2 |
| | Kane et al. (2007) | Self-Report | 225 internal medicine patients | | N/a | X | | | | | | | | X | | | | | | 2 |
| Jefferson Scale of Physician Empathy (JSPE) | | | | | | X | X | | X | X | X | X | | X | X | X | | X | | 10 |
| | Osim et al. (2019) | Self-Report | 120 Nigerian doctors | | Female (41.7%) Male (58.3%) | X | | | | X | | X | | X | | | | | | 4 |
| | Aggarwal et al. (2016) | Self-Report | 978 Indian dental students | | Female (68.4%) Male (31.6%) | X | | | | | | | | | X | | | | | 2 |
| | Spasenoska et al. (2016) | Self-Report | 193 Malaysian medical students | | N/a | X | | | | X | | | | X | | | | X | | 4 |
| | O'Connor et al. (2014) | Self-Report | 163 final-year medical students | | Female (53.4%) Male (46.6%) | | | X | X | | | | | | | | | | | 2 |
| | Tavakol et al. (2011) | Self-Report | 853 medical students in the UK | | Female (55%) Male (45%) | X | | | | | | | | X | X | X | | | | 4 |
| | Ward et al. (2009) | Self-Report | 333 undergraduate students | | Female (85%) Male (15%) | X | | | | X | | X | | X | | | | | | 4 |
| | Glaser (2007) | Self-Report | | | Female (64%) Male (36%) | | | | | | X | X | | | | | | | | 2 |
| | Sherman & Cramer (2005) | Self-Report | 130 dental students | | Female (34.6%) Male (65%) | X | | | | X | X | X | | X | | | | | | 5 |
| | Hojat et al. (2002a) | Self-Report | 704 physicians | | Female (26%) Male (74%) | | | | X | X | | | | | | | | | | 2 |
| | Hojat et al. (2002b) | Self-Report | 704 physicians | | N/a | X | X | | | X | | | | X | | | | | | 4 |
| | Hojat et al. (2001) | Self-Report | 193 physicians, medical residents, and medical students | | Female (40%) Male (60%) | X | | | | X | | X | | X | | | | | | 4 |
| Kiersma-Chen Empathy Scale (KCES) | | | | | | X | X | | | X | | X | | X | X | | | | | 6 |
| | Aronson et al. (2022) | Self-Report | 7712 student pharmacists, student nurses, physical therapy students, nurses, and physicians | NA | NA | X | X | | | X | | | | X | X | | | | | 5 |
| | Kiersma et al. (2013) | Self-Report | 216 pharmacy and nursing students | | Female (100%) Male (0%) | X | | | | X | | X | | | X | | | | | 4 |
| Kiersma-Chen Empathy Scale Revised Version (KCES-R) | Blakely et al. (2022) | Self-Report | 94 second-year student pharmacists | not indicated | 38% male | X | X | X | | X | | X | | X | | | | | | 6 |
| Kindness Scale | Youngs et al. (2021) | Self-Report | 1039 adults | M = 50, SD = 16.57 | 50% male, 50% female | X | | | X | X | X | X | | X | X | | | | | 7 |
| Kirk Adoption Questionnaire (KAQ) | Lo et al. (2021) | Self-Report | 190 adoptive mothers | wave 1: Mean child age = 7.8, wave 2: mean child age = 15.7 | 100% mothers | X | | X | | | X | | | X | X | | | | | 5 |
| Knee Osteoarthritis Expectations Questionnaire | Benhamou et al. (2013) | Self-Report | 10 experts (Development Study), 524 knee osteoarthritis patients (Validation Study) (N=534) | Phase 2: M = 68.2 | | X | X | | | X | X | X | | X | | X | | | | 7 |
| Leeds Satisfaction Questionnaire | Hill et al. (1992) | Self-Report | 70 patients with rheumatology arthritis | | N/a | X | X | | X | | | | | | | | | | | 3 |
| Level of Personality Functioning Scale (LPFS) | Morey (2017) | Self-Report | 306 adults | | Female (43%) Male (57%) | X | | | | X | | | | | | | | | | 2 |
| Level of Personality Functioning Scale - Brief Form 2.0 (LPFS-BF 2.0) | Waugh et al. (2021) | Self-Report | 23 grdauate students and PhD-Level psychologists | M = 29.36, SD = 8.23 | not indicated | | | X | X | | | | | | | | | | | 2 |
| Level of Personality Functioning Scale - Self Report (LPFS-SR) | | | | | | X | X | X | X | X | X | X | | X | | | | | | 8 |
| | Waugh et al. (2021) | Self-Report | 30 grdauate students and PhD-Level psychologists | M = 29.36, SD = 8.23 | not indicated | | | X | X | | | | | | | | | | | 2 |
| | Hopwood et al. (2018) | Self-Report | 1976 adults recruited through MTurk | | Female (46.4%) Male (53.6%) | | X | | | X | X | X | | X | | | | | | 5 |

| Instrument | Reference | Study Type | Study Population | Age (range [R] and/or Mean [M] with Standard Deviation [SD]) | Gender | Internal Consistency | Test Re-Test Reliability | Interratter Reliability | Content Validity | Construct Validity | Discriminant/Divergent Validity | Convergent Validity | Predictive Validity | Factor Analysis/Principal Component Analysis | Confirmatory Factor Analysis | Structural Equation Modeling | Control/Correlation with Social Desirability | Other Advanced Statistics | Other Biased Responding or Lie Scale | Total Psychometric Assessments |
|---|---|---|---|---|---|---|---|---|---|---|---|---|---|---|---|---|---|---|---|---|
| Levenson Self-Report Psychopathy Scale | Salekin et al. (2014) | Self-Report | 1257 undergraduate students | | Female (69%) Male (31%) | | | | | | X | X | | | X | | | | | 3 |
| Limited Prosocial Emotions Questionnaire (LPEQ) | | | | | | X | | | X | X | | X | | X | | | | | | 5 |
| | Castagna et al. (2021) | Self-Report | 1050 caregivers of children | 20-72 | 470 male, 580 female | X | | | | X | | X | | X | | | | | | 4 |
| | Castagna et al. (2020) | Self-Report | 1050 children | R: 5-12 | Female (48%) Male (52%) | X | | | X | X | | X | | | | | | | | 4 |
| Machiavellian Personality Scale (MPS) | Miller et al. (2015) | Self-Report | 282 adults | | Female (57.7%) Male (42.3%) | | | | | | | | | X | | | | | | 1 |
| Marital Communication Inventory (MCI) | Schumm et al. (1983) | Self-Report | 238 women | | Female (100%) Male (0%) | X | | | | X | | X | | | | | X | | | 4 |
| Measure of Caring in Nursing as Communicative Action [Unnamed] | Sumner & Fisher (2008) | Self-Report | 82 nurses | Almost 60% were aged between 31 and 50 | Female (85.4%) Male (7.3%) Other (7.3%) | X | | | | | | | | X | | | | | | 2 |
| Measure of Orientations Toward Empathic and Sympathetic Care | Hojat et al. (2011) | Self-Report | 258 medical students | Third-year medical students at Jefferson Medical College responded to four clinical vignettes in 2010. | N/a | X | | | | X | | X | | X | | | | | | 4 |
| Measure of Relational Climate [Unnamed] | Boyatzis & Rochford (2020) | Self-Report | 93 adults recruited through MTurk (Sample 1), 287 employed adults recruited through MTurk (Sample 2, EFA), 359 employed adults recruited through MTurk (Sample 3, CFA), 354 employed adults recruited through MTurk (Sample 4) (N=1093) | | Female (55%) Male (45%) | X | | | X | X | X | X | | X | X | X | | | X | 9 |
| Medical Condition Regard Scale (MCRS) | Williams et al. (2013b) | Self-Report | 783 undergraduate paramedic students | | Female (57.3%) Male (42.7%) | X | | | | X | | | | X | X | | | | X | 5 |
| Medical Humanities Questionnaire | Wang (2011) | Self-Report | 449 adults | R: younger than 20 to over 51 | Female (58.6%) Male (41.4%) | X | | | X | X | | | | X | | | | | | 4 |
| Mental States Task (MST) | Beaulieu-Pelletier et al. (2013) | Self-Report | 470 French Canadian undergraduate and graduate students (Study 1), 298 Canadian undergraduate students (Study 2) (N=768) | Study 1: M = 27.27 Study 2: M = 26.61 | N/a | | | | | | | | | X | X | | | | | 2 |
| Mentalized Affectivity Scale (MAS) | Greenberg et al. (2017) | Self-Report | 2840 adults | R: 18-65 | Female (55%) Male (42%) Other (2%) | X | | | X | X | | X | X | X | | | | | | 6 |
| Military Suicide Attitudes Questionnaire Short-Form (MSAQ-SF) | Cramer et al. (2022) | Self-Report | study 1: 200 treatment-seeking active duty service members; study 2: 1116 all active duty members within the statewide Army National Guard group | study 1: M=26.99 (SD=6.41) | study 1: 77% male, 19.5% female; study 2: 86.1% male, 13.9% female | X | | | | | | | | X | X | X | | | | 4 |
| Millennial Organizational Citizenship Behavior (OCB) Scale | Chou et al. (2021) | Self-Report | study 1: 191 full-time Millennial workers enrolled in business programs; study 2: 345 full-time Millennial employees enrolled in business programs; study 3: 416 full-time Millennial workers | study 1 & 2: M = 28.34, study 3: M = 31.2, | study 1: 102 females, study 2: 203 females, study 3: 189 females | X | | | X | X | X | X | | X | X | X | | | | 8 |
| Mindful Attention Awareness Scale (MAAS) | González-Blanch et al. (2022) | Self-Report | 150 participants with remitted first-episode psychosis | M=20.8, SD=2.9 | 55.3% male | X | X | | | X | X | X | | X | X | X | | X | | 9 |
| Mindful Parenting Inventory for Children (MPIC) | Acet & Oliver (2023) | Self-Report | 90 children | 11-16 | Female (51.9%) | X | | | | | | | | X | X | | | | | 3 |
| Mindful Parenting Inventory for Parents (MPIP) | Acet & Oliver (2023) | Self-Report | 135 birth mothers | 28-57 | Female (100%) | X | | | | X | | X | | X | X | | | | | 5 |
| Mindful Self-Care Scale (MSCS) | | | | | | X | | | X | X | | X | | | X | | | | | 5 |
| | Hotchkiss & Cook-Cottone (2019) | Self-Report | 858 hospice and healthcare professionals | | Females (60.7%) Males (39.3%) | X | | | | X | | X | | | X | | | | | 4 |
| | Cook-Cottone & Guyker (2018) | Self-Report | 448 adults recruited from university class, social media, and MTurk (Sample 1), 452 adults recruited from university class, social media, and MTurk (Sample 2) (N=900) | | Female (74.7%) Male (25.3%) | X | | | X | X | | X | | | X | | | | | 5 |
| Minnesota Multiphasic Personality Inventory-2 (MMPI-2) | | Self-Report | | | | X | | | | X | | X | | X | X | | | | | 5 |
| | Sweeney et al. (2021) | Self-Report | 179 mental health patients | M = 46.6 | 61.5% female | X | | | | X | | X | | X | X | | | | | 5 |
| | Williams, Dalgleish, Karl, & Kuyken (2014) | Self-Report | | | Female (68%) Male (32%) | X | | | | | | | | | X | | | | | 2 |
| | Rushton & Irwing (2009) | Self-Report | | | Female (56%) Male (44%) | X | | | | | | | | X | X | | | | | 3 |
| Miville-Guzman Universality-Diversity Scale | Miville et al. (1999) | Self-Report | 93 undergraduate students (Study 1), 111 undergraduate students (Study 2), 153 undergraduate students (Study 3), 135 undergraduate students (Study 4) (N=492) | | Study 1: Female 28%, Male 72% Study 2: Female 30%, Male 70% Study 3: Female 35%, Male 65% Study 4: Female 71%, Male 29% | X | X | | | | X | | | | | | | | | 3 |
| Modified Version of Empathic Accuracy Task (EAT) | Mackes et al. (2018) | Self-Report | 94 adults | | Female (58.6%) Male (41.4%) | | | | | X | | X | | | | | | X | | 3 |
| Moral Courage Scale for Physicians (MCSP) | Martinez et al. (2016) | Self-Report | 352 medical interns and residents | | Female (49%) Male (50%) | X | | | | X | | | | X | | | | | | 3 |
| Moral Expansiveness Scale (MES) | Crimston et al. (2016) | Self-Report | 117 adults recruited through MTurk (Study 1), 119 adults recruited through MTurk (Study 2), 289 adults recruited through MTurk (Study 3), 91 adults recruited through MTurk (Study 4), 87 adults recruited through MTurk (Study 5), 89 Australian undergraduate psychology students (Study 6) (N=792) | | Female (47.62%) Male (52.38%) | X | | | | | | X | X | | | | X | | | 4 |
| Moral Foundations Questionnaire (MFQ) | Graham et al. (2011) | Self-Report | 34476 adults | | Female (37%) Male (63%) | X | X | | X | X | X | X | X | X | X | X | | | | 10 |
| Moral Foundations Questionnaire - Revised (MFQ-r) | Harper & Rhodes (2021) | Self-Report | study 1: 428 participants; study 2: 322 participants | study 1: M = 33.54. SD=14.13; study 2: M=39.79, SD=14.80 | study 1: 70% male; study 2: 61% female | X | | | | | | | X | X | X | | | | | 4 |
| Moral Inclusion/Exclusion of Other Groups Scale (MIEG) | Passini & Morselli (2017) | Self-Report | 1345 adults | | Female (56.1%) Male (43.9%) | X | | | X | X | X | X | X | X | X | | | | | 8 |
| Moral Mosaic - Short Form | Bell & Showers (2021) | Self-Report | 449 undergraduates | M=18.7 (SD=1.56) | 333 female | | | | | | | | X | X | X | X | | | | 4 |
| Multicultural Personality Questionnaire (MPQ) | | | | | | X | | | | X | | | | X | X | | | | | 4 |
| | Rushton & Irwing (2009) | Self-Report | | | Female (69%) Male (31%) | X | | | | | | | | X | X | | | | | 3 |
| | Van Oudenhoven & Van Der Zee (2002) | Self-Report | 171 international business school students | | Female (54%) Male (46%) | X | | | | X | | | | | | | | | | 2 |
| Multicultural Personality Questionnaire Short Form (MPQ-SF) | Hofhuis et al. (2020) | Self-Report | 519 first-year students of an international English-language bachelor program | M=19.1, SD=1.58 | 60.5% female, 14.5% male, 25% other | | X | | | | | | | X | | X | | | | 3 |
| Multi-Dimensional Support Scale (MDSS) | Neuling & Winefield (1988) | Self-Report | 58 breast cancer patients with no previous life threatening illness | | Female (100%) Male (0%) | X | | | | | | | | | | | | | | 1 |
| Multifaceted Empathy Test (MET) | | | | | | X | | | | X | X | X | | X | | | | | | 5 |
| | Peretti et al. (2019) | Self-Report | | | | | | | | | X | X | | X | | | | | | 3 |
| | Foell et al. (2018) | Self-Report | 19 undergraduate students (Development Study), 80 undergraduate students (Validation Study) (N=99) | | Female (72.5%) Male (27.5%) | X | | | | X | | X | | | | | | | | 3 |
| My Emotions Questionnaire | | | | | | X | | | | X | X | X | X | X | X | | | | | 7 |
| | Leerkes &  Qu (2020) | Self-Report | 259 primiparous mothers | M=25 | 100% women | X | | | | X | X | X | X | X | X | | | | | 7 |
| | Klamann et al. (2019) | Self-Report | 240 first-time mothers | | Female (100%) Male (0%) | X | | | | | | | | X | X | | | | | 3 |
| Narnian Character Virtue Scales (NCVS) | Francis et al. (2017) | Self-Report | 56 adolescent students | R: 12-13 | Female (55.3%) Male (44.7%) | X | | | | X | | | | | | | | | | 2 |
| Non-Verbal Compassionate Communication Questionnaire | Mcelroy & Esterhuizen (2017) | Self-Report | 20 undergraduate or postgraduate nursing students (Lay Expert Sample), 3 nursing experts (Research Expert Sample) (N=23) | | N/a | | | | X | | | | | | | | | | | 1 |

| Instrument | Reference | Study Type | Study Population | Age (range [R] and/or Mean [M] with Standard Deviation [SD]) | Gender | Internal Consistency | Test Re-Test Reliability | Interratter Reliability | Content Validity | Construct Validity | Discriminant/Divergent Validity | Convergent Validity | Predictive Validity | Factor Analysis/Principal Component Analysis | Confirmatory Factor Analysis | Structural Equation Modeling | Control/Correlation with Social Desirability | Other Advanced Statistics | Other Biased Responding or Lie Scale | Total Psychometric Assessments |
|---|---|---|---|---|---|---|---|---|---|---|---|---|---|---|---|---|---|---|---|---|
| Nurse-Nursing Assistant-Caregiver Reciprocity Scale (NNA-CRS) | Ameia Yen-Patton et al. (2013) | Self-Report | 1050 nursing assistant caregivers | | N/a | X | | | X | | | | | X | | | | | | 3 |
| Nurses' Confidence Scale: Unique Families (NCSUF) | Grassley et al. (2020) | Self-Report | 62 nurses | | N/a | X | | | X | X | | X | | X | | | | | | 5 |
| Nursing Dimensions Inventory (NDI) | Watson (2003) | Self-Report | 38 nursing lecturers | | N/a | | | X | | | | | | | | | | | | 1 |
| Nursing Motives for Helping Scale (N-MHS) | Nassar (2021) | Self-Report | 361 nurses | M=33.4 (SD=7.4) | 81.4% females | X | | | | X | | X | | X | X | | | | | 5 |
| Nursing Shared Decision-Making Attitude Scale (NSDMA) | Hsu et al. (2021) | Self-Report | 451 nursing staff | M=42.01, SD=10.38 | not indicated | X | | | X | X | X | X | | X | X | X | | | | 8 |
| Open-Minded Cognition Scale [Unnamed] | Price et al. (2015) | Self-Report | 219 adults | | Female (47.7%) Male (52.3%) | X | | | | | X | X | X | | X | | | | | 5 |
| Orientation Model | Wilkinson (2019) | Self-Report | 375 undergraduate students | | Female (66.9%) Male (33.1%) | X | | | | X | | | | | | | | X | | 3 |
| OSPA Inventory | Washburne (1935) | Self-Report | 3114 adults | | N/a | X | | | X | | | | | | | | | | | 2 |
| Other Dyadic Perspective-Taking Scale (ODPT) | Long (1990) | Self-Report | | | | X | X | | | X | | | | X | | | | X | | 5 |
| Parental Empathy Measure (PEM) | Kilpatrick (2005) | Self-Report | 103 parents | | Female (88%) Male (12%) | X | | X | | X | X | X | | | | | X | | | 6 |
| Partner Synchrony Inventory (PSI) | Kahans et al. (1997) | Self-Report | 54 couples presenting for marital therapy (N=108) | | N/a | X | | | | | | X | | X | | | | | | 3 |
| Patient Experience Survey in Radiation Therapy | Hashmi et al. (2019) | Self-Report | 10 patients receiving radiation therapy treatment (Development Study), 86 patients receiving radiation therapy treatment (Validation Study) (N=96) | | N/a | | | | | | | | | | | | | | | 0 |
| Patient-Physician Relationship Scale - Physician (PPRS-Physician) | Feingold et al. (2021) | Self-Report | 1113 physicians | M=55.1 | 85% male | X | | | X | X | | X | | X | | | | | | 5 |
| Patient-Reported Compassion Measure [Unnamed] | Sinclair et al. (2020) | Self-Report | 33 (8 SME, 9 PAG, 16 patients) | not indicated | SME: 62.5% male, 37.5% female; PAG: 44.4% male, 55.5% female, patients 62.5% male, 37.5% female | | | | X | | | | | | | | | | | 1 |
| Patient Satisfaction Scale | Westaway et al. (2002) | Self-Report | 263 black diabetic outpatients | | Female (66.1%) Male (33.9%) | X | | | | | X | X | | X | | | | | | 4 |
| Pediatric-Hematogoloy Oncology Fellowship Training Skills Measure [Unnamed] | Kesselheim et al. (2017) | Self-Report | 90 hematology–oncology fellows | | N/a | X | | | | | | X | | | | | | | | 2 |
| Peer Edmonton Empathy Recruitment Scale (PEERS) | Dubue et al. (2018) | Self-Report | 1439 Canadian undergraduate students | | Female (65.5%) Male (34.5%) | X | X | | | | | X | | X | | | X | | | 5 |
| Perceived Empathic and Social Self-Efficacy Scale (PESE) | Sánchez et al. (2016) | Self-Report | 194 adults with primary diagnosis of serious mental illness | 18+ | Female (53.1%) Male (46.9%) | X | | | | X | | | | | X | | | | | 3 |
| Person-Centered Care Assessment Tool (P-CAT) | Gordon et al. (2022) | Self-Report | 35 aged care workers | not indicated | not indicated | X | X | X | X | | | | | | | | | | | 4 |
| Person-Centred Caring: Participation [Unnamed] | Strachan et al. (2020) | Self-Report | 705 patients | M = 76, SD = 14 | 37% male,62% female | X | | | X | X | | X | | X | | | | | | 5 |
| Person-Centred Caring: Personalization [Unnamed] | Strachan et al. (2020) | Self-Report | 796 patients | M = 68, SD=17 | 37% male, 62% female | X | | | X | X | | X | | X | | | | | | 5 |
| Person-Centred Caring: Responsiveness [Unnamed] | Strachan et al. (2020) | Self-Report | 644 patients | M = 70, SD = 17 | 45% male, 51% female | X | | | X | X | | X | | X | | | | | | 5 |
| Personal Orientation Inventory (POI) | | | | | | | | | | X | | | | | | | | | | 1 |
| | Leak (1984) | Self-Report | 90 undergraduate psychology students | R: 18-28 | N/a | | | | | X | | | | | | | | | | 1 |
| | Fischer & Knapp (1977) | Self-Report | 30 graduate social work students | | N/a | | X | X | | | | | | | | | | X | | 3 |
| Personal Strengths Inventory (PSI) | Kienfie Liau et al. (2011) | Self-Report | 410 adolescents in Singapore (Study 1), 598 adolescents in Singapore (Study 2) (N=1008) | | | X | | | | | | X | | X | X | | | | | 4 |
| Personal Trust and Connections scale (PerTC) | Symvoulakis et al. (2022) | Self-Report | 218 health care providers | M = 14.8, SD = 10.5 | 72.9% women | X | | | X | X | | X | | X | | | | | | 5 |
| Personality Assessment Inventory-Borderline Features Scale (PAI-BOR) | Waugh et al. (2021) | Self-Report | 28 grdauate students and PhD-Level psychologists | M = 29.36, SD = 8.23 | not indicated | | | X | X | | | | | | | | | | | 2 |
| Peace Attitude Scale (PAS) | Broccoli et al. (2021) | Self-Report | 499 adolescents and adults | M = 23.04, SD = 2.345 | 199 females, 274 males, 26 not specified | X | X | | X | X | X | X | | X | | | | | | 7 |
| Perth Empathy Scale (PES) | Brett et al. (2023) | Self-Report | study 1: 316 undergraduates study 2: 331 undergraduates | study 1: 32.17 mean age SD = 12.41 study 2: 16-56, M = 20.82, SD = 5.33 | study 1: Female 84.8%, Male 15.2% study 2: Female 55.6%, Male 44.1% | X | | | | X | X | X | | X | X | | | X | | 7 |
| Pharmacists' Care of Migraineurs Scale (PCMS) | Skomo et al. (2009) | Self-Report | 580 pharmacists | | Female (38.3%) Male (61.7%) | X | | | | X | | | | | | | | | | 2 |
| Phenomenological Body Shame Scale - Revised (PBSS-R) | Siegel et al. (2021) | Self-Report | study 1: 350 women participants; study 2: 241 women psychology students; study 3: 235 women participants | study 1: M=31.14, SD=11.47; study 2: M=19.27, SD=1.43; study 3: M=37.51, SD=11.07 | 100% women | X | | | | X | X | X | | X | X | | | | | 6 |
| Posttraumatic Growth Inventory (PTGI) | Morris et al. (2013) | Self-Report | 514 prostate cancer survivors | | Female (0%) Male (100%) | | | | | | | X | | X | X | | | | | 3 |
| Pregnant Women with HIV Attitude Scale | Tyer-Viola & Duffy (2010) | Self-Report | 209 obstetric nurses | | Female (97.6%) Male (2.4%) | X | | | X | | | | | X | | | | | | 3 |
| Prejudice Confrontation Styles (PCS) Scale | Chaney & Sanchez (2022) | Self-Report | study 1: 388 undergraduates; study 2: 276 undergraduates; study 3: 248 US undergraduate women | study 1: M=19.29, SD=2.48; study 2: M=18.84, SD=1.46; study 3: M=18.52, SD=1.13) | study 1: 59.5% women, 40.5% men; study 2: 71.7% women, 28.3% ment; study 3: 100% women | X | X | | X | X | X | X | X | X | X | X | | | | 10 |
| Prejudice toward People with Borderline Personality Disorder (PPBPD) Scale | Sheppard et al. (2023) | Self-Report | sample1: 217 Australian and New Zealand students; sample 2: 303 Australian undergraduate psychology students; sample 3: 314 general public | study 1: M=26.8, SD=8.13; study 2: M=21.6, SD=2.57; study 3: M=29.75, SD=10.57 | sample 1: 82% female; sample 2: 59% female; sample 3: 51% female | | | | | X | | X | | X | X | | | | | 4 |
| Prejudice toward People with Depression (PPD) | Bizumic & Gunningham (2022) | Self-Report | 406 participants | M = 27.43, SD = 10.76 | 64% female | X | | | X | X | X | X | | X | X | X | | | | 8 |
| Prejudice toward People with Schizophrenia (PPS) | Bizumic & Gunningham (2022) | Self-Report | 406 participants | M = 27.43, SD = 10.76 | 64% female | X | | | X | X | X | X | | X | X | X | | | | 8 |
| Princess Margaret Hospital Patient Satisfaction with Doctor Questionnaire (PMH/PSQ-MD) | Loblaw et al. (1999) | Self-Report | 277 cancer patients | | Female (74%) Male (26%) | | | | X | | X | X | | X | | | | | | 4 |
| Professional Quality of Life Scale (ProQoL) | | | | | | X | | | | X | X | X | | X | X | | | | | 6 |
| | Keesler & Fukui (2020) | Self-Report | 495 direct support professionals | | Female (79.8%) Male (19.5) | X | | | | | | | | X | | | | | | 2 |
| | Geoffrion et al. (2019) | Self-Report | 310 child protection workers | | Female (84.5%) Male (15.5%) | X | | | | X | X | X | | | X | | | | | 5 |
| Professional Quality of Life - Revised (ProQOL-21) | Ahmed et al. (2022) | Self-Report | 375 physicians | 52.43 mean age | Female (42.4%) | | | | | X | X | | | X | X | | | | | 4 |
| Professional Quality of Life Scale - Version 5 (ProQoL 5) | Hemsworth et al. (2018) | Self-Report | 273 Australian nurses (Sample 1), 303 Canadian nurses (Sample 2), 503 Canadian palliative care workers (Sample 3) (N=1079) | | Female (87.2%) Male (12.8%) | X | | | | X | X | X | | X | X | | | | | 6 |
| Professionalism Assessment Scale (PAS) | Klemenc-Ketis & Vrecko (2014) | Self-Report | 12 medical students (Focus Groups), 20 family medicine experts (Delphi Method), 122 medical students (Psychometrics) (N=154) | | | X | | | X | X | | | | X | | | | X | | 5 |
| Prosocial and Antisocial Behavior in Sport Scale (PABSS) | | | | | | X | X | | X | X | X | X | | X | X | | | X | | 9 |
| | Kavussanu et al. (2013) | Self-Report | 129 active athletes | | Female (43.4%) Male (56.6%) | X | X | | | X | X | X | | | | | | | | 5 |
| | Kavussanu & Boardley (2009) | Self-Report | 1213 team-sport athletes (Study 1), 106 team-sport athletes (Study 2) (N=1319) | | Female (46%) Male (54%) | | | | X | X | X | X | | X | X | | | X | | 7 |
| Prosocial Personality Battery (PSP) | Maki et al. (2017) | Self-Report | 363 adults | | Female (43.6%) Male (56.4%) | X | | | | X | X | X | | X | X | | | X | | 7 |
| Prosocial Risk-Taking Scale (PSRT) | Armstrong-Carter et al. (2021) | Self-Report | 867 adolescents | 10-14 | Female (50.11%) | X | X | | X | X | | X | | X | X | | | | | 7 |
| Psychopathic Personality Inventory - Short Form (PPI-SF) | Adams et al. (2020) | Self-Report | 1257 undergraduate introductory psychology students | 17-51 | Male (30.1%) Female (69.1%) | | | | | X | X | X | | X | X | | | | | 5 |
| Psychosocial Costs of Racism to Whites Scale (PCRW) | | | | | | X | X | | | X | X | X | | X | X | | X | | | 8 |
| | Poteat & Spanierman (2008) | Self-Report | 284 employed white adults | | Female (51%) Male (49%) | X | | | | X | X | X | | | X | | | | | 5 |
| | Spanierman & Heppner (2004) | Self-Report | 393 undergraduate students | | N/a | X | X | | | X | | X | | X | | | X | | | 6 |

| Instrument | Reference | Study Type | Study Population | Age (range [R] and/or Mean [M] with Standard Deviation [SD]) | Gender | Internal Consistency | Test Re-Test Reliability | Interratter Reliability | Content Validity | Construct Validity | Discriminant/Divergent Validity | Convergent Validity | Predictive Validity | Factor Analysis/Principal Component Analysis | Confirmatory Factor Analysis | Structural Equation Modeling | Control/Correlation with Social Desirability | Other Advanced Statistics | Other Biased Responding or Lie Scale | Total Psychometric Assessments |
|---|---|---|---|---|---|---|---|---|---|---|---|---|---|---|---|---|---|---|---|---|
| Psychosocial Inventory of Ego Strengths (PIES) | Markstrom et al. (1997) | Self-Report | 244 undergraduate students (Study 1), 153 undergraduate students (Study 2) (N=397) | | Study 1: Female (100%) Male (0%) Study 2: Female (59%) Male (41%) | X | | | X | | | X | | | | | | | | 3 |
| Public Attitudes Towards Homelessness (PATH) | Guzewicz & Takooshian (1992) | Self-Report | 222 adults | | Female (50%) Male (50%) | X | | | | X | | | | X | | | X | X | | 5 |
| Questionnaire of Cognitive and Affective Empathy (QCAE) | | | | | | X | | X | | X | X | X | X | X | X | X | | X | | 10 |
| | Gomez et al. (2022) | Self-Report | 203 adults | M=30.99, SD=11.79 | 43 men, 160 women | X | | | | X | | | | X | X | X | | X | | 6 |
| | Brunet et al. (2021) | Self-Report | 143 patients with schizophrenia or schizoaffective disorder | M = 31.4, SD =8.2 | 77.6% male | X | | X | | X | X | X | X | | | | | | | 6 |
| | Reniers et al. (2011) | Self-Report | 640 adults (Study 1), 318 (Study 2) (N=958) | | Study 1: Female (68.2%) Male (31.8%) Study 2: Female (72.3%) Male (27.7%) | | | | | X | | X | | X | X | | | | | 4 |
| Questionnaire Measure of Empathic Tendency | Mehrabian & Epstein (1972) | Self-Report | 88 undergraduate students | | Female (58%), Male (42%) | | | | X | X | | | | | | | | | | 2 |
| Range and Differentiation of Emotional Experience Scale (RDEES) | Kang & Shaver (2004) | Self-Report | 400 undergraduate psychology students (Study 1), 729 undergraduate psychology students (Study 2) (N=1129) | R: 17-51, M = 19.6 years, SD = 2.38) | N/a | X | | X | | X | X | X | | X | | | X | | | 7 |
| Rating Scales for the Assessment of Empathic Communication in Medical Internviews (REM) | Nicolai et al. (2010) | Self-Report | 118 German undergraduate students (Study 1), 23 German physicians (Study 2) (N=141) | Study 1: M = 25.4 Study 2: R: 35-58 | Female (70.8%) Male (29.2%) | | | X | | | | X | | X | | | | | | 3 |
| Reading the Mind in the Eyes Test (RMET) | | | | | | X | | X | | X | X | X | X | X | X | X | | | | 9 |
| | Kittle et al. (2022) | Self-Report | N/A | N/A | N/A | X | | X | | X | X | X | X | X | | X | | X | | 9 |
| | Olderbak et al. (2015) | Self-Report | 484 adults recruited through MTurk | | Female (50%) Male (50%) | X | | | | | | X | | X | X | | | | | 4 |
| Reflective Functioning Questionnaire | Fonagy et al. (2016) | Self-Report | 108 patients with comorbid Borderline Personality Disorder and Eating Disorders, 295 undergraduate students and univeristy staff (N=403) | | Female (66.4%) Male (33.6%) | X | X | | | X | | X | | | X | X | | | | 6 |
| Relational Care Scale (RCS) | | | | | | X | X | X | X | X | X | X | | X | | | | | | 8 |
| | Andersen & Havaei (2015) | Self-Report | 62 nursing home residents | | N/a | X | | | | X | X | X | | X | | | | | | 5 |
| | McGilton et al. (2005) | Self-Report | 30 nursing staff members (Sample 1), 50 nursing staff members (Sam | | Female (67.8%) Male (32 | X | X | X | X | X | X | | | X | | | | | | 7 |
| Relational Competence for Community-Dwelling Elderly | Hogg & Heller (1990) | Self-Report | 291 low-income, community-dwelling elderly women | | Female (100%) Male (0%) | X | | | X | | X | X | X | X | | | | | | 6 |
| Relational Depth (RD) Inventory | Wiggins et al. (2012) | Self-Report | 189 therapists, 152 clients, 2 uncategorized (N=343) | | Female (74.9%) Male (23.3%) Other (1.2%) | X | | X | | X | X | X | | X | | | | X | | 7 |
| Relational Engagement of the Sacred for a Transgression (REST) Scale | Davis et al. (2010) | Self-Report | 200 undergraduate students | | Female (59%) Male (41%) | X | | | | X | | | X | X | X | X | X | X | | 8 |
| Relationship Inventory (RI) | | | | | | | | | | | | | | X | | | | X | | 2 |
| | Cramer (1986) | Self-Report | 169 guraduate students | | Female (68.6%) Male (31.4%) | | | | | | | | | X | | | | X | | 2 |
| | Schumm et al. (1981) | Self-Report | 98 urban married couples (N=196) | | Female (50%) Male (50%) | | | | | | | | | X | | | | | | 1 |
| Response Empathy Rating Scale | Elliott et al. (1982) | Self-Report | 28 counselor-client interactions rated by trained undergraduate students | | N/a | X | | | | | | X | | | | X | | | | 3 |
| Restorative Justice Attitudes Scale (RJAS) | Taylor & Bailey (2022) | Self-Report | study 1: 315 participants; study 2: 335 participants | study 1: M = 35.08, SD = 12.45; study 2: M = 34.87, SD = 15.8 | study 1: 21% man, 66% woman; study 2: 22% man, 65% woman | X | | | X | X | | X | | X | X | | | | | 6 |
| Revised Humane Caring Scale (RHCS) | Goh et al. (2015) | Self-Report | 235 patients | | Female (47%) Male (53%) | X | X | | X | X | | | | | | | | | | 4 |
| Revised Sense of Purpose Scale (SOPS-2) | Yukhymenko-Lescroart & Sharma (2020 | Self-Report | study 1: 511 staff and students; study 2: 403 university students; study 3: 297 instructors and staff | study 1: students: M=33.6, SD=7.99, staff: M=49.71, SD=12.27; study 2: M=32.75, SD=9.72; study 3: M=46.87, SD=12.11 | study 1: 72.3% female students, 64.8% female staff; study 2: 66% female; study 3: 66.9% female | X | | | | X | X | X | | X | X | X | | | | 7 |
| Safety Skills Questionnaire [Unnamed] | Ahmed et al. (2014) | Self-Report | 24 general practitioner educational supervisors | | N/a | | | | X | | | | | | | | | | | 1 |
| San Diego Wisdom Scale (SD-WISE) | Thomas et al. (2019) | Self-Report | 39 adults | R: 27–76 | Female (46%) Male (54%) | | | | | | | | | | | | | X | | 1 |
| San Diego Wisdom Scale (SD-WISE-7) | Thomas et al. (2022) | Self-Report | 2093 participants | M = 46.3, SD = 14.6 | 55% women | X | | | | X | | X | | X | X | | | | | 5 |
| Santa Clara Brief Compassion Scale (SCBCS) | | | | | | X | X | | | X | X | X | | X | | | | | | 6 |
| | Plante & Mejia (2016) | Self-Report | 6763 undergraduate students | | Female (62.7%) Male (37.3%) | X | X | | | X | X | X | | X | | | | | | 6 |
| | Hwang et al. (2008) | Self-Report | 223 undergraduate students | | Female (75%) Male (25%) | X | | | | | | | | X | | | | | | 2 |
| Santa Clara Ethics Questionnaire (SCES) | Plante & McCreadie (2019) | Self-Report | 329 undergraduate students | | Female (70%) Male (30%) | | | | | | | | | | | | | X | | 1 |
| Satisfaction with Simulation Experience Scale (SSES) | Courtney-Pratt et al. (2015) | Self-Report | 497 nursing students | | Female (88.1%) Male (11.9%) | X | | | | | | | | X | X | | | X | | 4 |
| Scale of Ethnocultural Empathy (SEE) | | | | | | X | | | | X | X | X | | | X | X | | X | | 7 |
| | Fleming et al. (2015) | Self-Report | 136 nursing and dental students | | N/a | X | | | | | | | | | | | | | | 1 |
| | Albiero & Matricardi (2013) | Self-Report | 610 Italian undergraduate students | | Female (63.1%) Male (36.9%) | X | | | | X | X | X | | | X | X | | X | | 7 |
| | Wang et al. (2003) | Self-Report | 323 undergraduate students | | Female (66%) Male (34%) | | | | | | | | | | X | | | | | 1 |
| Scale of Humanistic Nursing Behaviors | Fenton (1987) | Self-Report | 316 nursing staff | | N/a | X | X | | | X | X | | | | | | | | | 4 |
| Scale of Teacher Empathy for African American Males (S-TEAAM) | Warren (2015) | Self-Report | 73 practicing classroom teachers | | N/a | X | | | | | | | | X | | | | | | 2 |
| Schadenfreude Scale | Crysel & Webster (2018) | Self-Report | 802 undergraduate students and adults recruited through MTurk (Study 1), 192 adults recruited through MTurk (Study 2), 28 undergraduate psychology students (Study 3), 261 adults recruited through MTurk (Study 4), 304 adults recruited through MTurk (Study 5) (N=1587) | R: 18-71 | Female (48.5%) Male (49.3%) Other (2.2%) | | X | | | | | X | | X | X | | | | | 4 |
| Schema of Partner Empathic Responses to Anger (SOPERA) | Sanford (1998) | Self-Report | 74 married individuals | | Female (50%) Male (50%) | | X | | | | | | | | | | | | | 1 |
| School Support Scale | McLaughlin & Randolph (2012) | Self-Report | 240 student-mothers | | Female (100%) Male (0%) | X | | | X | X | X | X | X | X | X | | | | | 8 |
| Secondary Traumatic Stress Scale (STSS) | Ahmed et al. (2022) | Self-Report | 375 physicians | 52.43 mean age | Female (42.4%) | | | | | X | X | | | X | X | | | | | 4 |
| Self and Interpersonal Functioning Scale (SIFS) | Gamache et al. (2019) | Self-Report | 280 Canadian adults (Community Sample), 106 individuals seeking personality disorder treatment at an outpaitent psychiatric clinic (Clinical Sample) (N=386) | | N/a | | | | X | | | | | X | | | | | | 2 |
| Self-Compassion and Self-Criticism Scales (SCCS) | Falconer et al. (2015) | Self-Report | 413 adults (Study 1), 90 adults (Study 2) (N=503) | | Female (61.6%) Male (38.4%) | X | | | | X | | X | | | | X | | | | 4 |
| Self-Compassion Scale (SCS) | | | | | | X | X | | X | X | X | X | X | X | X | X | X | X | | 12 |
| | Rakhimov et al. (2023) | Self-Report | 486 | M age = 39.1 years; SD = 19.2; range 18 to 85 | 196 men; 287 women; 2 gender-nonconforming people | - | | | | | | X | | X | X | X | | | | 4 |
| | Biehler & Naragon-Gainey (2022) | Self-Report | 172 community participants, over-sampled for individuals receiving or seeking mental health treatment | M = 35.1 (SD = 13.67) | 71% female | X | | | | X | X | X | X | X | X | X | | | | 8 |
| | Finaulahi et al. (2021) | Self-Report | 7443 participants | M=30.44, SD=11.33 | 31.8% male | X | | | | X | | | | X | | X | | | | 4 |
| | Tóth-Király & Neff (2020) | Self-Report | 10997 international respondents | R: 15-83, M = 31.16, SD = 13.45 | Female (69.4%) Male (30.6%) | - | | | | | | | | | | X | | | | 1 |
| | Zhang et al. (2019) | Self-Report | 248 individuals with a recent suicide attempt | | Female (56%) Male (44%) | X | | | | | | X | | | X | | | | | 3 |

| Instrument | Reference | Study Type | Study Population | Age (range [R] and/or Mean [M] with Standard Deviation [SD]) | Gender | Internal Consistency | Test Re-Test Reliability | Interratter Reliability | Content Validity | Construct Validity | Discriminant/Divergent Validity | Convergent Validity | Predictive Validity | Factor Analysis/Principal Component Analysis | Confirmatory Factor Analysis | Structural Equation Modeling | Control/Correlation with Social Desirability | Other Advanced Statistics | Other Biased Responding or Lie Scale | Total Psychometric Assessments |
|---|---|---|---|---|---|---|---|---|---|---|---|---|---|---|---|---|---|---|---|---|
| | Neff et al. (2019) | Self-Report | 11685 adults | R: 18-83 | Female (71.6%) Male (28.2%) Other (0.19%) | X | | | | | | | | | X | | | | | 2 |
| | Cleare et al. (2018) | Self-Report | 526 adults | R: 16-64 | Female (76%) Male (24%) | X | X | | X | X | | | | | X | | | | | 5 |
| | Tanenbaum et al. (2018) | Self-Report | 542 adults diagnosed with Typer 1 Diabetes | M = 41, SD = 15.7 | Female (65%) Male (35%) | X | | | | X | | X | | | X | | | | | 4 |
| | Neff et al. (2017) | Self-Report | 222 undergraduate students (Student Sample), 1394 adults recruited through MTurk (Community Sample), 215 individuals practicing Buddhist meditation (Meditator Sample), 390 individuals with a history of recurrent depression (Clinical Sample) (N=2221) | | Female (60.5%) Male (39.5%) | X | | | | | | | | X | | | | X | | 3 |
| | Pinciotti et al. (2017) | Self-Report | 322 undergraduate students exposed to a past traumatic event | | Female (72%) Male (28%) | X | | | | X | X | X | | | X | X | | | | 6 |
| | Williams, Dalgleish, Karl, & Kuyken (2014) | Self-Report | | | | X | | | | | | | | | X | | | | | 2 |
| | Kemppainen et al. (2013) | Self-Report | 1967 adults living with HIV disease | | Female (27.2%) Male (72.6%) | X | | | | | | | | X | | | | | | 2 |
| | Neff (2003) | Self-Report | 68 undergraduate students (Pilot Phase 1), 71 undergraduate students (Pilot Phase 2), 391 undergraduate educational-psychology students (Study 1), 232 undergraduate educational-psychology students (Study 2), 43 Buddhist meditation practitioners (Study 3) (N=805) | | Pilot phase 1: Female (44%) Male (56%), phase 2: Female (66%) Male (34%). Study 1: Female (57.6%) male (42.4%). Study 2: Female (62.5%) Male (37.5%). Study 3: Female (63%) Male (37%) | X | | | X | X | X | X | | X | X | | X | | | 8 |
| Self-Compassion Scale - Short Form (SCS-SF) | | | | | | X | X | | | X | X | X | | X | X | X | | X | | 9 |
| | Finaulahi et al. (2021) | Self-Report | 743 participants | M=30.44, SD=11.33 | 31.8% male | X | | | | X | | | | X | | X | | | | 4 |
| | Medvedev et al. (2021) | Self-Report | 124 educators | M=45.39, SD=12 | 85 female | X | X | | | | | | | | | | | X | | 3 |
| | Kotera & Sheffield (2020) | Self-Report | 333 undergraduate students in the U.K. | | N/a | X | | | | X | X | X | | | X | | | | | 5 |
| | Babenko & Guo (2019) | Self-Report | 200 medical students | | Female (60%) Male (40%) | X | | | | | | | | X | X | | | | | 3 |
| | Hayes et al. (2016) | Self-Report | 1609 psychotherapy clients | | Female (69%) Male (31%) | | | | | X | | | | X | | | | | | 2 |
| | Raes et al. (2011) | Self-Report | 271 adults (Study 1), 185 adults (Study 2) (N=456) | Study 1: R: 18-28, M = 18.14, SD = 1.25 Study 2: R: 18-64, M = 33.04 years, SD = 10.60 | Study 1: Female (78%) Male (22%) Study 2: Female (20%) Male (30%) | X | | | | | | | | X | | | | | | 2 |
| Self-Compassion Scale - Youth Version (SCS-Y) | Neff et al. (2021) | Self-Report | 279 middle school students (Study 1), 402 middle school students (Study 2), 102 middle school students (Study 3), 212 middle school students (Study 4) (N=995) | R: 11-16 | Study 1: Female (56.7%) male (43.3%). Study 2: Female (48.8%), Male (51.2%). Study 3: Female (51%), Male (49%). Study 4: Female (42.5%), Male (57.5%) | X | | | | | | | | X | | | | | | 2 |
| Self-Connection Scale (SCS) | Klussman et al. (2022) | Self-Report | 1469 participants (study 1: 308, study 2: 164, study 3: 997) | study 1: M=37.62, SD=12.21; study 2: M=36.01, SD=11.35; study 3: M=33.72, SD=12.01 | study 1: 47.73% male, 48.70% female; study 2: 51.83% male, 39.02% female; study 3: 40.62% male, 55.97% female, 3.41% non-binary | X | | | X | X | X | X | | X | X | | | | | 7 |
| Self Dyadic Perspective-Taking Scale (SDPT) | Long (1990) | Self-Report | | | | X | X | | | X | | | | X | | | | X | | 5 |
| Self-Efficacy for Mindfulness Meditation Practice Scale | Birdee et al. (2020) | Self-Report | 199 mindfulness meditation practitioners | | Female (74%) Male (26%) | X | X | | | X | | | | X | | | | | | 4 |
| Self-focused Practice Questionnaire (SfPQ) | Chigwedere et al. (2017) | Self-Report | 112 trainee therapists | | Female (75%) Male (25%) | X | | | | | | | | | X | | | | | 2 |
| Self-Other Four Immeasurables Scale (SOFI) | Kraus & Sears (2008) | Self-Report | 124 undergraduate students (Sample 1), 12 experienced meditators (Sample 2) (N=136) | | Female (52%) Male (48%) | X | | | | X | X | X | | X | | | X | | | 6 |
| Self versus Other Interest Implicit Association Test (SOI-IAT) | Thornton & Aknin (2020) | Self-Report | 153 Canadian blood donors | R: 19-77, M = 41.7, SD = 15.5 | Female (40%) Male (60%) | | | | | X | | X | | | | | | | | 2 |
| Semi-Structured Interview for Personality Functioning DSM–5 (STiP-5.1) | Hutsebaut et al. (2017) | Self-Report | 80 Dutch adults seeking treatment for personality pathology (Clinical Sample), 18 Dutch adults (Community Sample) (N=98) | | Female (66.3%) Male (33.7%) | X | | X | | X | | X | | | | | | | | 4 |
| SERVQUAL (SERVice QUALity) | | | | | | | | | | X | | X | X | X | X | | | | | 5 |
| | Kang et al. (2002) | Self-Report | 120 university employees | | Female (33%) Male (67%) | | | | | | | | | | X | | | | | 1 |
| | Konerding et al. (2019) | Self-Report | 1884 European adults diagnosed with Type 2 Diabetes or a Stroke | 18+ | N/a | | | | | | | X | | | | | | | | 1 |
| | Tomes & Chee Peng Ng (1995) | Self-Report | 20 hospitalized patients (Pilot Sample), 132 hospitalized patients (Validation Sample) (N=152) | | N/a | | | | | | | | | X | | | | | | 1 |
| | Soliman (1992) | Self-Report | 463 employed adults | R: 25 and older | N/a | | | | | X | | X | X | | | | | | | 3 |
| Severity Indices of Personality Problems-Short Form (SIPP-SF) | Waugh et al. (2021) | Self-Report | 26 grdauate students and PhD-Level psychologists | M = 29.36, SD = 8.23 | not indicated | | | X | X | | | | | | | | | | | 2 |
| Sex Trafficking Attitudes Scale (STAS) | Houston-Kolnik et al. (2016) | Self-Report | 601 undergraduate psychology students | | Female (71.4%) Male (28.6%) | X | | | | | | X | | X | X | | X | | | 5 |
| Sexual Narcissism Scale | Widman & McNulty (2010) | Self-Report | 299 undergraduate psychology students (Study 1), 378 male undergraduate psychology students (Study 2) (N=677) | | Study 1: Female (49.2%) Male (50.8%) Study 2: Female (0%) Male (100%) | X | | | | | | | | X | | | | | | 2 |
| Shared Care Instrument (SCI) | Sebern (2005) | Self-Report | 110 patients, 109 family members (N=219) | | N/a | X | | | X | X | | X | | X | | | | | | 5 |
| Shared Care Instrument-Revised (SCI-R) | Sebern (2008) | Self-Report | 223 patients, 220 caregivers (N=443) | | N/a | X | | | | X | X | X | | | X | | | | | 5 |
| Short-Form Five Facet Mindfulness Questionnaire (FFMQ-SF) | Abujaradeh et al. (2020) | Self-Report | 599 high school adolescents | R: 13-19 | Female (49%) Male (51%) | | | | | | | X | X | | | | | X | | 3 |
| Single-Item Measure of Depersonalization | West et al. (2009) | Self-Report | 287 medical students | | Female (54%) Male (46%) | | | | | X | | X | | | | | | | | 2 |
| Single-Item Measure of Emotional Exhaustion | West et al. (2009) | Self-Report | 287 medical students | | Female (54%) Male (46%) | | | | | X | | X | | | | | | | | 2 |
| Single-Item Mindfulness Scale (SIMS) | Meier et al. (2022) | Self-Report | 3125 adult and adolescent participants | study 1: M=18.85(SD=1.18), study 2: M=35.66 (SD=10.05), study 3: M=35.38(SD=13.35), study 4: M=20.50(SD=5.55), study 5: M=18.99 (SD=1.19), study 6: M=15.88(SD=1.15), study 7: M=22.66(SD=4.05), study 8: M=45.36(SD=16.21) | study 1: 108 female(F), 64 male(M); study 2: 124F, 174M; study 3: 199F, 180M, 8 no-binary; study 4: 131F, 69M, 1 non-binary; study 5: 118F, 75M, 1 gender queer; study 6: 400M, 213 F, 31 nonbinary/other; study 7: 88F, 70M; study 8: 543F, 517M | | X | | X | X | X | X | | | | | X | | | 6 |
| Single-Item Self-Compassion Scale (SISC) | Zhang et al. (2022) | Self-Report | study 1:192, study 2: 401, study 3:312, study 4: 386, study 5: 126, study 6: 282, study 9: 110, study 10: 152 | not indicated | not indicated | | X | | | X | - | X | X | | | | | | | 4 |
| Single Item Trait Empathy Scale (SITES) | Konrath et al. (2018) | Self-Report | 3289 adults recruited through MTurk | | Female (52.5%) Male (47.5%) | | X | | | X | X | X | X | | | | | | | 5 |
| Situational Judgment Tests (SJTs) | Goss et al. (2017) | Self-Report | 316 medical students | | N/a | X | | | X | | | | | | | | | | | 2 |
| Social Anhedonia Scale (SAS) | Leak (1991) | Self-Report | 160 undergraduate psychology students (Study 1), 84 undergraduate students (Study 2) (N=244) | | Female (55%), Male (45%) | X | | | | X | X | X | | | | | | | | 4 |

| Instrument | Reference | Study Type | Study Population | Age (range [R] and/or Mean [M] with Standard Deviation [SD]) | Gender | Internal Consistency | Test Re-Test Reliability | Interratter Reliability | Content Validity | Construct Validity | Discriminant/Divergent Validity | Convergent Validity | Predictive Validity | Factor Analysis/Principal Component Analysis | Confirmatory Factor Analysis | Structural Equation Modeling | Control/Correlation with Social Desirability | Other Advanced Statistics | Other Biased Responding or Lie Scale | Total Psychometric Assessments |
|---|---|---|---|---|---|---|---|---|---|---|---|---|---|---|---|---|---|---|---|---|
| Social Emotional Health Survey Higher Education (SEHS-HE) | Furlong et al. (2017) | Self-Report | 1413 undergraduate students | | Female (64%) Male (36%) | X | X | | X | X | | X | X | | X | | | X | | 8 |
| Social-Emotional Responding Task (SERT) | Malti et al. (2021) | Self-Report | sample 1: 291 children; sample 2: 282 children | sample 1: M=6.55, SD=2.02; sample 2: M=6.57, SD=1.56 | sample 1: 50% boys, sample 2: 49% boys | | | X | | X | X | X | | X | X | X | | | | 7 |
| Social Issues Advocacy Scale (SIAS) | Nilsson et al. (2011) | Self-Report | 278 undergraduate and graduate students and professionals (Study 1), 509 undergraduate and graduate students (Study 2) (N=787) | | study 1: Female (78%) Male (22%) study 2: Female (76%) Male (23.2%) Other (0.8%) | X | X | | | X | X | X | | X | | | | X | | 7 |
| Social Justice and Empathy Assessment (SJEA) | Vaffis et al. (2021) | Self-Report | 404 PharmD students | Private university: M = 26.7, SD = 5; Public university: M = 24.7, SD = 3.4 | Private university: 55.5% male; Public university: 59% male | X | X | | X | X | | | X | X | | | | | | 6 |
| Social Relations Survey (SRS) | Lorr et al. (1991) | Self-Report | 409 high school and undergraduate students | | Female (50%) Male (50%) | | | | | X | | | | X | | | | | | 2 |
| Social Self-Compassion Scale (SSCS) | | | | | | X | | | | X | X | X | X | X | X | X | | | | 8 |
| | Rose & Kocovski (2021) | Self-Report | 221 undergraduate participants | M=19.5 | 142 women, 79 men | | | | | X | X | X | | X | X | | | | | 5 |
| | Rose & Kocovski (2020) | Self-Report | 221 adults (Sample 1), 227 adults (Sample 2), 271 adults (Sample 3) (N=719) | | Female (72.5%) Male (27.5%) | X | | | | X | X | X | X | X | X | X | | | | 8 |
| Social Skills Improvement System (SSIS) | Panayiotou et al. (2022) | Self-Report | 3331 children | 8-10 | male; n = 1,720, 51.6% | | | | | | X | | | X | X | | | | | 3 |
| Spatial Perspective Task (SPT) | Chiu & Yeh (2018) | Self-Report | 158 undergraduate students | | Female (57%) Male (43%) | | | | | | | X | | | | | | | | 1 |
| Spiritual Modeling Inventory of Life Environments (SMILE) | Oman et al. (2009) | Self-Report | 1010 undergraduate students | | Female (71.5%) Male (28.5%) | X | X | | | X | X | X | | | | | X | | | 6 |
| Sports Injury Clinic Athlete Satisfaction Scale (SICASS) | Taylor & May (1995) | Self-Report | 160 patients from five sports injury clinics in the UK | | Female (37%) Male (63%) | X | | | | X | | | | | | | | | | 2 |
| Staff Attitudes Towards Patients who Attempt Suicide [Unnamed] | Aish et al. (2002) | Self-Report | 1010 medical staff members | | Female (71.8%) Male (28.2%) | | | | | | | | | | X | X | | | | 2 |
| Standardized Assessment of Severity of Personality Disorder (SASPD) | Waugh et al. (2021) | Self-Report | 25 grdauate students and PhD-Level psychologists | M = 29.36, SD = 8.23 | not indicated | | | X | X | | | | | | | | | | | 2 |
| State-Empathic Concern Scale | Johnson et al. (2019) | Self-Report | 199 counseling students | R: 22-69, M = 32.04 | Female (81.4%) Male (18.6%) | X | | | X | X | X | X | | | X | | | | | 6 |
| State Empathy Scale | Shen (2010) | Self-Report | 289 undergraduate students in speech communication classes (Study 1), 189 adults (Study 2) (N=478) | | N/A | X | | | | X | X | X | | | | | | | | 4 |
| State-Interpersonal Reactivity Index (S-IRI) | Johnson et al. (2021) | Self-Report | 240 mental health professionals and graduate-level trainees | M=31.18, SD=10.45 | 75.4% female, 8.8% male, 1.3% other | X | | | X | X | X | X | | X | X | | | | | 7 |
| State Moral Elevation Scale (SMES) | McGuire et al. (2022) | Self-Report | 930 adult participants | M=39.27, SD=11.98 | 55.38% male, 44.52% female | X | | | X | X | X | X | | X | X | X | | | | 8 |
| State Self-Compassion Scale-Long (SSCS-L) | Neff et al. (2021) | Self-Report | study 1: 588 participants; study 2: 411 participants; study 3: 139 participants | study 1: M=35.2, SD 10.1; study 2: M=20.6, SD=1.96; study 3: M=20.49, SD=1.81 | study 1: 58% male, 40.6% female; study 2: 31.1% male, 67.2% female; study 3: 36.7% male, 62.6% female | X | | | | | | | X | X | X | X | | | | 5 |
| State Self-Compassion Scale-Short Form (SSCS-S) | Neff et al. (2021) | Self-Report | study 1: 588 participants; study 2: 411 participants; study 3: 139 participants | study 1: M=35.2, SD 10.1; study 2: M=20.6, SD=1.96; study 3: M=20.49, SD=1.81 | study 1: 58% male, 40.6% female; study 2: 31.1% male, 67.2% female; study 3: 36.7% male, 62.6% female | X | | | | | | | X | X | X | X | | | | 5 |
| State-Trait Scales for Distinct Positive Emotions (STS-DPE) | Weidman & Tracy (2020) | Self-Report | study 1 = 150 participants; study 2 = 1372 participants; study 3 = 1962 participants | study 1: M = 20.4, SD= 2.47; study 3: M = 22.25 | study 1: 85% women; study 2: 72% women; 77% women | | | | X | X | X | X | | X | | | | | | 5 |
| Suicidal Patients Scale (USP Scale) | Aish et al. (2002) | Self-Report | 1010 medical staff members | | Female (71.8%) Male (28.2%) | | | | | | | | | | X | X | | | | 2 |
| Suicide Attitude Vignette Experience (SAVE) Scale | Stillion et al. (1984) | Self-Report | 198 high school students | M = 18.2 | Female (48%) Male (52%) | | X | | | | | X | | X | | | | | | 3 |
| Sulliman Scale of Social Interest (SSSI) | Watkins & St. John (1994) | Self-Report | 207 undergraduate students | | N/a | X | | | X | | | | | | | | | | | 2 |
| Sussex-Oxford Compassion for Others Scale (SOCS-O) | Gu et al. (2020) | Self-Report | | | Female (79.6%) Male (20.4%) | X | | | X | X | X | X | | | X | | | | | 6 |
| Sussex-Oxford Compassion for the Self Scale (SOCS-S) | | Self-Report | | | | | | | | | | | | | | | | | | 0 |
| Systemizing Quotient - Child (SQ-C) | Auyeung et al. (2009) | Self-Report | 1256 parents of typically developing children | parents of typically developing children | N/a | X | X | | | | | | | | | | | | | 2 |
| Test of Accurate Perception of Patients' Affect (TAPPA) | Hall et al. (2014) | Self-Report | 524 undergraduate students, nursing students, medical students, and internal medicine residents | M = 24.25 | Female (61.7%) Male (38.3%) | X | X | | | X | X | | | | | | | | | 4 |
| Test of Self-Conscious Affect - Adolescent (TOSCA-A) | Watson et al. (2016) | Self-Report | 931 children and adolescents | | Female (73.8%) Male (26.2%) | X | | | X | | | X | | | X | | | | | 4 |
| Therapeutic Environment Scales (TESS) | Veale et al. (2016) | Self-Report | 81 British patients in treatment for anxiety disorders | | Female (68%) Male (32%) | X | X | | | | | X | | | | | | | | 3 |
| Thought Impact Scale (TIS) | Palsson (2020) | Self-Report | 216 university staff, students, and faculty | | Female (81.9%) Male (28.1%) | X | X | | | X | | X | | | | | | X | | 5 |
| Tool to Assess Inpatient Satisfaction with Care from Hospitalists (TAISCH) | Torok et al. (2014) | Self-Report | 203 hospitalized patients | M = 60 | Female (56%) Male (44%) | | | | | | | | | X | X | | | | | 2 |
| Toronto Empathy Questionnaire (TEQ) | Spreng et al. (2009) | Self-Report | 200 Canadian female undergraduate students (Study 1), 79 Canadian undergraduate students (Study 2) (N=279) | Study 1: M = 18.8 years, SD = 1.2 Study 2: M = 18.9 years, SD = 3.0 | Study 1: Female (50%) Male (50%) Study 2: Female (69.6%) Male (30.4%) | X | X | | | X | X | X | | X | | | | X | | 7 |
| Trait Emotional Intelligence Questionnaire Short Form (TEIQue-SF) | Snowden et al. (2015) | Self-Report | 938 nursing and computing students | | Female (83.7%) Male (15.9%) Other (.4%) | | | | | X | | | | X | X | | | X | | 4 |
| Trait Sympathy Scales (TSS) | Lee (2009) | Self-Report | 732 undergraduate and graduate students (Study 1), 272 undergraduate students (Study 2), 77 undergraduate students (Study 3) (N=1081) | | Study 1: Female (69.4%) Male (30.3%) Study 2: Female (76.6% female) Male (23.4%) | X | | | X | X | X | X | X | X | X | | X | | | 9 |
| Transactional Analysis Ego States | Williams et al. (1983) | Self-Report | 105 undergraduate students (Study 1), 80 undergraduate students (Study 2), 68 undergraduate students (Study 3) (N=253) | | Study 1:F= Female 37.1, Male 62.9 Study 2: Female 50%, Male 50% Study 3: Female 45.6%, Male 54.4% | | | | X | | | | | X | | | | | | 2 |
| Transpersonal Gratitude Scale | Elfers et al. (2014) | Self-Report | 314 adults (Study 1), 385 adults (Study 2) (N=699) | | Study 1: Female (71.3%) Male (28.7%) Study 2: Female (57.1%) Male (42.9%) | X | | | X | | | | | X | X | | | | | 4 |
| Trauma-Informed Self-Care Measure | Salloum et al. (2018) | Self-Report | 177 child welfare workers | | Female (87%) Male (11.9%) Other (0.6%) | X | | | | X | | | | X | X | | | | | 4 |
| Trauma-Related Shame Inventory - Short Form (TRSI-SF) | Grau et al. (2022) | Self-Report | sample 1: 228 patients who voluntarily sought treatement, sample 2: 199 | sample 1: M=35.9, SD=12.5; sample 2: M=34.78, SD=11.6 | sample 1: 86% female, sample 2: 85% female | X | | | | X | X | X | | X | X | | | | | 6 |
| Twelve Step Facilitation Adherence Competence Empathy Scale (TSF ACES) | Campbell et al. (2013) | Self-Report | 471 outpatients diagnosed with a substance use disorder | older than 18 | Female (59%) Male (41%) | X | | | X | | | X | | | | | | | | 3 |
| Unconditional Self-Kindness Scale | Smith et al. (2018) | Self-Report | 1452 undergraduate students | 18+ | Female (68.8%) Male (31%) | X | X | | | X | X | X | | X | X | | | | | 7 |
| Undergraduate Nursing Palliative Care Knowledge Survey (UNPCKS) | Davis et al. (2020) | Self-Report | Phase II: 20 Phase IV: 262 | phase ii: NA phase iv: M=23.6 | phase ii: NA phase iv: female (88.5%), male (11.4), other (0.40) | X | | | | | | | | X | | | | | | 2 |
| Universal Worth Scale (UWS) | Rouse (2012) | Self-Report | 345 adults (Study 1), 345 adults (Study 2), 133 undergraduate students (Study 3) (N=823) | | | | X | | | X | | X | | | X | | X | | | 5 |
| Verona Coding Definitions of Emotional Sequences (VR-CoDES) | Del Piccolo et al. (2011) | Self-Report | 104 healthcare providers | | Female (42.5%) Male (37.5%) | | X | | | | | | | X | | | | | | 2 |

| Instrument | Reference | Study Type | Study Population | Age (range [R] and/or Mean [M] with Standard Deviation [SD]) | Gender | Internal Consistency | Test Re-Test Reliability | Interratter Reliability | Content Validity | Construct Validity | Discriminant/Divergent Validity | Convergent Validity | Predictive Validity | Factor Analysis/Principal Component [illegible] | Confirmatory Factor Analysis | Structural Equation Modeling | Control/Correlation with Social Desirability | Other Advanced Statistics | Other Biased Responding or Lie Scale | Total Psychometric Assessments |
|---|---|---|---|---|---|---|---|---|---|---|---|---|---|---|---|---|---|---|---|---|
| Vicarious Posttraumatic Growth Inventory (VPTGI) | Deaton & Ohrt (2023) | Self-Report | 651 helping professionals and helping professionals-in-training working with clients impacted by trauma | M=39.6, SD=10.95 | 90.2% women | X | | | | X | X | X | | X | | X | | | | 6 |
| Vicarious Resilience Scale (VRS) | Killian et al. (2017) | Self-Report | 190 helping professionals working with severe trauma | M = 44.43, SD = 12.5 | Female (72%) Male (28%) | X | | | | | | | | X | | | | | | 2 |
| Vicarious Trauma Organizational Readiness Guide (VT-ORG) | Hallinan et al. (2019) | Self-Report | 3018 employees of first responder agencies and victim assistance agencies | | Female (28.9%) Male (65.1%) Other (6%) | X | | | | | | | | | X | X | | X | | 4 |
| Vicarious Trauma Scale (VTS) | Aparicio et al. (2013) | Self-Report | 157 social workers | | | X | | | | | | | | X | X | | | X | | 4 |
| Victim Concern Scale (VCS) | Clements et al. (2006) | Self-Report | 219 undergraduate students | R: 18-36 | Female (54%) Male (46%) | X | | | X | | | | | X | | | | | | 3 |
| Victim Empathy Response Assessment (VERA) | Terry et al. (2009) | Self-Report | 103 adult males | R: 16-70, M = 33.71, SD = 15.25 | Female (0%) Male (100%) | | | | | X | | | | X | | | | | | 2 |
| Visual CARE Measure | Murphy et al. (2013) | Self-Report | 99 patients attending routine appointments in a GP practice | | N/a | X | | | | | | | | | | | | | | 1 |
| Working Alliance Inventory - General Pracitce (WAI-GP) | Hunik et al. (2021) | Self-Report | 146 patients | 52.1% were aged older than 45 years | 62.3% female | X | | | | X | | X | | X | | | | | | 4 |
| Workplace Compassion Scale | Mandliya & Pandey (2023) | Self-Report | 406 adults | 18-60; average age was 30.91 years (SD = 7.3) | 198 (48.8%) were male, 207 (51%) were females | X | | | X | | X | X | X | X | X | | | | | 7 |
| Young Children's Empathy Measure (YCEM) | Poresky (1990) | Self-Report | 38 children | | Female (55%) Male (45%) | X | | X | | X | X | X | | | | | | | | 5 |
| Youth Suicide Scale (YSS) | Range et al. (1987) | Self-Report | 191 undergraduate psychology students (Sample 1), 152 undergraduate psychology students (Sample 2) (N=343) | | N/a | | X | | | | | | | X | | | | | | 2 |

**S1 Table. Measure populations and psychometric assessments.** S1 Table includes all compassion and empathy measures identified categorized by type (behavior/expert coder, peer/corollary report, self-report), then sorted alphabetically by measure name, and for measures with mroe than one articles, from newest to oldest.
