# Supplementary material for: Measures of empathy and compassion: A scoping review
Source: PLoS One. 2024 Jan 19;19(1):e0297099. doi: 10.1371/journal.pone.0297099 (PMC10798632; doi:10.1371/journal.pone.0297099)
Supplement: S2 Table — (PDF) [file pone.0297099.s002.pdf]

Instrument	Internal Consistency	Test Re-Test Reliability	Interratter Reliability	Content Validity	Construct Validity	Discriminant/Divergent Validity	Convergent Validity	Predictive Validity	Factor Analysis/ Principal Component Analysis	Confirmatory Factor Analysis	Structural Equation Modeling	Control/ Correlation with Social Desirability	Other Advanced Statistics	Other Biased Responding or Lie Scale	Total Psychometric Assessments
Interpersonal Reactivity Index (IRI)	X	X	N/A	X	X	X	X	X	X	X	X	X	X		12
Self-Compassion Scale (SCS)	X	X	N/A	X	X	X	X	X	X	X	X	X	X		12
Body Compassion Questionnaire (BCQ)	X	X	X	X	X	X	X		X	X	X				10
Compassion Scale (CS)	X	X	N/A	X	X	X	X		X	X	X	X			10
Complementary Measure of Psychotherapy Measure (COMPO)	X	X	N/A	X	X	X	X	X	X	X	X				10
Empathy Quotient (EQ)	X	X	N/A		X	X	X	X	X	X		X	X		10
Four-Item Mentalising Index	X	X	N/A	X	X	X	X	X	X	X	X				10
Genuine Happiness Scale (GHS)	X	X	N/A	X	X	X	X	X	X	X	X				10
Jefferson Scale of Physician Empathy (JSPE)	X	X	N/A	X	X	X	X		X	X	X		X		10
Moral Foundations Questionnaire (MFQ)	X	X	N/A	X	X	X	X	X	X	X	X				10
Prejudice Confrontation Styles (PCS) Scale	X	X	N/A	X	X	X	X	X	X	X	X				10
Questionnaire of Cognitive and Affective Empathy (QCAE)	X		X		X	X	X	X	X	X	X		X		10
18-item short form Coping with Children's Negative Emotions Scale (CCNES)	X	X	N/A	X	X		X		X	X	X		X		9
6-item brief short form Coping with Children's Negative Emotions Scale (CCNES)	X	X	N/A	X	X		X		X	X	X		X		9
Arble Estimate of Selfobject Pursuits (AESOP)	X		N/A	X	X	X	X		X	X		X	X		9
Attitudes Related to Trauma-Informed Care Scale (ARTIC)	X	X	N/A	X	X	X	X	X	X	X					9
Bolton Compassion Strengths Indicators (BCSIs)	X	X	N/A	X	X	X	X	X	X	X					9
Child-Related Values Survey (CRVS)	X		N/A		X	X	X		X	X	X	X		X	9
Compassionate Love Scale for Humanity - Short Form (CLS-H-SF)	X		N/A	X	X	X	X		X	X		X	X		9
Defending Behaviors Scale (DBS)	X	X	N/A	X	X	X	X	X	X		X				9
ECRC Altruism Scale	X		N/A	X	X	X	X	X	X	X	X				9
Empathetic Care Scale (ECS)		X	N/A		X	X	X	X	X	X		X	X		9
Empathic Experience Scale (EES)	X		N/A	X	X	X	X		X	X	X	X			9
Essential Properties of Yoga Questionnaire (EPYQ)	X	X	X	X	X	X	X		X	X					9
Fat Attitudes Assessment Toolkit (FAAT)	X	X	N/A	X	X	X	X		X	X		X			9
Fears of Compassion in Sport Scale (FCSS)	X	X	N/A	X	X	X	X	X	X	X					9
Measure of Relational Climate [Unnamed]	X		N/A	X	X	X	X		X	X	X			X	9
Mindful Attention Awareness Scale (MAAS)	X	X	N/A		X	X	X		X	X	X		X		9
Multiple Mini-Interview (MMI)	X		X	X	X	X	X	X	X				X		9
Observed Mindfulness Measure (OMM)	X	X	X	X	X	X	X		X	X					9
Prosocial and Antisocial Behavior in Sport Scale (PABSS)	X	X	N/A	X	X	X	X		X	X			X		9
Reading the Mind in the Eyes Test (RMET)	X		X		X	X	X	X	X	X	X				9
Self-Compassion Scale - Short Form (SCS-SF)	X	X	N/A		X	X	X		X	X	X		X		9
Trait Sympathy Scales (TSS)	X		N/A	X	X	X	X	X	X	X		X			9
15-item Mutualism Scale	X	X	N/A		X	X		X	X	X	X				8
Accountability Scale	X	X	N/A	X	X	X	X		X	X					8
Active Empathetic Listening (AEL) Scale	X		N/A		X	X	X		X	X	X		X		8
Awareness, Knowledge, Skills - General (ASK-G)	X		N/A	X	X	X	X		X	X	X				8
Barrett-Lennard Relationship Inventory Observer Form (BLRI Obs-40)	X	X	X		X		X		X	X	X				8
Body Compassion Scale (BCS)	X		N/A	X	X		X		X	X		X	X		8
Consultation and Relational Empathy (CARE) Measure	X		X	X	X		X		X	X			X		8
Contingency Management Competence Scale (CMCS)	X		X	X	X	X	X	X	X						8
Counselor Behavior Analysis (CBA)	X		X	X	X	X	X	X		X					8
Emotional Skills and Competence Questionnaire (ESCQ)	X		N/A	X	X	X	X	X	X	X					8
Empathic Counselor Response Scale (ECRS)	X		X	X	X	X	X		X	X					8
Empathy for Pain Scale (EPS)	X		N/A	X	X	X	X	X	X	X					8
Equanimity Barriers Scale (EBS)	X		X	X	X	X	X		X	X					8
Facets of Emotional Experiences in Everyday Life Scale (FEEELS)	X		N/A	X	X	X	X		X	X	X				8
Griffith Empathy Measure (GEM)	X	X	X		X	X	X		X				X		8
Hogan Empathy Scale (HES)	X	X	X		X	X	X	X	X						8
Interpersonal Regulation Questionnaire (IRQ)		X	N/A		X	X	X	X	X	X		X			8
Level of Personality Functioning Scale - Self Report (LPFS-SR)	X	X	X	X	X	X	X		X						8
Measure of Expressed Empathy	X		N/A	X	X		X	X	X	X	X				8
Millennial Organizational Citizenship Behavior (OCB) Scale	X		N/A	X	X	X	X		X	X	X				8
Moral Inclusion/Exclusion of Other Groups Scale (MIEG)	X		N/A	X	X	X	X	X	X	X					8
Nursing Shared Decision-Making Attitude Scale (NSDMA)	X		N/A	X	X	X	X		X	X	X				8
Physician-Caregiver Relationship Scales (PCRS)	X		X	X	X	X		X		X			X		8
Prejudice toward People with Depression (PPD)	X		N/A	X	X	X	X		X	X	X				8
Prejudice toward People with Schizophrenia (PPS)	X		N/A	X	X	X	X		X	X	X				8
Psychosocial Costs of Racism to Whites Scale (PCRW)	X	X	N/A		X	X	X		X	X		X			8
Relational Care Scale (RCS)	X	X	X	X	X	X	X		X						8
Relational Engagement of the Sacred for a Transgression (REST) Scale	X		N/A		X			X	X	X	X	X	X		8
School Support Scale	X		N/A	X	X	X	X	X	X	X					8
Schwartz Center Compassionate Care Scale (SCCCS)	X	X	N/A	X	X		X		X	X			X		8
Sinclair Compassion Questionnaire (SCQ)	X	X	N/A	X	X	X	X		X	X					8

Instrument	Internal Consistency	Test Re-Test Reliability	Interratter Reliability	Content Validity	Construct Validity	Discriminant/Divergent Validity	Convergent Validity	Predictive Validity	Factor Analysis/ Principal Component Analysis	Confirmatory Factor Analysis	Structural Equation Modeling	Control/ Correlation with Social Desirability	Other Advanced Statistics	Other Biased Responding or Lie Scale	Total Psychometric Assessments
Social Emotional Health Survey Higher Education (SEHS-HE)	X	X	N/A	X	X		X	X		X			X		8
Social Self-Compassion Scale (SSCS)	X		N/A		X	X	X	X	X	X	X				8
State Moral Elevation Scale (SMES)	X		N/A	X	X	X	X		X	X	X				8

S2 Table. Measures with 8+ psychometric assessments. S2 Table shows compassion and empathy measures that have more than 8 types of psychometric assessments reported, sorted by measures with the most types of psychometric assessments completed to the least.
