# Supplementary material for: Measures of empathy and compassion: A scoping review
Source: PLoS One. 2024 Jan 19;19(1):e0297099. doi: 10.1371/journal.pone.0297099 (PMC10798632; doi:10.1371/journal.pone.0297099)
Supplement: S3 Table — (PDF) [file pone.0297099.s003.pdf]

Instrument	Published Year	Type of Measure	Subscales/ Forms	Total Number of Items	Sample Item(s)	Response set
(Inner) Strength-Based Inventory (SBI)	2020	Self-Report	(generosity) (morality) (mindfulness/meditation) (wisdom) (perseverance) (patience and endurance) (truthfulness) (determination) (loving-kindness and equanimity)	10	"I always feel sympathetic for everyone even if I do not like him or her." "Sometimes I react with physical violence."	Each item has its own rating ranging from five to eight choices
1-Item Brief Burnout Screening [Unnamed]	2009	Self-Report	No Subscales	1		
10-item Remembered Relationship with Parents (RRP10) Scale	1958	Self-Report	(alienation father) (alienation mother) (control father) (control mother)	10	"Often felt that my mother did not understand me" "Wished mother would worry less about me"	five-point Likert scale from 0 (false) to 4 (true)
15-item Mutualism Scale	2022	Self-Report	(Partner Choice) (Proportional Sharing) (Proportioonal Punishment) (Proportional Helping)	15	“I hold the door open for others” “I help when it helps others much more than it costs me, like holding a door open”	Likert response scale coded from 1 (strongly disagree) to 5 (strongly agree)
18-item short form Coping with Children’s Negative Emotions Scale (CCNES)	2002	Self-Report	(emotion coaching) (emotion dismissing)	18	"Acknowledge that it can be disappointing to miss out on something you want to do" "Acknowledge that it can feel worrying being separated from you"	7-point Likert-type scale (1 = very unlikely, 4 = medium, 7 = very unlikely)
2-Item Brief Burnout Screening [Unnamed]	2009	Self-Report	(emotional exhaustion) (depersonalization) (physician work-life study)	2		
20-Item Toronto Alexithymia Scale (TAS-20)	1994	Self-Report	(Difficulty identifying feelings (DIF)) (Difficulty describing feelings (DDF)) (Externally oriented thinking (EOT))	20	"I am often confused about what emotion I am feeling" "I prefer talking to people about their daily activities rather than their feelings"	5-point Likert scale (1 = strongly disagree, 5 = strongly agree)
24-Item Rating Form of Clinical Competence [Unnamed]	1986	Peer/Corollary Report	(knowledge and clinical capabilities) (professionalism)	24		
30-Item Rating Scale of Parent Satisfaction with Pediatric Neuropsychological Evaluations [Unnamed]	1979	Self-Report	(general satisfaction) (clinician acceptance/empathy) (provision of help) (facilities/administrative assistance)	30	"Made me feel less stressed" "Provided a caring atmosphere"	5-point Likert type scale from 1 (strongly disagree) to 5 (strongly agree)
5-item Compassion Measure	2019	Peer/Corollary Report	No Subscales	5	"During this emergency department visit, how often do you feel your clinician cared about your emotional or psychological well-being?" "During this emergency department visit, how often do you feel your clinician was interested in you as a whole person?"	4-point Likert-type scale ranging from 1 (never) to 4 (always)
6-item brief short form Coping with Children’s Negative Emotions Scale (CCNES)	2002	Self-Report	(emotion coaching) (emotion dismissing)	6	"Acknowledge that it can feel worrying being separated from you" "encourage my child to talk about his/her nervous feelings of embarrassment (expressive encouragement)."	7-point Likert-type scale (1 = very unlikely, 4 = medium, 7 = very unlikely)
Accountability Scale	2022	Self-Report	No Subscales	11	"I usually welcome being accountable to others" "I am willing to be held responsible for my contributions on tasks"	1 = disagree strongly; 2 = disagree somewhat; 3 = neither agree nor disagree; 4 = agree somewhat; 5 = agree strongly
Actions and Feelings Questionnaire (AFQ)	2015	Self-Report	(production) (perception)	18	"I tend to pick up on people’s body language" "I wouldn't tend to know what someone was feeling like if they did not say"	4-point Likert-type scale ranging from 0 (strongly disagree) to 3 (strongly agree)
Active Empathetic Listening (AEL) Scale	2006	Self-Report	(sensing) (processing) (responding)	45	"I understand how others feel." "I assure others that I will remember what they say."	7-point Likert-type scale ranging from 1 (never or almost never true) to 7 (always or almost always true)
Ad Response Empathy (ARE)	2003	Self-Report	(response sympathy) (response empathy)	10	"While watching the ad, I experienced feeling as if the events were really happening to me." "While watching the ad, I felt as though I were one of the characters."	7-point Likert-type scale ranging from 1 to 7
Ad Response Sympathy (ARS)	2003	Self-Report	(response sympathy) (response empathy)	10	"Based on what was happening in the commercial, I understood what the characters were feeling" "Based on what was happening in the commercial, I understood what was bothering the characters."	7-point Likert-type scale ranging from 1 to 7
Adapted Health Communication Assessment Tool© (adapted HCAT©)	2013	Peer/Corollary Report	(relationship building) (empowering) (empathy response)	9	"Used empathy to build relationship with patient/family" "Inquired about patient's/family's feelings regarding birth, illness, injury and/or treatment"	Yes, No, Unsure
Adapted SERVQUAL	2020	Peer/Corollary Report	(tangibles) (reliability) (responsiveness) (assurance) (empathy) (sanitation and hygiene)	30	"Employees in the hospital are always willing to help you." "Employees in the hospital are never too busy to respond to your request."	7-point Likert scale with descriptive anchors ranging from strongly disagree (1) to strongly agree (7)
Adolescent and Adult Mindfulness Scale (AAMS)	2018	Self-Report	(attention and awareness) (being non-reactive) (being non-judgmental) (being self-acceptant)	19	"I tell myself that I shouldn't be feeling the way I'm feeling" "I intentionally stay aware of my feelings."	5-point Likert-type scale ranging from 1 (never true) to 7 (always true)

Instrument	Published Year	Type of Measure	Subscales/ Forms	Total Number of Items	Sample Item(s)	Response set
Adolescent Attitudes towards Dementia Scale (A-ADS)	2017	Self-Report	(perceptions of dementia) (personal sacrifice) (empathy with people living with dementia)	23	"I would be annoyed if my parents asked me to spend time with a family friend who has dementia rather than see my friends" "I would donate my time or money to help people with dementia"	7-point Likert-type scale ranging from 1 (strongly disagree) to 7 (strongly agree)
Adult-Adolescent Parenting Inventory (AAPI)	1984	Self-Report	(inappropriate expectations) (lack of empathy) (parental value of corporal punishment) (parent-child role reversal)	32	"Children should be able to feed themselves by twelve months" "Children will quit crying faster if they are ignored"	5-point Likert-type scale ranging from 1 (strongly agree) to 7 (strongly disagree)
Affective and Cognitive Measure of Empathy (ACME)	2016	Self-Report	(cognitive empathy) (affective resonance) (affective dissonance)	45	"I have a hard time reading people's emotions" "I can usually guess what's making someone angry"	5-point Likert-type scale ranging from 1 (disagree strongly) to 5 (agree strongly)
Affective Forecasting Situation Questionnaire	2014	Self-Report	No Subscales	16	"I feel more sympathetic toward people who get AIDS from blood transfusions than those who get it from IV drug abuse" "Most people who have AIDS have only themselves to blame"	7-point Likert-type scale ranging from 0 (not at all) to 6 (very strong)
Affective Sensitivity Scale [Unnamed]	1963	Behavioural/Expert Coder	No Subscales	89		
AIDS Attitude Scale	1992	Self-Report	(avoidance) (empathy	21	"I would rather work with a better class of people than AIDS people." "I sometimes find it hard to be sympathetic to AIDS patients."	6-point Likert-type scale ranging from 1 (strongly disagree) to 6 (strongly agree)
AIDS Attitude Scale-Generic (AAS-G)	1992	Self-Report	(avoidance) (empathy	21	"I would rather work with a better class of people than AIDS people." "I sometimes find it hard to be sympathetic to AIDS patients."	6-point Likert-type scale ranging from 1 (strongly disagree) to 6 (strongly agree)
Altruism Scale	2015	Peer/Corollary Report	(altruism) (solidarity)	22		
Altruism Scale for Children (ASC)	2020	Self-Report	No Subscales	30	A teacher is walking into the building and drops some things as she attempts to open the door. I: (a) ask my friends if we should offer help, (b) stop and offer to help, or (c) keep walking by."One response reflects an altruistic response (scored with three points), one response focuses on a self-centered response (scored with one point), and the third option ranges in the middle (scored with two points) relating to a reliance on feedback from others in the decision-making process	
Analog Task of Prosocial Helping (AToP-H)	2022	Behavioural/Expert Coder	(Instrumental helping) (Informative helping) (Empathic helping)	9	The examiner provided a graduated series of cues to elicit helping. Cues were delivered in a consistent sequence for each item, from subtlest to most explicit.	The examiner provided cues every 4 s until the child provided a response that was consistent with that item's context and helping type
Anasakti Scale [Unnamed]	1992	Self-Report	(outcome vulnerability) (faith in god) (empathy) (frustration tolerance) (effort orientation and emotional equipoise)	22	"I feel remembering God is the perfect solution to all the problems." "I deeply care for people who are suffering and offer my comfort."	5-point Likert-type scale ranging from 1 (never) to 5 (always)
Appreciative Joy Scale (AJS)	2017	Self-Report	(sense of joy) (positive interpersonal bias) (self-transcendence)	14	"I can usually notice many little kind acts performed by my friends" "I would be sincerely happy for my friends' achievements."	9-point Likert-type scale ranging from 1 (not at all like me) to 9 (totally like me)
Arab Muslim Perceptions of Perinatal Loss Care (AMPPLC)	2021	Self-Report	No Subscales	29	"After delivery, I would want to hold my baby who died" "After delivery, I would like an opportunity to bathe my baby who died."	4-point LIkert-type scale ranging from strongly agree (4) to strongly disagree (1)
Arble Estimate of Selfobject Pursuits (AESOP)	2017	Self-Report	(idealizing) (mirroring) (twinship)	31	"I feel that people do not appreciate the struggles I've had to face" "It helps me to be around people going through the same things that I am going through"	7-point Likert-type scale ranging from 1 (not at all true of me) to 7 (very true of me)
ASP ["Expressions of Spirituality"] Questionnaire	2007	Self-Report	(prayer, trust in god, and shelter) (insight, awareness, and wisdom) (transcendence conviction) (compassion, generosity, and patience) (conscious interactions) (gratitude, reverence and respect) (equanimity.)	40		
ASPIRE Coaching Fidelity Checklist (ACFC)	2020	Behavioural/Expert Coder	(session structure) (session process) (set goals and monitor progress) (assess and personalize self-regulatory content) (manage the session) (creates a supportive and empathetic climate) (stays on track)	192	"Organized and prepared" "Delivers didactic material in a matter of fact and friendly way"	three-point ordinal scale (0 = did not cover/demonstrate; 1 = partially covered/demonstrated; 2 = fully covered/demonstrated)
Assessment of Motivational Interviewing Groups – Observer Scales (AMIGOS)	2013	Behavioural/Expert Coder	(group processes) (client-centered style) (mi change focus)	18		

Instrument	Published Year	Type of Measure	Subscales/ Forms	Total Number of Items	Sample Item(s)	Response set
Attitudes About Reality Scale (AAR)	1986	Self-Report	(individual determinism) (societal determinism) (variable determinism)	40	"Effort can often make up for a lack of talent in an area." "The mother-infant relationhip a key to understanding later adult development" "Most people would cooperate with each other if only they understood that everyone would benefit from such behavior"	7-point Likert-type scale ranging from 1 (disagree almost completely) to 7 (agree almost completely)
Attitudes Related to Trauma-Informed Care Scale (ARTIC)	2016	Self-Report	(underlying causes of problem behavior and symptoms) (responses to problem behavior and symptoms) (empathy and control) (self-efficacy at work) (reactions to the work) (personal support of TIC) (system-wide support for TIC)	45	"If students don't apologize to me after they act out, I look like a fool in front of others" "I realize that students may not be able to apologize to me after they act out"	7-point bipolar Likert scale containing a TIC-favorable and a TIC-unfavorable statement
Attitudes toward Suicidal Patients	1998	Self-Report	(emotional attitudes) (empathic or rejecting) (attitudes toward further training) (attitudes toward the need for psychiatric care)	100	"A patient who has tried to commit suicide usually takes up a great deal of staff time" "Once people have made up their minds to commit suicide, you cannot stop them"	
Attitudes Toward Women with HIV/AIDS Scale	1995	Self-Report	(attitudes toward women with hiv/aids) (female-specific knowledge) (attitudes toward female sexuality)	66	“Pregnant women with HIV/AIDS should be forced to have their baby tested for HIV” "Women with HIV/AIDS are unfit mothers."	5-point Likert-type scale ranging from 1 (strongly agree) to 5 (strongly disagree)
Attribution Questionnaire-Substance Use Disorder (AQ-SUD)	2021	Self-Report	(negative emotions) (assessment of responsibility) (lack of empathy) (social disengagement)	18	"I would feel pity for Derek." "How much sympathy would you feel for Derek?"	9-point Likert scale from 1 (not at all) to 9 (very likely)
Augmented Stanford Faculty Development Program Questionnaire (SFDPQ)	2010	Peer/Corollary Report	No Subscales	58		
Awareness, Knowledge, Skills - General (ASK-G)	2022	Self-Report	(Knowledge) (Awareness of others) (Proactive skills development) (Awareness of self)	37	"When I make a cultural misstep, I see that moment as a learning opportunity." "When I say something that is offensive to another person, I can apologize even if I do not fully understand how I have offended them."	6-point Likert-type scale: (1) strongly agree, (2) disagree, (3) slightly disagree, (4) slightly agree, (5) agree, (6) strongly agree
Barrett-Lennard Relationship Inventory - Empathy (BLRI-E)	1962	Self-Report	(empathy) (regard) (congruence)	16	"S/he feels deep affection for me" "I feel that she really values me"	6-point Likert-type scale ranging from 1 (I strongly feel that is untrue) to 6 (I strongly feel that is true)
Barrett-Lennard Relationship Inventory (BLRI)	1962	Self-Report	(level of regard) (advice-giving) (empathy) (unconditionality of regard) (congruence)	64	"S/he feels deep affection for me" "I feel that she really values me"	6-point Likert-type scale ranging from 1 (I strongly feel that is untrue) to 6 (I strongly feel that is true)
Barrett-Lennard Relationship Inventory for Receiver (B-L RI: OS-64)	1962	Self-Report	(Level of regard) (Empathic understanding) (Congruence) (Unconditionality of regard)	64	"____ respects me as a person" "______ wants to understand how I see things"	six-point Likert scale (-3: NO, I strongly feel that it is not true; -2: No, I feel it is not true; -1: (No) I feel that it is probably untrue, or more untrue than true; +1: (Yes) I feel that it is probably true, or more true than untrue; +2: Yes, I feel it is true; +3: YES, I strongly feel that it is true
Barrett-Lennard Relationship Inventory Observer Form (BLRI Obs-40)	2015	Behavioural/Expert Coder	(Empathic Understanding) (Level of Regard) (Unconditionality of Regard) (Congruence)	40	"S/he evidently feels a responsive personal warmth and liking for B" "S/he senses or realizes how B is feeling"	6-point Likert-type scale
Barriers to Physician Compassion Questionnaire	2014	Self-Report	No Subscales	34	"Feeling burned out" "Having a limited time for consultations"	7-point Likert-type scale ranging from 1 (minimal) to 7 (a great deal)
Basic Empathy Scale (BES)	2004	Self-Report	(affective empathy) (cognitive empathy)	40	“My friend’s emotions don’t affect me much” “I can understand my friend’s happiness when she/he does well at something”	5-point Likert-type scale ranging from 1 (strongly disagree) to 5 (strongly agree)
Beech Brook Therapy Assessment Instrument	2002	Peer/Corollary Report	No Subscales	9		
Behavior Change Counseling Index (BECCI)	2005	Peer/Corollary Report	No Subscales	11	"Practitioner invites the patient to talk about behaviour change" "Practitioner demonstrates sensitivity to talking about other issues"	0. Not at all;1. Minimally; 2. To some extent; 3. A good deal; 4. A great extent
Behavioral Assessment of Empathy	1968	Behavioural/Expert Coder	(communication of acceptance) (allowing the child self-direction) (involvement)	15		
Body Compassion Questionnaire (BCQ)	2021	Self-Report	(body kindness) (common humanity) (motivated action)	23	"I like my body in spite of small inadequacies" "I am trying to become more accepting of my body"	5-point Likert-type scale, ranging from "almost never" (1) to "almost always" (5)
Body Compassion Scale (BCS)	2017	Self-Report	(defusion) (common humanity) (acceptance)	23	"When I feel frustrated with my body’s inability to do something, I tend to feel separate and cut off from other people." "When I am at my lowest during times of physical symptoms, illness, or injury, I know I am not alone in feeling this way."	5-point Likert-type scale ranging from 1 (almost never) to 5 (almost always)

Instrument	Published Year	Type of Measure	Subscales/ Forms	Total Number of Items	Sample Item(s)	Response set
Body Image–Acceptance and Action Questionnaire (BIAAQ)	2013	Self-Report	No Subscales	5	"Worrying too much about my weight makes it difficult for me to live a life that I value"<br>"To control my life, I need to control my weight."	7-point Likert-type scale ranging from 1 (never true) to 7 (always true)
Body-Image Contextualized Adaptation of the Self-Compassion Scale (BI-SCS)	2003	Self-Report	(Self-Kindness) (Self-Judgment) (Common Humanity) (Isolation) (Mindfulness) (Over-Identification)	26	"I am disapproving and judgmental about my own flaws and inadequacies"<br>"I obsess and fixate on everything that was wrong"	5-point Likert type scale with anchors "almost never" and "almost always"
Bolton Compassion Strengths Indicators (BCSIs)	2020	Self-Report	(Self-care) (Character) (Empathy) (Connection) (Interpersonal) (Engagement) (Competence) (Communication)	48	"I believe in myself no matter what."<br>"Respecting the patient is just as important as the care they receive."	1: Definitely not like me, 2: Generally not like me, 3: Slightly not like me, 4: Slightly like me, 5: Generally like me, 6: Definitely like me
Breaking Bad News (BBN) Assessment Tool [Unnamed]	2019	Self-Report	No Subscales	21	"Overall, how would you rate this clinician's communication with you?"<br>"The doctor demonstrated that he or she was able to recognize the impact of this moment on me"	5-point Likert-type scale ranging from 1 (strongly disagree) to 5 (agree)
Brief Assessment of Social Skills - Dementia (BASS-D)	2020	Self-Report	(face emotion perception task) (facial identification task) (empathy/theory of mind task) (social disinhibition) (social reasoning task) (memory for familiar faces task)	7	Multiple different types of tasks with pictures	Multiple different types of tasks with pictures
Brief Dimensional Apathy Scale (b-DAS)	2020	Self-Report	(executive) (emotional) (initiation)	9	"I struggle to emphathise with other people"<br>"I became emotional easily when watching something happy or sad on TV"	4-point Likert scale (ranging from 0 to 3)
Brief Patient Satisfaction Scale [Unnamed]	2011	Peer/Corollary Report	No Subscales	10	"I am satisfied that my doctor has been taking care of me."<br>"My doctor explains the reason(s) for any medical test."	7-point Likert scale (1=strongly disagree, 7=strongly agree)
Burnout Measure - Short Version	1988	Self-Report	No Subscales	10	Each item is a continuation of the statement "When you think about your work overall, how often in the last month have you felt…"	seven-point Likert scale ranging from "Never" to "Always"
Burns-Specific Patient-Reported Experience Measure (PREM) [Unnamed]	2019	Self-Report	(care) (compassion and dignity) (information and decision making) (the burns team) (involving others) (facilities) (looking after burn)	23	"Feeling care for"<br>"Treated as a whole person"	5-point Likert-type scale ranging from 1 (strongly disagree) to 5 (strongly agree)
Callous-Unemotional Problems and Impairment (CUPI)	2006	Behavioural/Expert Coder	No Subscales	7		
Calm Compassionate Care Scale (CCCS)	2006	Self-Report	No Subscales	20	"I can be peaceful and focused when my body is quiet and still and it is quiet in my environment."<br>"I can extend kindness, peace, and compassion to patients, colleagues, and myself."	11-point Likert-type scale ranging from 0 (not confident) to 10 (very confident)
Care Receiver View of Caregiver Role Enactment Scale	1985	Self-Report	(affection exhibited by the caregiver in providing care) (attentiveness from the caregiver to things important to the care receiver) (skill of the caregiver in providing care)	12	"How often does your family member make sure you have a chance to do social activities that are important to you?"<br>"How often does your family member do special things for you that you count on and look forward to?"	5-point Likert-type scale ranging from 0 (never) to 4 (always)
Caring Assessment for Care Givers	2011	Self-Report	(maintaining belief) (knowing) (being with) (doing for) (enabling/informing)	25	"I focus on accomplishing the tasks necessary to provide care."<br>"I focus on caring for the whole person (body, mind, spirit) with concentration on facilitating healing."	5-point Likert-type scale ranging from 1 (highly task oriented) to 4 (high degree of rational orientation)
Caring Assessment Tool - Version V (CAT-V)	2014	Self-Report	(nurse–patient communication) (feeling cared for)	27	"Feeling cared for"<br>"Treated as an individual"<br>"Treated as a whole person"	5-point Likert scale ranging from 1 (never) to 5 (always)
Caring Assessment Tool (CAT)	1990	Self-Report	(nurse–patient communication) (feeling cared for)	36	"Feeling cared for"<br>"Treated as an individual"<br>"Treated as a whole person"	5-point Likert scale ranging from 1 (never) to 5 (always)
Caring Behavior Coding Scheme (CBCS)	2020	Self-Report	(Knowing) (Being with) (Doing for) (Enabling)	25	"Centering on the one being cared for/Believing in or holding in esteem."<br>"Protecting dignity/Offering a hope-filled attitude."	
Caring Behaviors Inventory - Short Form (CBI-24)	1994	Self-Report	(assurance) (knowledge and skill) (respectful) (connectedness)	24	"Treating the patient as an individual"<br>"Attentively listening to the patient"<br>"Supporting the patient"	6-point Likert scake ranging from 1 (never) to 6 (always)
Caring Behaviors Inventory (CBI)	1979	Self-Report	No Subscales	42	"Treating the patient as an individual" "Attentively listening to the patient"<br>"Supporting the patient"	6-point Likert scake ranging from 1 (never) to 6 (always)
Caring Culture Survey	2015	Self-Report	(personal caring) (leadership caring) (coworker caring)	24	"I often show empathy and caring to my coworkers"<br>"I feel a lot of empathy toward my patients and their families"	
Caring Dimensions Inventory - 35-Item Version (CDI-35)	1996	Self-Report	(intimacy) (technical) (unnecessary) (supporting) (inappropriate)	35	"Getting to know the patient as a person"<br>"Feeling sorry for a patient"	5-point Likert-type scale ranging from 1 (strongly disagree) to 5 (strongly agree)

Instrument	Published Year	Type of Measure	Subscales/ Forms	Total Number of Items	Sample Item(s)	Response set
Caring Dimensions Inventory (CDI)	1997	Self-Report	No Subscales	41	"Getting to know the patient as a person" "Feeling sorry for a patient"	5-point Likert-type scale ranging from 1 (strongly disagree) to 5 (strongly agree)
Caring Efficacy Scale (CES)	1997	Self-Report	No Subscales	28	"I am able to tune into a particular client/patient and forget my personal concerns" "Even when I really try, I can't get through to difficult clients/patients"	6-point, Likert-type self-report scale (strongly disagree −3 to strongly agree +3)
Caring for Bliss Scale	2017	Self-Report	No Subscales	4	"I search for lasting happiness inside myself, rather than outside of myself" "I listen deeply to my heart"	respondents were instructed to indicate how often each of the statements were true for them on a 5- point rating scale ranging from 0 (never) to 4 (regularly)
Caring Nurse-Patient Interaction - Short Scale (CNPI-Short Scale)	2006	Peer/Corollary Report	(humanistic care) (relational care) (clinical care) (comforting care)	23	"Treat them as complete individuals, show that I was interested in more than their health problem" "Show that I will be there for them if they need me."	1 to 5 points (almost never = 1, sometimes = 2, often = 3, very often = 4, almost always = 5).
Caring Nurse–Patient Interaction Scale (CNPI)	2004	Peer/Corollary Report	(humanism) (hope) (sensitivity) (helping relationship) (expression of emotions) (problem solving) (teaching) (environment) (needs) (spirituality)	70	"Treat them as complete individuals, show that I was interested in more than their health problem" "Show that I will be there for them if they need me."	1 to 5 points (almost never = 1, sometimes = 2, often = 3, very often = 4, almost always = 5).
Caring Scale for Institutionalized Elders	2016	Self-Report	(being supported physically) (being valued)	33	"Provide services that make me feel cared for" "Respond quickly to my discomfort"	5-point Likert-type format for frequency of caring, we used hand signals (five fingers = always, four fingers = more than half of the time, three fingers = half of the time, two fingers = less than half of the time, and one finger = never)
Carkhuff Scale	1969	Self-Report	No Subscales	5	Communicating little or no awareness or understanding of the caller's feelings	seven-point scale for degree of empathy
Carolina Opinions on Care of Older Adults (COCOA)	2007	Self-Report	(early interest in geriatrics) (empathy/compassion) (attitudes towards geriatrics careers) (ageism) (clinical and social services for older adults) (social value of older adults)	24	"Caring for patients in nursing homes would be rewarding" "Older adults are valuable contributors to our society."	Scored on a 1–5 Likert-type (Agreement)
Charge Nurse Support Scale (CNSS)	2003	Peer/Corollary Report	No Subscales	6	"My charge nurse recognizes the standards of care I try to deliver." "My charge nurse knows me well enough to know when I have concerns about patient care and tries to understand my point of view."	5-point Likert-type scale ranging from 1 (not at all true) to 5 (always true)
Child and Adolescent Dispositions Scale (CADS)	2008	Peer/Corollary Report	(dispositional sympathy) (proso ciality) (negative emotionality) (caring) (respect for rules)	57	"Concerned about others when they are hurt" "Feels sorry for kids who get picked on"	1. Not at all, 2. Just a little, 3. Pretty much, 4. Very much.
Child Care Worker Interview	1985	Self-Report	No Subscales	50	"Relationship" "Empathy" "Responsibility"	a 50-item structured interview consisting of 10 theme sets of five questions each.
Child-Related Values Survey (CRVS)	1990	Self-Report	(affective valence) (empathic caring) (self-sacrifice) (societies' duties) (instrumentality) (authoritarian attitude)	43	It hurts me when I see a child's feelings hurt "It makes me feel good to know a child is happy"	
Childhood Narcissism Scale (CNS)	2008	Self-Report	No Subscales	10	"These kids feel bad if they see another kid without a friend to play with" "It makes me sad to see a girl who can't find anyone to play with"	4-point Likert-type response scale with 0 = not at all true, 1 = not really true, 2 = sort of true, and 3 = completely true
Children's Concentration and Empathy Scale (CCES)	2021	Peer/Corollary Report	(concentration) (choosing challenging activities) (being happy) (showing empathetic concern) (appreciating beauty) (realistic ventures)	21	"I try to be understanding and patient towards those aspects of my personality I don't like." "When I fail at something important to me I become consumed by feelings of inadequacy."	5-point scale ("Never/Not at all characteristic of child" to "Always/Very characteristic of child"
Clinical Skills Assessment (CSA)	2002	Self-Report	(interviewing) (counselling) (rapport) (personal manner) (satisfaction)	12	"Ability to be on his or her way to establishing a caring relationship with a patient" "Attentiveness, body language, including appropriate eye contact and respect of personal space, attitude, and demonstration of empathy and support for the patient's concerns"	4-point Likert type scale from 1 (poor) to 4 (excellent)
Clinician and Group Consumer Assessment of Healthcare Providers and Systems Survey (CG-CAHPS)	2018	Self-Report	No Subscales	5	"How often do you feel your provider cares about your emotional or psychological well-being?" "How often do you feel your provider is interested in you as a whole person?"	Responses: 1, Never 2, Sometimes 3, Usually 4, Always

Instrument	Published Year	Type of Measure	Subscales/ Forms	Total Number of Items	Sample Item(s)	Response set
Clinician Associative Stigma Scale (CASS)	2017	Self-Report	(negative stereotypes about professional effectiveness) (discomfort with disclosure) (negative stereotypes about people with mental illness) (stereotypes about professionals' mental health)	18	"I have heard other people say that the work I do is easy/could be done by anyone" "When I have met a new person at a social gathering, I am reluctant to discuss my work with people with serious mental illness"	report the frequency of experiences on the following scale: 1, if the experience had never occurred; rarely, 2, if it had occurred only once or twice; sometimes, 3, if it had occurred repeatedly but irregularly; and often, 4, if it occurred regularly
Cognitive, Affective, and Somatic Empathy Scale (CASES)	2018	Self-Report	(positive emotion- cognitive) (positive emotion- affective) (positive emotion- somatic empathy) (negative emotion- cognitive) (negative emotion- affective) (negative emotion- somatic empathy)	30	"Seeing people sad at a funeral would make me feel sad too" "I feel pleased when someone tells me some good news that they had"	3-point Likert scale from 0 (rarely) to 2 (often)
Collaborative Behaviors Observational Assessment Tool (CBOAT)	2020	Behavioural/Expert Coder	No Subscales	26	"Observed participant covers patient with blanket" "Observed participant shows support to the actor during code verbally."	Yes or NO
Compassion Competence Scale (CCS)	2016	Self-Report	(Communication) (Sensitivity) (Insight)	18	"I can show my compassion toward patients through communication with them." "I try to support patients through care that helps them overcome their problems."	5-point Likert scale (1 = strongly disagree to 5 = strongly agree)
Compassion Fatigue Self-Test (CFST)	1995	Self-Report	(PTSD) (burnout)	120	"I find my life satisfying." "I feel connected to others"	6-point Likert type scale from 0 (Never) to 5 (Very Often)
Compassion Focused Therapy Therapist Competence Rating Scale (CFT-TCRS)	2020	Peer/Corollary Report	(unique competencies) (microskills)	23		
Compassion Motivation and Action Scales (CMAS) for Compassion	2017	Self-Report	(Intention) (Distress Tolerance) (Action)	12	"I want to be kinder and more caring towards people I don't know" "I plan to take action and be more helpful to others"	7-point Likert scale (1 = strongly disagree to 7 = strongly agree)
Compassion Motivation and Action Scales (CMAS) for Self-Compassion	2017	Self-Report	(Intention) (Distress Tolerance) (Action)	18	"I wish to be kind and caring towards myself when faced with difficulties" "I want to be supportive towards myself when I face disappointments in my life"	7-point Likert scale (1 = strongly disagree to 7 = strongly agree)
Compassion Scale	2011	Self-Report	(generosity) (hospitality) (objectivity) (sensitivity) (tolerance)	10	"How much of your future savings would you give away now to help a stranger in need of financial help" "How many times would you deny yourself the pleasure of something that cuases others pain"	response categories (ranging from not true of me to very true of me)
Compassion Scale (CS)	2019	Self-Report	(kindness) (common humanity) (mindfulness) (indifference)	16	"I like to be there for others in times of difficulty" "My heart goes out to people who are unhappy"	5-point Likert scale from 1 (almost never) to 5 (almost always)
Compassionate Care Assessment Tool (CCAT)©	2013	Self-Report	(meaningful connection) (patient expectations) (caring attributes) (capable practitioner)	28	"Being empathetic" "Considering of personal needs"	6-point Likert scale from never to always
Compassionate Engagement and Action Scales (CEAS)	2017	Self-Report	(Engagement) (actions) (Sensitivity to Suffering) (Engagement with suffering)	39		10-point Likert scale (1 = 'never' to 10 = 'always')
Compassionate Love Scale (CLS)	2005	Self-Report	No Subscales	21	"When I see people I do not know feeling sad, I feel a need to reach out to them." "It is easy for me to feel the pain (and joy) experienced by others, even though I do not know them."	7-point Likert-type scale ranging from 1 (not at all true of me) to 7 (very true of me)
Compassionate Love Scale for Humanity - Short Form (CLS-H-SF)	2020	Self-Report	No Subscales	9	"When I hear about someone (a stranger) going through a difficult time, I feel a great deal of compassion for him or her" "It is easy for me to feel the pain (and joy) experienced by others, even though I do not know them"	7-point Likert scale with 1 = "Not at all true of me" and 7 = "Very true of me"
Complementary Measure of Psychotherapy Measure (COMPO)	2021	Self-Report	(Self-acceptance) (Self-knowledge) (Relationship quality) (Consideration of others)	12	"People tell me that I am understanding" "I try to consider how others feel about my criticism before I voice them"	Likert-type scale, with response options of 1 = strongly disagree, 2 = disagree, 3 = slightly disagree, 4 = neither agree nor disagree, 5 = slightly agree, 6 = agree, 7 = strongly agree
Comprehensive Object Relations Profile (CORP)	1984	Self-Report	(object constancy) (object integration) (empathy)	6		
Comrey Personality Scales (CPS)	1970	Self-Report	(shyness) (dependence) (empathy) (neuroticism) (compulsion) (hostility) (socialization)	216	"A sad movie makes me feel like crying" "I am rather insensitive to the difficulties that other people are having."	7-point Likert-type scale ranging from 1 (never or definitely not) to 7 (always or definitely)
Connection Index (CI12)	2022	Self-Report	(psychological safety) (edicational alliance) (empathy) (feedback)	12	"Overall, I felt comfortable asking questions of, asking for help from, and admitting mistakes to this person" "Overall, this person could understand my experience and perspective"	7-point Likert item scales. Scores ranged from 1 to 7, with higher scores indicating more favorable connections.

Instrument	Published Year	Type of Measure	Subscales/ Forms	Total Number of Items	Sample Item(s)	Response set
Connor-Davidson Resilience Scale 10 (CD-RISC-10)	2003	Self-Report	No Subscales	7	"I am able to adapt to change."<br>"I can deal with whatever comes."	5-point Likert, 0 = not true at all, 4 = true nearly all the time
Consultation and Relational Empathy (CARE) Measure	2002	Self-Report	No Subscales	19	"How was the doctor at fully understanding your concerns?"<br>"How was the doctor at showing care and compassion?"	5-point Likert-type scale ranging from 1 (Poor) to 5 (Excellent)
Consumer Orientation Questionnaire (SCOI)	2000	Self-Report	No Subscales	35		
Contingency Management Competence Scale (CMCS)	2009	Behavioural/Expert Coder	(general) (draw)	12	"To what extent did the therapist state how many draws were earned at this session?"<br>"Empathy (conveys warmth and sensitivity, demonstrates genuine concern and a non-judgmental stance, understands/expresses clients' feelings and concerns)."	7-point scale, with higher numbers reflecting better usage. Competence ratings anchored the 1 with the phrase “Very poor,” and the 7 was anchored with the word “Excellent.”
Counselor Behavior Analysis (CBA)	1986	Self-Report	(counselor adaptability) (counselor empathy) (counselor talkativeness) (counselor overall effectiveness)	24		
Countertransference Management Scale (CMS)	2017	Peer/Corollary Report	(understanding self and client) (self-integration and regulation)	22	"Grasps theoretically clients’ dynamics in terms of what goes on in the therapeutic relationship"<br>"Effectively connects strands of clients’ material in developing conceptualizations of clients"	5-point scale where 1 strongly disagree, 3 neither agree nor disagree, and 5 strongly agree
Curtin Vicarious Response Scales (CVRS)	2012	Self-Report	(perspective taking) (empathy) (emotional lability)	28		
Decentering Scale for Sport (DSS)	1996	Self-Report	No Subscales	28	"During training and competition……<br>I can pull myself out of annoying thoughts or images without being controlled by them."<br>I can distinguish thoughts which are objective reflections from those which are my personal thinking."	5-point Likert-type scale ranging from 1 (never true) to 5 (always true)
Defending Behaviors Scale (DBS)	2020	Self-Report	(reporting to authority) (comforting) (aggressive) (solution-focused)	20	"When I'm upset at someone, I usually try to put myself in his shoes for a while"<br>"I often have tender concerned feeling for people less fortunate than me"	5-point Likert type scale from 0 (doesn't describe me at all) to 4 (describes me very well)
Dental Beliefs Survey - Revised (DBS-R)	1995	Self-Report	(ethics) (communication) (control) (trust)	28	"Dentists do not like patient requests."<br>"Dentists seem to be in a hurry, so I feel rushed."	5-point Likert-type scale ranging from 1 (never) to 5 (nearly always)
Dental Home Scale (DHS)	2019	Peer/Corollary Report	No Subscales	10	"During the last 2 years, how often did dentists or their staff show respect for what you had to say?"<br>"During the last 2 years how often have you felt that dentists or their staff treated you unfairly because of your race?"	Always/usually/sometimes/Never/DK
Dentist-Patient Relationship Skills Assessment [Unnamed]	2012	Self-Report	(verbal and nonverbal communication) (empathy and assertiveness) (behavioral characteristics and personality traits) (pharmacological and nonpharmacological control techniques)	44	"Listens actively, interspersing expressions and questions, showing attention to the patient's history"<br>"Expresses kindness, reflecting a positive attitude of willingness to help"	
Diabetes-Specific Self-Compassion Scale (SCS-Dp)	2003	Self-Report	No Subscales	19	"When I fail at something important to me, I become consumed by feelings of inadequacy."<br>"I try to see my failing as part of the human condition."	5-point Likert-type scale ranging from 1 (almost never) to 5 (almost always)
Dietrich Object Relations and Object Representations Scale (DOROS)	1985	Peer/Corollary Report	(object investment) (sadistic vs neutralized aggression) (object warmth) (quality/level of object relatedness) (empathy) (object love) (superego harshness vs superego benevolence) (helplessness vs internal sense of control) (psychological mindedness) (degree of depression)	4		
Driving While Impaired Therapeutic Educator Inventory (DTEI)	2001	Self-Report	(judicial) (disease) (structure) (relationship) (interaction) (empathic-supportive)	69	“Genetics play the primary role in alcoholism”	6-point Likert scale from 0 (strongly disagree) to 5 (strongly agree)
DSM-5 Level of Personality Functioning Questionnaire (DLOPFQ)	2017	Self-Report	(self-direction) (identity) (empathy) (intimacy)	66	“It’s difficult to believe others when they tell me that I do not understand them”<br>“It is a waste of time to think about my or others’personal reactions"	6-point Likert type scale from 1 (strongly disagree) to 6 (strongly agree)
Dymond's Test of Insight and Empathy	1949	Self-Report	No Subscales	24	"Friendliness"<br>"Sympathy"	5-point scale

Instrument	Published Year	Type of Measure	Subscales/ Forms	Total Number of Items	Sample Item(s)	Response set
ECRC Altruism Scale	2021	Self-Report	No Subscales	5	"I really care about the needs of other people"<br>"I enjoy doing things for others"	5-point Likert scale (i.e., strongly disagree = 1, disagree = 2, neither agree nor disagree = 3, agree = 4, strongly agree = 5)
Edinburgh Social Cognition Test (ESCoT)	2018	Behavioural/Expert Coder	(Cognitive ToM) (Affective ToM) (Interpersonal understanding of social norms) (Intrapersonal understanding of social norms)	5	"What is X thinking?"<br>"How does X feel at the end of the animation?"	N/A
Effects of Meditation (EOM) Scale	2009	Self-Report	(cognitive effects) (mystical experiences) (relaxation) (physical discomfort and emotional effects) (effects of meditation in everyday life)	18	"I experience fluctuating emotions"<br>"I observe without judgement any negative thoughts or emotions that arise"	5-point Likert-type scale ranging from 1 (almost never) to 5 (almost always)
Eight-Item Toronto Alexithymia Scale (TAS-8)	2020	Self-Report	(Difficulty identifying feelgs) (Difficulty describing feelings) (Externally oriented thinking)	8	"I am often confused about what emotion I am feeling"<br>"It is difficult for me to find the right words for my feelings"	Five-point Likert scale items from Strongly Disagree to Strongly Agree
Emotion Specific Empathy (ESE) Questionnaire	2014	Self-Report	(anger affective empathy) (anger cognitive empathy) (disgust affective empathy) (disgust cognitive empathy) (fear affective empathy) (fear cognitive empathy) (happy affective empathy) (happy cognitive empathy) (sad affective empathy) (sad cognitive empathy) (surprise affective empathy) (surprise cognitive empathy)	60	"When I see someone being taken advantage of, I feel kind of protective toward them"<br>"I cannot continue to feel OK if people around me are depressed"	7-point response scale ranging from -3 (disagree strongly to 3 (agree strongly
Emotional Accuracy Research Scale (EARS)	1996	Self-Report	(consensus-agreement) (target-agreement score)	24	The suffering of others deeply disturbs me	multiple choice and forced choice items
Emotional Intelligence (EI) Instrument	2000	Self-Report	(maturity) (compassion) (morality) (sociability) (calm disposition)	34	"I sympathize with others when they have problem"<br>"I keep myself focused on my goals"	5-point Likert-type scale ranging from 1 (never like me) to 5 (always like me)
Emotional Intelligence Inventory (EII)	1998	Self-Report	(handling relationships) (utilization of feelings) (empathy) (self-control)	172	"I sympathize with others when they have problem"<br>"I keep myself focused on my goals"	5-point Likert-type scale ranging from 1 (never like me) to 5 (always like me)
Emotional Intelligence Scale (EIS)	1998	Self-Report	(optimism/mood regulation) (appraisal of emotions) (social skills) (utilization of emotions)	33	"I know when to speak about my personal problems to others"<br>"I expect I will do well on most things I try"	5-point Likert-type scale ranging from 1 (strongly disagree it is like me) to 5 (strongly agree this is like me)
Emotional Intimacy Scale (EIS)	2005	Peer/Corollary Report	No Subscales	5	"This person completely accepts me as I am."<br>"I can openly share my deepest thoughts and feelings with this person."	5-point Likert scale ranging from 1 (rarely) to 5 (a great deal of time)
Emotional Logic Development Profile (ELDP)	1995	Self-Report	No Subscales	36	"I feel confident in myself."<br>"I can talk about my feelings."	0 = clearly individual; 1 = borderline; 2 = clearly relational.
Emotional Response Questionnaire (ERQ)	2018	Self-Report	No Subscales	6	"I often have tender or concerned feelings for people less fortunate than me"<br>"I try to look at everybody's side of a disagreement before I make a decision"	Likert rating scale (ranging from 'not at all' to "very much')
Emotional Skills and Competence Questionnaire (ESCQ)	1997	Self-Report	(perceive and understand emotions) (express and label emotions) (manage and regulate emotions)	45	"When I see how someone feels, I usually know what has happened to him"<br>"I notice when somebody feels down"	6-point Likert-type scale ranging from 1 (never) to 6 (always)
Empathetic Care Scale (ECS)	2013	Self-Report	(extra-role behavior) (relational richness) (emotional engagement)	10	"I help my clients feel better when they are down"<br>"My clients' emotional state is just as important as their physical state"	strongly disagree (1) to strongly agree (7) scales
Empathetic Understanding Assessment (EUA)	2007	Behavioural/Expert Coder	(maternal representational ability) (mothers views of their children's emotional experience) (mothers' views of their own emotion)			
Empathic Communication Coding System (ECCS)	2002	Self-Report	No Subscales	N/a	"Statement of shared feeling and experience"<br>"Implicit recognition of patient perspective"	ECCS used to code physician responses to empathic opportunities
Empathic Counselor Response Scale (ECRS)	2020	Behavioural/Expert Coder	No Subscales	24		Likert-type scale of very unempathic (1), somewhat unempathic (2), somewhat empathic (3), and very empathic (4)
Empathic Experience Scale (EES)	2018	Self-Report	(vicarious experience) (intuitive understanding)	30	"I easily get carried away by the lyrics of a love song"<br>"I don't get overly involved with friends' problems"	5-point Likert-type scale ranging from 1 (Not at all true) to 5 (Completely true)
Empathic Expressions Scale (EES)	2021	Self-Report	(Verbal Affirmation) (Experience Sharing) (Empathic Voice) (Emotional Reactvity) (Empathic Touch)	28	"When people are feeling negative emotion, I encourage them by saying that they will get through this"<br>"I tell people I know what it is like to feel the way they do"	7-point Likert-scale ranging from 1 = never to 7 = very often

Instrument	Published Year	Type of Measure	Subscales/ Forms	Total Number of Items	Sample Item(s)	Response set
Empathic Tendency Scale (ETS)	1988	Self-Report	(empathic skill) (empathic environment) (anti-empathic attitude)	29	"Interested in the problems of those around me as much as mine"<br>"I am gentle to understand that people get cried while watching movies"	5-point Likert scale ranging from “totally agree” to “totally disagree”.
Empathic Understanding Rating Scale	1976	Behavioural/Expert Coder	No Subscales	N/a		
Empathy Accuracy (EA) Task	2009	Self-Report	No Subscales	27	picture items	rate the perceived emotional intensity of each target using a 9-point scale from 1 (no emotion) to 9 (very strong emotion)
Empathy and Clarity Rating Scale (ECRS)	2015	Behavioural/Expert Coder	(fostering relationship/supporting emotion) (gathering information) (providing information) (helping the patient to make decisions)	75	"Sets the stage for the encounter and ongoing care and made patient feel at ease"<br>"Listens actively and conveys full understanding of patient’s concerns"	5 (desired), 4 (Needs minimal adjustment), 3 (Skills developing), 2 (Needs significant adjustment), 1 (Unsatisfactory)
Empathy Assessment Index (EAI)	2010	Self-Report	(affective response) (self-other awareness) (perspective-taking) (emotion regulation) (empathic attitude)	54	"When a friend is sad, I become sad"<br>"I feel what another person is feeling even when I don't know the person"	5-point Likert-type scale ranging from 1 (Never) to 5 (Always)
Empathy Assessment Scale (EAS)	2022	Self-Report	(Social Interaction) (Cognitive Behavior) (Emotional Identification)	13	“Being together with a sad person, I feel sad too”<br>“I sincerely congratulate my successful opponent”	5-point Likert-type grading was used as never, rarely, sometimes, often, and always
Empathy Clinical Evaluation Exercise (ECEX)	2019	Behavioural/Expert Coder	No Subscales	4		
Empathy Components Questionnaire (ECQ)	2017	Self-Report	(cognitive ability) (cognitive drive) (affective ability) (affective drive) (affective reactivity)	39	"It affects me very much when one of my friend is upset"<br>"I get very upset when I see someone cry"	4-point response scale ranging from 1 (strongly disagree) to 4 (strongly agree)
Empathy Construct Rating Scale (ECRS)	1981	Self-Report	(nonverbal behaviour) (personality traits) (sensitivity to another's world) (responding) (respect for self and others)	100	Seems to understand another person's state of being.” 6-point Likert scales	
Empathy for Action Pain Picture Database (EPSS-Action_Picture)	2023	Self-Report	(Pain intensity) (Affective valence) (Arousal) (Dominance)	478		9 point rating scales per dimension: pain intensity (1 = no sensation, 4 = pain threshold, 9= most intense pain imaginable), affective valence (1 = very unhappy, 9 = very happy), arousal (1=extremely peaceful, 9 = extremely excited), dominance (1= extremely out of control, 9 = extremely in control)
Empathy for Action Pain Video Database (EPSS-Action_Video)	2023	Self-Report	(Pain intensity) (Affective valence) (Arousal) (Dominance)	478		9 point rating scales per dimension: pain intensity (1 = no sensation, 4 = pain threshold, 9= most intense pain imaginable), affective valence (1 = very unhappy, 9 = very happy), arousal (1=extremely peaceful, 9 = extremely excited), dominance (1= extremely out of control, 9 = extremely in control)
Empathy for Face Pain Picture Database (EPSS-Face)	2023	Self-Report	(Pain intensity) (Affective valence) (Arousal) (Dominance)	160		9 point rating scales per dimension: pain intensity (1 = no sensation, 4 = pain threshold, 9= most intense pain imaginable), affective valence (1 = very unhappy, 9 = very happy), arousal (1=extremely peaceful, 9 = extremely excited), dominance (1= extremely out of control, 9 = extremely in control)
Empathy for Limb Pain Picture Database (EPSS-Limb)	2023	Self-Report	(Pain intensity) (Affective valence) (Arousal) (Dominance)	136		9 point rating scales per dimension: pain intensity (1 = no sensation, 4 = pain threshold, 9= most intense pain imaginable), affective valence (1 = very unhappy, 9 = very happy), arousal (1=extremely peaceful, 9 = extremely excited), dominance (1= extremely out of control, 9 = extremely in control)
Empathy for Pain Scale (EPS)	2014	Self-Report	(affective distress) (empathic concern) (vicarious pain)	48	"Sense of compassion"<br>"Sense of what it feels like"	5-point response scale ranging from 1 (strongly disagree) to 5 (strongly agree)

Instrument	Published Year	Type of Measure	Subscales/ Forms	Total Number of Items	Sample Item(s)	Response set
Empathy for Voice Pain Database (EPSS-Voice)	2023	Self-Report	(Pain intensity) (Affective valence) (Arousal) (Dominance)	60		9 point rating scales per dimension: pain intensity (1 = no sensation, 4 = pain threshold, 9= most intense pain imaginable), affective valence (1 = very unhappy, 9 = very happy), arousal (1=extremely peaceful, 9 = extremely excited), dominance (1= extremely out of control, 9 = extremely in control)
Empathy Gradient Questionnaire (EGQ)	2017	Self-Report	(family empathy) (friend empathy) (peer/colleague empathy) (distant other empathy) (species empathy)	23	"I worry about people living in poverty overseas" "I become upset when I see someone hurt, even when I do not know the person very well"	5-point response scale ranging from 1 (very much disagree) to 5 (very much agree)
Empathy Index (EI)	2016	Self-Report	(emotional contagion) (behavioral contagion)	14	"If I see someone fidgeting, I'll start feeling anxious too" "If I see a video of a baby smiling, I find myself smiling"	5-point Likert-type scale
Empathy Measure for Parents Analog Task - Emotion Audio (EMPAT-EA)	2021	Self-Report	(Happy) (Mad) (Sad) (Scared)	16	Happy, Mad, Cheerful, Sad	5-point Likert scale to rate how hearing the child in each clip made them feel
Empathy Measure for Parents Analog Task - Emotion Scripts (EMPAT-ES)	2021	Self-Report	(Happy) (Mad) (Sad) (Scared)	16	Happy, Mad, Cheerful, Sad	5-point Likert scale to rate how hearing the child in each clip made them feel
Empathy Questionnaire for Children and Adolescents (EmQue-CA)	2010	Self-Report	(affective empathy) (cognitive empathy) (intention to comfort)	14	"When a friend is upset, I feel upset too" "If someone in my family is sad, I feel really bad"	5-point Likert type scale from 0 (completely untrue) to 4 (completely true)
Empathy Quotient - Child (EQ-C)	2009	Self-Report	No Subscales	55	"I find it hard to understand how to behave in a social situation" "In a conversation, I tend to focus on my own thoughts rather than on what my listener might be thinking."	5-point Likert-type scale ranging from 0 (strongly disagree) to 4 (strongly agree)
Empathy Quotient - Short (EQ-Short)	2003	Self-Report	No Subscales	22	"I am good at predicting how someone will feel" "I am quick to spot when someone in a group is feeling awkward or uncomfortable"	Likert type scale with half worded "agree" and half worded "disagree" each to variating degree
Empathy Quotient (EQ)	2004	Self-Report	(cognitive empathy) (emotional reactivity) (social skills)	60	"I find it hard to understand how to behave in a social situation" "In a conversation, I tend to focus on my own thoughts rather than on what my listener might be thinking."	5-point Likert-type scale ranging from 0 (strongly disagree) to 4 (strongly agree)
Empathy Scale for Social Workers (ESSW)	2012	Self-Report	(compassionate contextual assessment) (accepting and attentive collaborative inquiry) (intrinsic helping and emotional support)	41	"I am kind to my clients" "I am attentive to my clients' nonverbal cues"	5-point Likert-type scale ranging from 1 (never) to 5 (always)
Empathy Selection Task	2010	Self-Report	No Subscales	N/a		
Empathy Test	1979	Self-Report	No Subscales	17		
Empathy Towards People with Intellectual Disabilities Questionnaire (EMP-ID)	2015	Self-Report	(proximity) (challenge) (active attunement)	20	"I get angry when I think of how people with intellectual disabilities have been treated" "I always try to tune into the feelings of people with intellectual disabilities around me"	5-point Likert-type scale from 1 (not at all) to 5 (very much)
Empathy-Enhancing Virtual Evolving Environment (EEVEE)	2015	Self-Report	No Subscales	16		
English Youth Self-Report Version of the ICU	2004	Self-Report	(Callous-Unemotional) (Empathic-Prosocial)	18	"I do not care who I hurt to get what I want." "I do not care about being on time."	4-point response scale ranging from 1- Not at all true to 4 – Definitely true
Equanimity Barriers Scale (EBS)	2018	Self-Report	(innate) (social) (interactive) (reflective)	15	"Organisations or institutions influence the way I perceive others?" "I feel that the media influences my feelings towards others?"	7-point response scale ranging from 1 (strongly disagree) to 7 (strongly agree)
Essential Properties of Yoga Questionnaire (EPYQ)	2018	Behavioural/Expert Coder	(acceptance/compassion) (breathwork) (physicality) (active postures (asanas)) (restorative postures (asanas)) (body locks (bandhas)) (body awareness) (mental & emotional awareness/release) (health benefits) (individual attention) (social aspects) (spirituality) (meditation & mindfulness) (yoga philosophy)	61	"How much did the instructor mention or include placing one's focus on the breath?" "How much did the instructor mention or include vigorous activity or physical exertion?"	5-point Likert scale from 1 (not at all) to 5 (a very large amount)
Ethical Wills Assessment	2009	Self-Report	No Subscales	22		
Ethnic Minority Patient-3 (EMP-3)	2010	Self-Report	(physicians task perception and ideas on cultural differences in health and healthcare) (physicians' attitudes towards physician-patient communication with minority patients) (physicians' perception of minority patients' needs in communication)	18	"Physicians should be empathic toward every patient, even if they have completely different opinions" "Physicians have a moral duty toward taking care of refugees"	

Instrument	Published Year	Type of Measure	Subscales/ Forms	Total Number of Items	Sample Item(s)	Response set
Evaluating Goelman's Five Dimensions of Emotional Intelligence [Unnamed]	2013	Self-Report	(self-awareness) (mood management) (self-motivation) (empathy) (managing relationships)	25	"I am adept at recognizing anxiety in others" "I am adept at recognizing nonverbal cues associated with feelings in others"	5-point Likert-type scale ranging from 1 (never) to 5 (always)
Everyday Multicultural Competencies/Revised Scale of Ethnocultural Empathy(EMC/RSEE)	2014	Self-Report	(cultural openness and desire to learn) (resentment and cultural dominance) (anxiety and lack of multicultural self-efficacy) (empathic perspective-taking) (awareness of contemporary racism and privilege) (empathic feeling and acting as an ally)	84	"I get disturbed when other people experience misfortunes due to their racial or ethnic background" "I share the anger of people who are victims of hate crimes"	6-point Likert-type scale from 1 (strongly disagree) to 6 (strongly agree)
Evidence-Based Practice Profile (EBP2) Questionnaire	2010	Self-Report	(relevance) (terminology) (confidence) (practice) (sympathy)	58	"How often have you formulated a clearly answerable question as the beginning of the process towards filling this gap?" "How often have you tracked down the relevant evidence once you have formulated the question?"	7-point Likert scale
Executive Function Index (EFI)	2004	Self-Report	(empathy) (strategic planning) (organization) (impulse control) (motivational drive)	27	"Takes others' feelings into account" "Concern for others"	5-point Likert-type scale (1 = Not at all, 5 = Very much)
Experience Sampling Method (ESM)	2017	Self-Report	(emotion-oriented) (intention-oriented)		"I get very upset when I see someone cry" "It affects me very much when one of my friend seems upset"	4-point Likert scale
Eysenck Personality Inventory (EPI)	1995	Self-Report	(extraversion (neuroticism) (shyness and nervousness) (conscientiousness) (agreeableness) (openness or culture)	57	"Do you ever feel 'just miserable' for no good reason" "Are you often troubled about feelings of guilt"	yes/no
Eysenck Personality Questionnaire - Revised (EPQ-R)	1985	Self-Report	(psychoticism) (extraversion) (neuroticism) (lie)	100	"Do you ever feel 'just miserable' for no good reason" "Are you often troubled about feelings of guilt"	yes/no
Facets of Emotional Experiences in Everyday Life Scale (FEEELS)	2022	Self-Report	(Anger) (Fear) (Joy) (Love) (Sadness) (Surprise)	97	In the past hour, I felt.... [cranky, thankful, amazed, compassionate]	5-point scale: Not at all, somewhat, moderately, quit a bit, extremely
Facilitative Interpersonal Skills for Child Therapy (FIS-C)	2018	Self-Report	(verbal fluency) (hope and positive expectations) (persuasiveness) (emotional expression) (warmth, acceptance and understanding) (empathy) (alliance bond capacity) (alliance rupture repair responsiveness)	7		5-point Likert-type scale, ranging from '1' (not characteristic) to '5' (extremely characteristic)
Faculty Evaluation Instrument [Unnamed]	2000	Peer/Corollary Report	(competency and professionalism) (commitment to knowledge and instruction) (inclusion and interaction) (patient focus) (openness to ideas)	18		
Family Nurse Caring Belief Scale (FNCBS)	2007	Self-Report	No Subscales	25	"I tell the family that I am their supporter" "I am a worker who understand how the family truly feels"	5 point Likert type scale (1-strongly disagree to 5-strongly agree)
Family Response Questionnaire (FRQ)	2000	Self-Report	(sympathetic–empathic) (active engagement) (rejecting–hostile) (concern with self)	25	"Thought about how X must be feeling?" "Worried about X's illness?"	
Fat Attitudes Assessment Toolkit (FAAT)	2022	Self-Report	(Empathy) (Activism Orientation) (Size Acceptance) (Attractiveness) (Critical Health) (General Complexity) (Socioeconomic Complexity) (Responsibility) (Body Acceptance)	49	"Negative beliefs about body weight lead to negative assumptions about fat people" "Fat people face discrimination in many areas of life"	1 (strongly disagree), 2 (disagree), 3 (mildly disagree), 4 (neither agree nor disagree), 5 (mildly agree), 6 (agree), 7 (strongly agree)
Fears of Compassion for Self Scale	2011	Self-Report	(expressing compassion for others) (responding to compassion for others) (expressing kindness and compassion towards yourself)	38	"Getting on in life is about being tough rather than compassionate" "I fear that if I am more self-compassionate I will become a weak person"	five-point Likert scale (0 = Don't agree at all, 4 = Completely agree)
Fears of Compassion in Sport Scale (FCSS)	2022	Self-Report	(Fear of self-compassion) (Fear of compassion from others)	10	"I fear that if I start to develop compassion for myself, I will become dependent on it" "I fear that if I become too compassionate to myself, I will lose my self-criticism and my flaws will show"	5-point Likert scale ranging from 0 (do not agree at all) to 4 (completely agree)
Fears of Compassion Scales	2011	Self-Report	(expressing compassion for others) (responding to compassion for others) (expressing kindness and compassion towards yourself)	45	"I fear that being too compassionate makes people an easy target" "People need to help themselves rathan than waiting for others to help them"	five-point Likert scale (0 = Don't agree at all, 4 = Completely agree)
Figley's Compassion Fatigue Scale - Revised	1995	Self-Report	(secondary trauma) (job burnout)	13	"Losing sleep over client's traumatic experience" "Intrusive thoughts after working with client"	10-point, visual analog–type Likert scale (rarely/never = 1to very often = 10)

Instrument	Published Year	Type of Measure	Subscales/ Forms	Total Number of Items	Sample Item(s)	Response set
Five Facet Mindfulness Questionnaire (FFMQ)	2006	Self-Report	(Observe) (Describe) (Nonjudge) (Nonreact) (Actaware)	39	"I criticize myself for having irrational or inappropiate emotions." "When I have distressing thoughts or images, I feel calm soon after."	5-point Likert-type scale ranging from 1 (never or very rarely true) to 5 (very often or always true)
Five-Factor Narcissism Inventory - Short Form (FFNI-SF)	2012	Self-Report	(acclaim seeking) (arrogance) (authoritativeness) (distrust) (entitlement) (exhibitionism) (exploitativeness) (grandiose fantasies) (indifference) (lack of empathy) (manipulativeness) (need for admiration) (reactive anger) (shame) (thrill seeking)	60	"I make a point of trying not to hurt others in pursuit of my goals" "I feel bad if my words or actions cause someone else to feel emotional pain"	5-point Likert-type scale ranging from 1 (disagree strongly) to 5 (agree strongly)
Forms of Responding to Self-Critical Thoughts Scale (FoReST)	2020	Self-Report	(unworkable action) (mindful acceptance)	46	"When I have a critical thought about myself, it gets me so down that I don't act in the way I should" "When I have a critical thought about myself, I don't treat others the way I would like"	Likert type scale ranging from 0 to 3
Four Habits Coding Scheme (4HCS)	2006	Behavioural/Expert Coder	(invest in beginning) (elicit patient's perspective) (demonstrate empathy) (invest in end)	23	"Demonstrating familiarity with the patient and greeting the patient warmly" "Helping patients to identify their emotions; using appropriate nonverbal behavior"	5-point scale
Four-Dimensional Connectedness Scale (4DCS)	2011	Self-Report	(other workers) (recipients) (task requirement) (values)	15	"I feel close to the people I am helping" "I enjoy doing volunteer work"	7-point Likert scale ranging from 1 = strongly disagree to 7 = strongly agree
Four-Item Mentalising Index	2021	Self-Report	No Subscales	4	"I find it easy to put myself in somebody else's shoes." "I sometimes find it difficult to see things from other people's point of view."	4-point Likert scale
General Anesthesia Satisfaction Questionnaire	2012	Self-Report	No Subscales	23		
Genuine Happiness Scale (GHS)	2022	Self-Report	No Subscales	4	"I experience an inner joy, no matter what the external circumstances are." "Deep within me I retain an inner peace, regardless of the external circumstances"	a 5-point rating scale ranging from 0 (never) to 4 (regularly)
Global Interprofessional Therapeutic Communication Scale© (GITCS©)	2019	Behavioural/Expert Coder	(empathy) (power sharing) (trust and rapport building)	35	"Offers patient opportunities to organize and express their thoughts." "Encouraged feedback and input from patient/family."	Never, Rarely, Sometimes, Usually, Always, Not Applicable
Global Rating Form for ACGME	1999	Self-Report	(interpersonal communication skills and professionalism) (medical knowledge) (patient care and systems-based care)	23	"Gather essential and accurate information about their patients." "Effectiveness as a team member"	5-point Likert-type scale ranging from 1 (severe deficits) to 5 (outstanding competence)
Global Rating of Empathy Scale (GRE)	2011	Peer/Corollary Report	(perspective taking) (compassionate care) (standing in the patient'sshoes)	20	"It is difficult for me to view things from my patients' perspective" "Patients feel better when their health care providers understand their feelings."	7-point Likert-type scale ranging from 1 (strongly disagree) to 7 (strongly agree)
Grid Measure of Empathy [Unnamed]	1972	Self-Report	No Subscales	N/a		
Griffith Empathy Measure (GEM)	2007	Peer/Corollary Report	(cognitive factor) (affective factor)	23	"'My child gets upset with he/she sees an animal being hurt'' "It makes my child sad to see another child who can't find anyone to play with''	nine-point Likert scale from strongly disagree (4) to strongly agree (+4)
Groningen Reflection Ability Scale (GRAS)	2007	Self-Report	(self-reflection) (empathetic reflection) (reflective commuication)	23	"I can empathize with someone else's situation" "I am able to understand people with a different culture/religious background"	5-point Likert-type scale ranging from 1 (disagree strongly) to 5 (agree strongly)
Group Questionnaire (GQ)	2009	Self-Report	(positive bonding relationship) (positive working relationship) (negative relationship)	30		
Group-Analysis-Empathy 1 (GA-Em1)	2007	Self-Report	(emotional disclosure and sensibility) (containing and metabolizing) (immersion) (resonance and responsiveness) (insight)	40		
Group-Analysis-Empathy 2 (GA-Em2)	2007	Self-Report	(emotional disclosure and sensibility) (containing and metabolizing) (immersion) (resonance and responsiveness) (insight)	40		
Hate Crime Beliefs Scale - UK (HCBS-UK)	2021	Self-Report	(denial) (compassion) (sentencing)	20	"Offenders who target Muslims based on their race deserve a harsher sentence." "A hate crime offender should receive a lengthier prison sentence."	NA
Hate Crime Beliefs Scale (HCBS)	2018	Self-Report	(denial) (compassion) (sentencing)	46	"I am someone who is helpful and unselfish with others" "I am someone who worries a lot"	5-point Likert-type scale ranging from 1 (disagree strongly) to 5 (agree strongly)

Instrument	Published Year	Type of Measure	Subscales/ Forms	Total Number of Items	Sample Item(s)	Response set
Health Care Providers Attitudes Toward FGM/C and Those who Practice	2021	Self-Report	(Attitudes) (Empathetic Attitudes)	12	"FGM/C is a violation of human rights." "Communities that practice FGM/C are honoring an important cultural tradition."	four-point Likert scale from "4 = Strongly Agree" to "1 = Strongly Disagree"
Health Care Providers Confidence in FGM/C Care	2021	Self-Report	(Confidence in Clinical FGM/C Care) (Confidence in Critical Communication Skills for FGM/C Care)	8	"Respond to the health concerns of women with FGM/C by engaging in non-judgmental listening." "Create a positive therapeutic relationship with a patient who refuses a recommended procedure."	four-point Likert scale from "4 = Strongly Agree" to "1 = Strongly Disagree"
Health Coaching Index (HCI)	2021	Behavioural/Expert Coder	(Long-term vision, desigining goals & actions, and self-monitoring) (Use a self-discovery process) (Match information to client interest and needs) (Have a client-centered/mindful approach)	18	"Collaborative partnership" "Express empathy"	3-point scale: 0 (not yet competent), 1 (competent), 2 (proficient)
Health Communication Assessment Tool© (HCAT©)	2013	Self-Report	(rapport) (power sharing) (education/empowering) (empathy) (avoiding miscommunication)	24	"Used positive communication including smile to encourage interaction" "Leaned towards the speaker to show listening intently"	three-point rating scale of yes, no, and unsure
Helpful Responses Questionnaire (HRQ)	1991	Behavioural/Expert Coder	No Subscales	6	"Last night Joe really got high and he came home late and we had a big fight. He yelled at me and I yelled back and then he hit me hard! He broke a window and the TV set, too! It was like he was crazy. I just don't know what to do!"	Free response
Helpful Responses to Refugees Questionnaire (HRRQ)	2015	Behavioural/Expert Coder	No Subscales	5	"Last night Joe really got high and he came home late and we had a big fight. He yelled at me and I yelled back and then he hit me hard! He broke a window and the TV set, too! It was like he was crazy. I just don't know what to do!"	Free response
Hogan Empathy Scale (HES)	1969	Self-Report	No Subscales	64	"As a rule I have little difficulty in 'putting myself into other people's shoes'" "I have seen some things so sad that I almost felt like crying"	
Hospice Volunteer as Patient Advocate (HVPA) Scale	1998	Self-Report	(patient rights) (volunteer duty)	25	"My failures are due to the people surrounding me" "My life depends on myself"	5-point Likert-type scale ranging from 1 (disagree strongly) to 5 (agree strongly)
Hospital Nurse Interpersonal Empathy Questionnaire (HNEIQ)	2018	Self-Report	(empathetic and ethical attention) (perspective adoption) (emotional affectability) (altruism) (emotion identification and responsivity) (reflection forecasting)	45	"Colleagues' grief and sorrow make me feel bad" "I feel unhappy at disrespect towards a colleague"	5-point Likert-type scale ranging from 1 (rarely) to 5 (always)
Huddersfield Service User Pedagogy Scale	2018	Self-Report	(service user involvement in teaching) (learning in response to service user involvement in teaching)	19	"Service users' feelings are much more important than keeping to appointment times" "The service user's story is the most important aspect in making professional decisions"	5-point Likert-type scale ranging from 1 (disagree strongly) to 5 (agree strongly)
Human Empathetic Listening Test (HELT)	1976	Self-Report	(understanding) (interest) (response-ability)	60		
Human Factors Skills for Healthcare Instrument - Auxiliary Version (HuFSHI-A)	2019	Self-Report	No Subscales	12	"When my collegues are upset, I can help them calm down" "I can show others that I care, even when I am under stress"	scale from 1 to 10
Humanised Care Assessment Tool (HCAT)	2018	Self-Report	No Subscales	109	"I feel I should give something back to others" "Because I had cancer I am more understanding of what other people feel"	5-point Likert-type scale ranging from 1 (strongly disagree) to 5 (strongly agree)
Humanistic Relationship Importance Scale (HRIS)	2010	Self-Report	(relational availability) (promoting quality of daily life) (recognizing and supporting choice) (forming connections) (supporting human uniqueness)	93	"The nurses take time to listen to your concerns" "You and the nurses feel close to each other"	5-point scale from "not important" to "very important"
Illness Invalidation Inventory (3*I)	2010	Self-Report	(spouse) (family) (medical professionals) (work environment and social services)	8	"Spouse/family/medical professionals/work environment/social services takes me seriously" "...Thinks I should be tougher"	five-point Likert-type scale (1 = never to 5 = very often)
Impact of Cancer Scale - Version 2 (IOCv2)	2006	Self-Report	(altruism and empathy) (health awareness) (meaning of cancer) (positive self-evaluation) (appearance concerns) (body change concerns) (life interferences) (worry)	27	"Having had cancer has made me more willing to help others" "Because I had cancer I am more understanding of what other people feel"	5-point Likert-type scale ranging from 1 (strongly disagree) to 5 (strongly agree)
Implicit Relational Assessment Procedure (IRAP)	2014	Self-Report	No Subscales	24	"When times are really difficult, I tend to be tough on myself" "When something painful happens, I try to take a balanced view of the situation"	7-point Likert-type scale ranging from 1 (completely false) to 7 (completely true)
Impulsiveness-Venturesomeness-Empathy Questionnaire [Unnamed]	1978	Self-Report	(impulsiveness) (venturesomeness) (empathy)	63	"Do you often get emotionally involved with your friends' problems" "Do unhappy people who are sorry for themselves irritate you"	yes/no

Instrument	Published Year	Type of Measure	Subscales/ Forms	Total Number of Items	Sample Item(s)	Response set
Index of Empathetic Concern	1972	Self-Report	(positive adjectives) (negative adjectives)	13	"It makes me sad to see a lonely stranger in a group" "I tend to get emotionally involved in a friend's problems"	Likert-type scale
Index of Responding	1984	Self-Report	(negative affect factor) (contrasting affect factor)	8		
Insanity Defense Attitude - Revised (IDA-R) Scale	2004	Self-Report	(strict liability) (injustice and danger)	19	"We should punish people who commit criminal acts, regardless of their degree of mental disturbance" "Some people with severe mental illness are out of touch with reality and do not understand that their acts are wrong. These people cannot be blamed and do not deserve to be punished"	7-point Likert-type scale ranging from 1 (strongly disagree) to 7 (strongly agree)
Integrity Scale	2008	Self-Report	No Subscales	18		1 = strongly disagree; 7= strongly agree
Interaction with Disabled Persons Scale (IDP)	1992	Self-Report	(discomfort) (sympathy)	20	"I feel uncomfortable and find it hard to relax." "I feel frusturated because I don't know how to help."	5-point scale ranging from "Stromgly Agree" to "Strongly Disagree"
Interconnectedness Scale	2020	Self-Report	(well-being) (mindfulness) (nonattachment)	25	When I hear about someone (a stranger) going through a difficult time, I feel a great deal of compassion for him or her	7-point Likert scale from 1 (not at all true of me) to 7 (very true of me)
Intercultural Effectiveness Scale (IES)	1991	Self-Report	(communication competence) (personality traits) (cultural empathy) (communication behavior)	20		
International Personality Item Pool (IPIP)	1999	Self-Report	(benevolence) (entitlement)	8		
Interpersonal and Social Empathy Index (ISEI)	2013	Self-Report	(macro perspective-taking) (cognitive empathy) (self–other awareness) (affective response)	32	"I am good at understanding other people's emotions" "When I am with someone who gets a bad news, I feel sad for a moment too"	6-point Likert-type scale that ranged from never (1) to always (6)
Interpersonal Communication Assessment Scale (ICAS)	2006	Self-Report	(advocacy) (therapeutic use of self) (validation)	23	"Acknowledges concerns" "Observes for needed emotional support"	Likert-type scale (1 = seldom; 2 = often; 3 = usually; 4 = always)
Interpersonal Communication Competence Scale (ICCS)	1994	Self-Report	(self-disclosure) (empathy) (social relaxation) (assertiveness) (interaction management) (altercentrism) (expressiveness) (supportiveness) (immediacy) (environmental control)	30	"How often do you have difficulties understanding other's feelings" "How often are you able to put yourself in others' shoes"	5-point Likert-scale (almost never = 1, seldom = 2, sometimes = 3, often = 4, almost always = 5)
Interpersonal Guilt Rating Scale –15 Self-Report (IGRS-15s)	2018	Self-Report	(survivor guilt) (omnipotent guilt) (self-hate)	15	"When someone is feeling down, I can usually understand how they feel" "I get caught up in other people's feelings easily"	5-point Likert-type scale ranging from 1 (strongly disagree) to 5 (strongly agree)
Interpersonal Quality of Family Planning Scale (IQFP)	2018	Peer/Corollary Report	No Subscales	11	"Respecting me as a person" "Showing care and compassion"	5-point Likert scale ranging from poor to excellent
Interpersonal Reactivity Index - Brief Form (B–IRI)	1980	Self-Report	(fantasy) (perspective taking) (empathic concern) (personal distress)	16	"I tend to lose control during emergencies" "When I am reading an interesting story or novel, I imagine how I would feel if the events in the story were happening to me"	5-point Likert-type scale ranging from 0 (does not describe me well) to 4 (describes me very well)
Interpersonal Reactivity Index (IRI)	1980	Self-Report	(perspective taking) (fantasy) (empathetic concern) (personal distress)	28	"I tend to lose control during emergencies" "When I am reading an interesting story or novel, I imagine how I would feel if the events in the story were happening to me"	5-point Likert-type scale ranging from 0 (does not describe me well) to 4 (describes me very well)
Interpersonal Regulation Questionnaire (IRQ)	2018	Self-Report	(negative tendency) (negative efficacy) (positive tendency) (positive efficacy)	16	"When I'm having trouble, I can't wait to tell someone about it" "I appreciate having others' support through difficult times"	7-point Likert-type scale ranging from 1 (strongly disagree) to 7 (strongly agree)
Interview Satisfaction Questionnaire (ISQ)	2011	Self-Report	(opportunity to disclose concerns) (physician's empathy) (confidence in physician's abilities) (general satisfaction)	12	"The phsyician gave me undivided attention" "The physician tried to make me feel relaxed"	5-point Likert-type scale ranging from 1 (strongly disagree) to 5 (strongly agree)
Inventory of Beliefs about Wife Beating (IBWB)	1987	Self-Report	(wife beating is justified) (wives gain from beatings) (help should be given) (offender should be punished) (offender is responsible)	36	"Social agencies should do more to help battered women" "There is no excuse for a man beating his wife."	7-point Likert-type scale ranging from 1 (strongly disagree) to 7 (strongly agree)
Inventory of Callous-Unemotional (ICU) Parent-Report Form	2004	Peer/Corollary Report	(callous-unemotional) (empathic-prosocial) (uncaring factors)	24	"I express my feelings openly" " I feel bad or guilt when I do something wrong"	4-point Likert scale, ranging from 0 (Not at all true) to 3 (Definitely True)
Inventory of Callous-Unemotional Traits (ICU)	2004	Peer/Corollary Report	(empathic-prosocial) (callous-unemotional)	24	"I express my feelings openly" "I feel bad or guilt when I do something wrong"	4-point Likert scale, ranging from 0 (Not at all true) to 3 (Definitely True)
Inventory of Cross-Cultural Sensitivity (ICCS)	1986	Self-Report	No Subscales	32	"I speak only one language" "The way other people express themselves is very interesting to me"	1 Strongly Agree to 7 - Strongly Disagree
Inventory of Motivations for Palliative Care Volunteerism (IMPCV)	2004	Self-Report	(altruism) (civic responsibility) (self-promotion) (leisure) (personal gain)	25	"It is my responsibility to help others" "I want to make others happy and comfortable in life, as well as in death"	6-point scale from 0 (does not describe me well) to 5 (describes me very well)

Instrument	Published Year	Type of Measure	Subscales/ Forms	Total Number of Items	Sample Item(s)	Response set
Inventory of Personality Organization (IPO)	2001	Self-Report	(Psychological Defenses) (Identity Diffusion) (Reality Testing)	155		
Jefferson Scale fof Physician Empathy - Student Version (JSPE-S)	2001	Peer/Corollary Report	(perspective taking) (compassionate care) (standing in the patient'sshoes)	20	"It is difficult for me to view things from my patients' perspective" "Patients feel better when their health care providers understand their feelings."	7-point Likert-type scale ranging from 1 (strongly disagree) to 7 (strongly agree)
Jefferson Scale for Physician Empathy for Observers (JSEO)	2016	Behavioural/Expert Coder	No Subscales	5	"Can view things from the patient's perspective (see things as the patient would)" "Asks about what is happening in the patient's daily life"	7-point scale (1 = Strongly Disagree, 7 = Strongly Agree)
Jefferson Scale of Attitudes toward Physician–Nurse Collaboration (JSAPNC)	2001	Self-Report	(shared education and collaboration) (physician–nurse collaboration) (physician's authority)	15	"It is difficult for me to view things from my patients' perspective" "Patients feel better when their health care providers understand their feelings."	7-point Likert-type scale ranging from 1 (strongly disagree) to 7 (strongly agree)
Jefferson Scale of Empathy - Health Profession Students (JSE-HPS)	2016	Self-Report	No Subscales	20	"It is difficult for me to view things from my patients' perspective" "Patients feel better when their health care providers understand their feelings."	7-point Likert-type scale ranging from 1 (strongly disagree) to 7 (strongly agree)
Jefferson Scale of Empathy - Law Students (JSE-LS)	2001	Self-Report	(understanding the client's perspective) (responding to clients' experiences and emotions) (responding to clients' cues and behaviors) (standing in the client's shoes)	20	"It is difficult for me to view things from my patients' perspective" "Patients feel better when their health care providers understand their feelings."	7-point Likert-type scale ranging from 1 (strongly disagree) to 7 (strongly agree)
Jefferson Scale of Empathy - Medical Student Version (JSE-MS)	2002	Self-Report	No Subscales	20	"It is difficult for me to view things from my patients' perspective" "Patients feel better when their health care providers understand their feelings."	7-point Likert-type scale ranging from 1 (strongly disagree) to 7 (strongly agree)
Jefferson Scale of Empathy (JSE)	2001	Self-Report	(perspective taking) (compassionate care) (standing in the patient's shoes)	20	"It is difficult for me to view things from my patients' perspective" "Patients feel better when their health care providers understand their feelings."	7-point Likert-type scale ranging from 1 (strongly disagree) to 7 (strongly agree)
Jefferson Scale of Empathy for Observers (JSEO)	2017	Peer/Corollary Report	No Subscales	5	"It is difficult for me to view things from my patients' perspective" "Patients feel better when their health care providers understand their feelings."	7-point Likert-type scale ranging from 1 (strongly disagree) to 7 (strongly agree)
Jefferson Scale of Patient's Perception of Physician Empathy (JSPPPE)	2007	Self-Report	No Subscales	5	"The doctor…(1) can view things from my perspective (see things as I see them)" "The doctor…(2) asks about what is happening in my daily life."	7-point Likert scale, with responses ranging from 1 (strongly disagree) to 7 (strongly agree)
Jefferson Scale of Physician Empathy (JSPE)	2001	Self-Report	(perspective taking) (compassionate care) (standing in patient's shoes)	20	"It is difficult for me to view things from my patients' perspective" "Patients feel better when their health care providers understand their feelings."	7-point Likert-type scale ranging from 1 (strongly disagree) to 7 (strongly agree)
Kids' Empathic Development Scale (KEDS)	2012	Behavioural/Expert Coder	(cognitive) (affective) (behavioral)	13		
Kiersma-Chen Empathy Scale (KCES)	2013	Self-Report	No Subscales	15	"Health care providers should try to stand in their patients' shoes when providing care to them" "Patients feel better when their health care provider understand their feelings"	7-point Likert scale from 1 (strongly disagree) to 7 (strongly agree)
Kiersma-Chen Empathy Scale Patient Scale (KCES-PV)	2013	Peer/Corollary Report	(Global empathy belief) (Health care professional's empathy ability)	14		8 response categories (Part 1: 1 = unnecessary, 4 = moderately necessasry, 7 = extremely necessary; Part 2: 1 = does not describe me, 4=describes me moderately well, 7 = describes me extremely well)
Kiersma-Chen Empathy Scale Revised Version (KCES-R)	2013	Self-Report	(Global empathy belief) (Personal empathy ability)	14		7 response categories (Part 1: 1 = unnecessary, 4 = moderately necessasry, 7 = extremely necessary; Part 2: 1 = does not describe them, 4=describes themmoderately well, 7 = describes themextremely well)
Kindness Scale	2021	Self-Report	(Affective-Socially Prescribed) (Affective-Proactive) (Principle-Socially Prescribed) (Principle-Proactive) (Anthropophilia)	45	"I open doors to let people through" "I think it is right to give everyone a fair chance"	
Kirk Adoption Questionnaire (KAQ)	1981	Self-Report	(AOD) (Empathy) (Communication)	14	"How frequently have you wondered whether the birth mother ever thinks about the child?" "How frequently have you recalled at one time the child legally belonged to someone else?"	4-point Likert scale (1 = never, 2 = infrequently, 3 = sometimes, 4 = often)
Knee Osteoarthritis Expectations Questionnaire	2011	Self-Report	(expectations for education) (expectations for information) (expectations for physician empathy)	60	"I expect that my phsycian takes time to listen to me" "Takes into consideration all the problems I have in my daily living activities because of my knee"	11-point Likert scales from 0 (disagree) to 10 (agree)

Instrument	Published Year	Type of Measure	Subscales/ Forms	Total Number of Items	Sample Item(s)	Response set
Leeds Satisfaction Questionnaire	1992	Self-Report	(overall satisfaction) (information) (empathy) (technical competence) (attitude) (access and continuity)	58	"The seats in the waiting area are very comfortable" "There are always a lot of people attending the clinic"	Strongly agree to Strongly disagree
Level of Personality Functionin Scale - Brief Form 2.0 (LPFS-BF 2.0)	2016	Self-Report	(Self) (Interpersonal)	12		
Level of Personality Functioning Scale - Self Report (LPFS-SR)	2017	Self-Report	(Identity) (Self-Direction) (Empathy) (Intimacy)	80		
Level of Personality Functioning Scale (LPFS)	2016	Self-Report	(identity) (self-direction) (empathy) (intimacy)	80	"I don't have a clue about why other people do what they do" Many people around me have very destructive motives"	4-point scale ranging from Totally False, not at all True to Very True
Levenson Self-Report Psychopathy Scale	1995	Self-Report	(callousness) (antisocial) (egocentricity)	26	"I make a point of trying not to hurt others in pursuit of my goals" "I feel bad if my words or actions cause someone else to feel emotional pain"	4-point Likert-type scale ranging from 1 (strongly disagree) to 4 (strongly agree)
Limited Prosocial Emotions Questionnaire (LPEQ)	2020	Self-Report	No Subscales	6	"Lacks guilt after he or she misbehaves or hurts someone" "Lacks empathy for or caring about others who are hurt, sad, or distressed"	4-point Likert-type scale ranging from 1 (not at all like this) to 4 (very much like this)
Machiavellian Personality Scale (MPS)	2009	Self-Report	(distrust of others) (desire for status) (desire for control) (amorality)	16	"I feel others needs" "I have a good word for everyone"	a five-point Likert scale (1 = strongly disagree, 2 = disagree, 3 = neither agree nor disagree, 4 = agree, 5 = strongly agree).
Marital Communication Inventory (MCI)	1970	Self-Report	(regard) (self-disclosure anxiety) (aversive communication) (empathy) (discuss)	46	"My spouse understands my words but not my feelings" "My spouse does not realize how strongly I feel about things"	9-point Likert-type scale
Measure of Caring in Nursing as Communicative Action [Unnamed]	1995	Self-Report	(caring identity) (patient focus scale) (professionalism) (nurse-patient interaction) (trusting partnership) (communicative mutuality) (responsibilities of care) (nursing practice scale)	102	"To me, patients are more than illness problems" "My own feelings give me insights into my patients"	6-category rating scale extending from Very Strongly Agree to Very Strongly Disagree
Measure of Empathy in Early Childhood (MEEC)	2023	Peer/Corollary Report	(Attention to Others' Emotions) (Personal Distress (i.e., Emotional Contagion/Affective Empathy)) (Personal Distress–Fictional Characters) (Prosocial Behavior) (Sympathy)	35	"My child comforts me when I am upset" "When I am hurt, my child comes over and checks on me"	4-point response scale from 1 (strongly disagree) to 4 (strongly agree)
Measure of Expressed Empathy	2002	Behavioural/Expert Coder	No Subscales	20		
Measure of Orientations Toward Empathic and Sympathetic Care	2010	Self-Report	(empathic response) (sympathetic involvement)	4	"I often have tender, concerned feelings for people less fortunate than me" "I tend to lose control during an emergency"	five-point Likert-like scale (0 = Does not describe me well; 4 = Describes me very well)
Measure of Relational Climate [Unnamed]	2020	Self-Report	(shared vision) (compassion) (relational energy)	12	"Members of my organization are empathetic toward each other" "People in my organization notice when others are in need"	five-point Likert scale ranging from 1 = very weak fit to 5 = very strong fit
Medical Condition Regard Scale (MCRS)	2002	Self-Report	(positive regard) (negative regard)	11	"I feel especially compassionate toward patients like this" "Patients like this irritate me"	6-point Likert scale (1 = strongly disagree, 6 = strongly agree)
Medical Humanities Questionnaire	2011	Self-Report	(professionalism) (caring and empathy) (patient orientation) (disclosure of harm) (communication)	19		
Mental States Task (MST)	2013	Self-Report	(concrete thinking) (low defensive level) (intermediate defensive level) (objective-rational) (high defensive level) (reflective thinking)	24	"I thought that what the character was going through was not that bad" "The character's situation moved me, but I was not overwhelmed with sadness"	7-point Likert-type scale ranging from 1 (strongly disagree) to 7 (strongly agree)
Mentalized Affectivity Scale (MAS)	2017	Self-Report	(identifying emotions) (processing emotions) (expressing emotions)	60	"If I feel something, I will convey it to others" "It is hard for me to talk about my complex emotions"	four point scale (strongly disagree, slightly disagree, slightly agree, or strongly agree)
Military Suicide Attitudes Questionnaire Short-Form (MSAQ-SF)	2022	Self-Report	(discomfort) (unacceptability) (support) (empathic views)	18	"A service member who attempts suicide deserves understanding and empathy." "People who attempt suicide should be given time to receive help."	5-point Likert scale (1 = strongly disagree to 5 = strongly agree)
Millennial Organizational Citizenship Behavior (OCB) Scale	2021	Self-Report	(Empathetic Responsiveness) (Professional Development) (Role Modeling) (Social Responsibility)	13	"I empathetically respond to my coworkers' feelings" "I show sensitvity to my coworkers' emotional condition"	seven-point Likert scale ranging from 1 (strongly disagree) to 7 (strongly agree)
Mindful Attention Awareness Scale (MAAS)	2003	Self-Report	No Subscales	15	I find it difficult to stay focused on waht is happening in the present	6-point Likert scale ranging from 1 (almost always) to 6 (almost never)

Instrument	Published Year	Type of Measure	Subscales/ Forms	Total Number of Items	Sample Item(s)	Response set
Mindful Parenting Inventory for Children (MPIC)	2023	Self-Report	(Self-Regulation in Parenting) (Acceptance and Compassion towards Child) (Being in the Moment with Child) (Awareness of Child)	18	NA	5-point Likert scale (never true = 1 to always true = 5)
Mindful Parenting Inventory for Parents (MPIP)	2023	Self-Report	(Self-Regulation in Parenting) (Acceptance and Compassion towards Child) (Being in the Moment with Child) (Awareness of Child)	18	null	5-point Likert scale (never true = 1 to always true = 5)
Mindful Self-Care Scale (MSCS)	2017	Self-Report	(physical care) (supportive relationships) (mindful awareness) (self-compassion and purpose) (mindful relaxation) (supportive structure)	84	"Reminded myself that failure and challenges are a part of the human experience"<br>"Engaged in supportive and comforting self-talk"	5 point Likert-type response anchors consisting of “strongly dislike,” “slightly dislike,” “neutral,” “slightly like,” or “strongly like,”
Minnesota Multiphasic Personality Inventory-2 (MMPI-2)	1943	Self-Report	(hypochondriasis) (depression) (conversion hysteria) (psychopathic deviate) (masculinity/femininity) (paranoia) (psychasthenia) (schizophrenia) (hypomania) (social introversion)	567	“A sad movie makes me feel like crying”	7 point Likert-type response anchors consisting of (1) Never, (2) Very rarely, (3) Rarely, (4) Occasionally, (5) Frequently, (6) Very frequently, and (7) Always
Miville-Guzman Universality-Diversity Scale	1987	Self-Report	(relativistic appreciation of oneself and others) (diversity of contact) (sense of connection)	45	"I often have tender, concerned feelings for people less fortunate than me"<br>"Sometimes I don't feel very sorry for other people when they are having problems"	6 point Likert-type scale
Modified Version of Empathic Accuracy Task (EAT)	2009	Self-Report	No Subscales	27	picture items	rate the perceived emotional intensity of each target using a 9-point scale from 1 (no emotion) to 9 (very strong emotion)
Moral Courage Scale for Physicians (MCSP)	2006	Self-Report	No Subscales	12	"It is difficult for me to view things from my patients' perspective"<br>"Patients feel better when their health care providers understand their feelings."	7-point Likert-type scale ranging from 1 (strongly disagree) to 7 (strongly agree)
Moral Expansiveness Scale (MES)	2016	Self-Report	No Subscales	30	“I often have tender, concerned feelings for people less fortunate than me”<br>"Sometimes I don't feel very sorry for other people when they are having problems"	5 point Likert scale from 1┐does not describe me well to 5┐describes me very well
Moral Foundations Questionnaire - Revised (MFQ-r)	2021	Self-Report	(Traditionalism) (Compassion) (Liberty)	41	"I think everyone should be free to do as they choose, so long as they don’t infringe upon the equal freedoms of others"<br>"I think it’s morally wrong that rich children inherit a lot of money while poor children inherit nothing"	6-point Likert scale
Moral Foundations Questionnaire (MFQ)	2011	Self-Report	(harm) (fairness) (ingroup) (authority) (purity)	30	"Compassion for those who are suffering is the most crucial virtue"<br>"One of the worst things a person could do is hurt a defenseless animal"	6-point scale from 1 (strongly disagree) to 6 (strongly agree)
Moral Inclusion/Exclusion of Other Groups Scale (MIEG)	2016	Self-Report	No Subscales	4	"Values held by this group represent a threat to our well-being"<br>"Members of this group deserve no respect "	7-point scale from 1 = ‘strongly disagree’ to 7 = ‘strongly agree’
Moral Mosaic - Short Form	1988	Self-Report	(psychological adjust) (social activity) (physical adjustment)	65	"Encouraged you to talk about your emotional feelings about your illness, encouraged you to talk about your illness experience"<br>"Told you not to worry as everything would be all right, told jokes and chattered to keep your mind off your illness"	scale from 0 (never) to 3 (often)
Multi-Dimensional Support Scale (MDSS)	1988	Self-Report	(psychological adjust) (social activity) (physical adjustment)	65	"Encouraged you to talk about your emotional feelings about your illness, encouraged you to talk about your illness experience"<br>"Told you not to worry as everything would be all right, told iokes and chattered to keep your mind off your illness"	scale from 0 (never) to 3 (often)
Multicultural Personality Questionnaire (MPQ)	1985	Self-Report	(cultural empathy) (open-mindedness) (emotional stability) (social initiative and flexibility)	78	"Pays attention to the emotions of other"<br>"Keeps calm when things don’t go well"	5-point Likert-type scale ranging from 1 (not at all applicable) to 5 (totally applicable)
Multicultural Personality Questionnaire Short Form (MPQ-SF)	2000	Self-Report	(Cultural Empathy) (Emotional Stability) (Flexibility) (Openmindedness) (Social Initiative)	40		7-point Likert scale (1=not at all applicable; 7=completely applicable)
Multifaceted Empathy Test (MET)	2008	Self-Report	(emotional empathy) (cognitive empathy)	82	“How aroused does this picture make you feel?”<br>“How strong is the emotion you feel about this person?”	9-point Likert-type scale ranging from 1 (minimum evels of arousal (implicit empathy) and empathic concern (explicit empathy)) and 9 (maximum levels of arousal (implicit empathy) and empathic concern (explicit empathy))

Instrument	Published Year	Type of Measure	Subscales/ Forms	Total Number of Items	Sample Item(s)	Response set
Multiple Mini-Interview (MMI)	2004	Behavioural/Expert Coder	(moral and ethical values) (interpersonal ability) (academic ability) (communication skills) (critical thinking skills)	21	"Kindness, compassion and empathy" "Respect for difference and diversity"	five or seven point Likert-type scale as well as assigning a global rating score which can range from 'excellent' to 'cause for concern'
My Emotions Questionnaire	2004	Self-Report	(amusement) (anxiety) (frustration) (sympathy) (protective)	20	"I feel sorry for this baby" "I feel concerned for this baby"	5-point Likert scale (1 = very weakly, 2 = weakly, 3 = moderately, 4 = strongly, 5 = very strongly)
Narnian Character Virtue Scales (NCVS)	2017	Self-Report	No Subscales	60	"Do you often get emotionally involved with your friends' problems" "Do you often long for excitement"	yes/no
Non-Verbal Compassionate Communication Questionnaire	2012	Self-Report	No Subscales	24	"How do you express compassion towards patients" "How do you show your anger towards patients"	different answer choices specific to each question
Nurse 5-item Compassion Measure	2019	Peer/Corollary Report	No Subscales	5	"During this hospital stay, how often do you feel your nurses cared about your emotional or psychological well-being?" "During this hospital stay, how often do you feel your nurses were interested in you as a whole person?"	1 = Never; 2 = Sometimes; 3 = Usually; 4 = Always
Nurse-Nursing Assistant-Caregiver Reciprocity Scale (NNA-CRS)	2013	Self-Report	(balanced reciprocal ethical caring team work) (love and affection) (intrinsic rewards)	16		
Nurses' Confidence Scale: Unique Families (NCSUF)	2020	Self-Report	(skills and resources) (awareness and sensitivity)	19	"I can provide social support (e.g., information, emotional, practical, affirmation) for all unique families under our care" "I can identify and manage my internal conflict when caring for families whose lifestyle choices differ from my own"	5-point Likert scale (1 = not confident at all to 5 = very confident)
Nursing Dimensions Inventory (NDI)	1999	Self-Report	No Subscales	35	"Getting to know the patient as a person" "Feeling sorry for a patient"	5-point Likert-type scale ranging from 1 (strongly disagree) to 5 (strongly agree)
Nursing Motives for Helping Scale (N-MHS)	2006	Self-Report	No Subscales	9		Likert scale, where lower scores indicate lesser empathy and altruistic traits in the respondents
Nursing Shared Decision-Making Attitude Scale (NSDMA)	2021	Self-Report	(Empathic communication) (Mastery learning)	11	"Eager to respect and empathize with patients' preferences and values." "Eager to implement patient-centered care."	5-point Likert scale, ranging from 1 (strongly disagree) to 5 (strongly agree)
Objective Structured Clinical Evaluation (OSCE)	1979	Peer/Corollary Report	(empathy) (coherence) (verbal and non-verbal expression)	10		
Observed Mindfulness Measure (OMM)	2022	Peer/Corollary Report	(mindful awareness) (acceptance) (attentiveness)	9	Please tell us how frequently each of the following statements would be true about your paired participant	1: "Not at all", 2: "Occasionally", 3: "About half the time", 4: "Frequently", 5: "All the time"
Open-Minded Cognition Scale [Unnamed]	2015	Self-Report	(general) (political) (religious) (open-minded cognition)	18	"I can strongly feel what other people feel" "I care a great deal of what happens to people	7-point Likert scale from 1 (strongly disagree) to 7 (strongly agree)
Orientation Model	2015	Self-Report	(cognitive processing) (attachment) (empathy) (introspection)	10	"When I'm upset at someone, I usually try to 'put myself in their shoes' for a while" "I often have tender, concerned feelings for people less fortunate than me"	5-point Likert-type scale ranging from 1 = not very true of me to 5 = very true of me
OSPA Inventory	1935	Self-Report	(purpose) (sodalness) (sympathy) (poise) (impulse-judgment)		"Did you ever cry over a book or a story" "When you see other people sad does it usually make you sad"	yes/no
Other Dyadic Perspective-Taking Scale (ODPT)	1990	Self-Report	(strategies) (cognizance)	23	"In my relationship with my partner I believe that there are two sides to every question, and I try to look and think about both sides." "When involved in an argument with me, my partner is the type of person who will consider and take into account my point of view and compare that with his/her own."	5-point Likert-type scale ranging from 0 (totally disagree) to 4 (totally agree)
Parent Affective and Cognitive Empathy Scale (PACES)	1998	Behavioural/Expert Coder	(affective empathy) (cognitive empathy) (empathic behavior)	23	"[Child] was happy, so I started to feel happy, too." "When [child] told me what her friend had said, I felt angry on her behalf."	11-point scale ranging from -1 (anti-empathic, hostile, rejecting) to 9 (exceptional, insightful)
Parental Empathy Measure (PEM)	2005	Self-Report	(attention to signals subscale) (behavior-unstressed subscale) (behavior-stressed subscale) (emotion-unstressed subscale) (emotion-stressed subscale) (atrributions subscale) (faking good scale)	24		
Partner Synchrony Inventory (PSI)	1997	Self-Report	(authoritativeness) (empathy) (compliance)	15		

Instrument	Published Year	Type of Measure	Subscales/ Forms	Total Number of Items	Sample Item(s)	Response set
Patient Centeredness Index (PCI)	2022	Peer/Corollary Report	No Subscales	14	"They pay attention to my needs." "They talk to me with respect."	0 = Never, 1 = Rarely, 2 = Sometimes, 3 = Usually, 4 = Always, N/A
Patient Experience Survey in Radiation Therapy	2019	Self-Report	No Subscales	3	"My overall experience in Radiation Therapy was great"	5 point Likert-type scale
Patient Satisfaction Scale	2002	Self-Report	(interpersonal) (logistics) (technical)	25	"Friendly providers" "Providers who listen to me"	
Patient-Physician Relationship Scale - Physician (PPRS-Physician)	2020	Self-Report	(Interfering attributes) (Positive attributes) (Personal connection)	35	"Is grateful for my help" "Is allied with me"	7-item Likert scale that range from 0 to 6, in which the "Very Undesirable" score = 0, to "Very Desirable" = 6
Patient-Reported Compassion Measure [Unnamed]	2020	Self-Report	(Virtuous response) (Relational space) (Seeking to understand) (Relational communication) (Attending to needs) (Global Compassion)	54	"I felt that my Healthcare Providers were compassionate" "I felt that my Healthcare Providers were caring"	5-point Likert scale: strongly disagree, disagree, neutral, agree, strongly agree
Patient's Assessment of Quality Scale - Acute Care Version (PAQS-ACV)	1989	Peer/Corollary Report	No Subscales	45		
Peace Attitude Scale (PAS)	2021	Self-Report	(Sociopolitical) (Personal Well-Being) (Ease with Diversity) (Environmentalist Attitude) (Caring)	22	"I never will host a foreign family in my house" "If I bumped into an injured animal, I wouldn't hesitate to take care of it"	7-point Likert-type scale (never, almost never, rarely, sometimes, often, very often, always)
Pediatric-Hematogoloy Oncology Fellowship Training Skills Measure [Unnamed]	2017	Self-Report	(balancing the competing demands of fellowships) (caring for the dying patient) (depression and burnout) (responding to challenging relationships with patents) (humanism in medicine)	15	"When caring for a patient who is dying, I can show the care and compassion a family needs" "Following a patient's death, I process my grief with great difficulty, coping poorly"	
Peer Edmonton Empathy Recruitment Scale (PEERS)	2018	Self-Report	No Subscales	18	"I am empathic" "I am egotistical"	10-point response scale (1 = strongly disagree to 10 = strongly agree)
Peer Group Caring Interaction Scale (PGCIS)	1998	Peer/Corollary Report	(caring behaviors) (giving assistance)	16	"Anticipate, and are well aware of, the wishes of classmates" "Talk freely to each other about their concerns and insterests"	
Perceived Empathic and Social Self-Efficacy Scale (PESE)	2010	Self-Report	(perceived empathic self-efficacy) (perceived social self-efficacy)	11	"How well can you read your friends' needs" "How well can you express your opinion to people who are talking about something of interest to you"	five-point Likert-type scale (1 = not well at all to 5 = very well)
Perceived Empathy Measure	1996	Peer/Corollary Report	No Subscales	8	"This salesperson seemed to feel what I needed when we talked about this purchase" "This salesperson understands me as a person and my role in this organization."	a 5-point scale anchored by strongly agree (5) and strongly &sagree (1)
Performance of Empathic Expression Rating Scale (PEERS)	2017	Behavioural/Expert Coder	(verbal) (nonverbal) (global)	11		
Person-Centered Care Assessment Tool (P-CAT)	2010	Self-Report	(Extent of Personalising Care) (Amount of Organisational Support) (Degree of Environmental Accountability)	13	"We have formal team meetings to discuss patients' care." "I simply do not have the time to provide care based on the person's choice."	5-point Likert scale (1 = strongly disagree to 5 = strongly agree)
Person-Centred Caring: Participation [Unnamed]	2020	Self-Report	No Subscales	10	The people involved in my health care... "... gave me the information I needed to make decisions that were right for me" "... respected my right to choose the care and treatment I wanted"	Never' = 0, 'Almost never' = 1, 'Sometimes' = 2, 'Almost always' = 3, 'Always' = 5
Person-Centred Caring: Personalization [Unnamed]	2020	Self-Report	(Connecting) (Knowing)	12	The people in my health care... "...showed an interest in me as a person" "... took enough time to understand how my health affects me"	Never' = 0, 'Almost never' = 1, 'Sometimes' = 2, 'Almost always' = 3, 'Always' = 4
Person-Centred Caring: Responsiveness [Unnamed]	2020	Self-Report	(Being attentive) (Reciprocity)	10	The people involved in my health care... "... knew which other healthcare staff were involved in my health care" ".... gave me their full attention when they were with me"	Never' = 0, 'Almost never' = 1, 'Sometimes' = 2, 'Almost always' = 3, 'Always' = 6
Personal Orientation Inventory (POI)	1964	Self-Report	(self actualization) (belief in human benevolence) (modernity) (self consciousness) (empathy) (rigidity)	150	"I often get swept up in my friends' feelings" "I don't become sad when I see other people crying"	9-point scale
Personal Strengths Inventory (PSI)	2007	Self-Report	(social competence) (emotional awareness) (goal setting) (emotional regulation) (empathy)	22	"When I see someone being treated unfairly, I feel sympathy for them" "I feel sorry for people when they are having problems"	5-point scale, where 1 = very seldom true of me, 2 = seldom true of me, 3 = sometimes true of me, 4 = often true of me, and 5 = very often true of me
Personal Trust and Connections scale (PerTC)	2022	Self-Report	(Emotional Reliance) (Social Reliance) (Cognitive Reliance)	10	"Can you trust persons you have met for a few times?" "Do you rapidly feel close to persons you trust?"	10-point Likert-type scale (from 0: not at all, to 10: very much)
Personality Assessment Inventory-Borderline Features Scale (PAI-BOR)	1991	Self-Report	(Affective Instability) (Identity Problems) (Negative Relationships) (Self-Harm)	24		

Instrument	Published Year	Type of Measure	Subscales/ Forms	Total Number of Items	Sample Item(s)	Response set
Personality Functioning Scale - Self Report (LPFS-SR)	2017	Self-Report	(identity) (self-direction) (intimacy) (empathy)	80	"I see myself as someone who is helpful and unselfish with others"<br>"I see myself as someone who can be cold and aloof"	5-point scale ranging from disagree strongly to agree strongly
Perth Empathy Scale (PES)	2023	Self-Report	(cognitive empathy) (positively valenced) (negatively valenced)	20		5-point Likert scale, ranging from almost never (1) to almost always (5)
Pharmacists' Care of Migraineurs Scale (PCMS)	2008	Self-Report	(empathy) (prospective drug utilization review) (headache sufferer triage) (medication counseling) (nonpharmacologic treatment plan) (dissemination of public health information) (maintenance of knowledge on migraine)	45	"I listen attentively to patients descrbe frustrations regarding their headaches"<br>"I provide empathic responses to persons complaining of migraine or other recurring headaches"	1 = never to very rarely, 2 = rarely, 3 = sometimes, 4 = fairly often, and 5 = nearly all the time to all the time
Phenomenological Body Shame Scale - Revised (PBSS-R)	1998	Self-Report	No Subscales	8	For the next set of statements, imagine that you are looking at your body in a mirror, and indicate the degree to which you experience ach of the feelings described, using the scale provided: "I feel like covering my body"<br>"I feel angry with myself"	5-point scale ranging from 1 (not at all) to 5 (extremely)
Physician 5-item Compassion Measure	2019	Peer/Corollary Report	No Subscales	5	"During this hospital stay, how often do you feel your doctors cared about your emotional or psychological well-being?"<br>"During this hospital stay, how often do you feel your doctors were interested in you as a whole person?"	1 = Never; 2 = Sometimes; 3 = Usually; 4 = Always
Physician-Caregiver Relationship Scales (PCRS)	2008	Behavioural/Expert Coder	(physician-caregiver liking) (physician-caregiver understanding) (physician dominance)	N/a		
Posttraumatic Growth Inventory (PTGI)	1996	Self-Report	(compassion) (new possibilities) (relating to others) (personal strength) (appreciation of life and spiritual change)	8	"I feel compassion for other people who are facing challenges"<br>"I try to encourage people when they feel low"	six-point Likert scale from 0 (not at all) to 5 (very great degree)
Pregnant Women with HIV Attitude Scale	2010	Self-Report	(child care) (myths/negative stereotypes) (reproduction/contraceptive issues) (sympathy/transmission routes)	27	"Volunteer to have their babies tested"<br>"Sympathy blood transfusions vs. injection drug use"	5-point Likert scale ranging from strongly agree to strongly disagree
Prejudice Confrontation Styles (PCS) Scale	2021	Self-Report	(Educational) (Argumentative) (Help-seeking) (Empathy) (Humor)	22	"I teach them the various ways discrimination is still prominent in our society"<br>"I let them know they have hurt me"	1 (not at all true of me) to 5 (very true of me)
Prejudice toward People with Borderline Personality Disorder (PPBPD) Scale	2018	Self-Report	(Fear/Avoidance) (Malevolence) (Authoritarianism) (Unpredictability)	28	"I am not scared of people with borderline personality disorder."<br>"People who have borderline personality disorder should be forced to have treatment"	9-point Likert-type scale, ranging from 1 (very strongly disagree) to 9 (very strongly agree)
Prejudice toward People with Depression (PPD)	2018	Self-Report	(fear/avoidance) (unpredictability) (authoritarianism) (malevolence)	28	"I would find it hard to talk to someone who has depression"<br>"I would be just as happy to invite a person with depression into my home as I would anyone else"	9-point Likert-type scale, ranging from 1 (very strongly disagree) to 9 (very strongly agree)
Prejudice toward People with Schizophrenia (PPS)	2018	Self-Report	(fear/avoidance) (unpredictability) (authoritarianism) (malevolence)	28	"I would find it hard to talk to someone who has schizophrenia"<br>"I would be just as happy to invite a person with schizophrenia into my home as I would anyone else"	9-point Likert-type scale, ranging from 1 (very strongly disagree) to 9 (very strongly agree)
Princess Margaret Hospital Patient Satisfaction with Doctor Questionnaire (PMH/PSQ-MD)	1999	Self-Report	(understanding the patient's situation) (can talk about personal things) (consider needs) (interest in patient) (assessment -look into problems) (treatment) (competence) (explain - give information) (patient role in communication) (patient understanding) (time issue) (respectful/caring) (social/family well-being)	29	"The doctor did not seem to really consider my needs"<br>"I felt able to tell this doctor about very personal things"	four-point Likert scale (strongly agree, agree, disagree, and strongly disagree) as well as "does not apply" and "statement not clear"
Professional Quality of Life - Revised (ProQOL-21)	2010	Self-Report	(Compassion Satisfaction) (Compassion fatigue)	21	NA	three-to-five point Likert scales, depending on the item
Professional Quality of Life Scale - Version 5 (ProQoL 5)	2010	Self-Report	(compassion satisfaction) (secondary traumatic stress) (burnout)	30	"Over the past six months, my work made me feel enthusiastic"<br>"Over the past six months, my work made me feel content"	5-point Likert scale ranging from 1 (never) to 5 (almost always)
Professional Quality of Life Scale (ProQoL)	2010	Self-Report	(risk for burnout) (compassion satisfaction) (secondary traumatic stress)	30	"Over the past six months, my work made me feel enthusiastic"<br>"Over the past six months, my work made me feel content"	5-point Likert scale ranging from 1 (never) to 5 (almost always)

Instrument	Published Year	Type of Measure	Subscales/ Forms	Total Number of Items	Sample Item(s)	Response set
Professionalism Assessment Scale (PAS)	2013	Self-Report	(professional relationship and development) (responsibility) (empathy and humanism)	22	"Physician should do his/her best to help the patient in every consultation" "Physicians should adapt to the level of the patient's understanding"	5-point Likert scale ranging from strongly agree to strongly disagree
Prosocial and Antisocial Behavior in Sport Scale (PABSS)	2009	Self-Report	(prosocial - toward opponents) (prosocial - toward teammates) (antisocial - toward opponents) (antisocial - toward teammates)	20	"Helped an injured opponent" "Deliberately fouled an opponent"	5-point Likert scale ranging from never to very often
Prosocial Personality Battery (PSP)	1995	Self-Report	(autonomy oriented helping) (dependency oriented helping) (opposition to helping)	24	"I like to try to help people even if the issue might come up again" "Teaching people to take care of themselves is good for society because it makes them independent"	1 (strongly disagree) to 7 (strongly agree) scale
Prosocial Risk-Taking Scale (PSRT)	2021	Self-Report	No Subscales	6	"You and your classmates are getting into groups for a project. An unpopular student asks you to work with her. You say yes, even though you risk your classmates judging you as uncool too." "You notice some popular kids from school picking on another kid. You decide to step in and help the kid, even though you risk looking uncool or getting picked on yourself."	5-point Likert-type scale ranging from "1 = Very unlikely" to "5 = Very likely"
Psychopathic Personality Inventory - Short Form (PPI-SF)	1990	Self-Report	(Machiavellian Egocentricity) (Social Potency) (Coldheartedness) (Fearlessness) (Impulsive Nonconformity) (Blame Externalization) (Carefree Nonplanfulness) (Stress Immunity)	56	"I become deeply attached to people I like," Reversed	four-point Likert-type scale
Psychosocial Costs of Racism to Whites Scale (PCRW)	2004	Self-Report	(white empathic reactions toward racism) (white guilt) (white fear of others)	16	"I am angry that racism exists" "I become sad when I think about racial injustice"	6 point Likert-type scale from 1 (strongly disagree) to 6 (strongly agree)
Psychosocial Inventory of Ego Strengths (PIES)	1964	Self-Report	(hope) (will) (fidelity) (love) (care) (purpose) (competence) (wisdom)	128		
Public Attitudes Towards Homelessness (PATH)	1988	Self-Report	(homeless) (poverty) (achieving tendency) (just world) (authoritarianism) (need for approval)	38	"Society is responsible for people being homeless" "Many homeless people have themselves to blame"	4 point Likert-type scale from 1 (strongly disagree) to 4 (strongly agree)
Quality of Interactions Schedule (QuIS)	1993	Behavioural/Expert Coder	No Subscales	N/A		five-category scale: positive social, positive care, neutral, negative protective, negative restrictive
Questionnaire Measure of Empathic Tendency	1972	Self-Report	(susceptibility to emotional contagion) (appreciation of the feelings of unfamihar and distant others) (extreme emotional responsiveness) (tendency to be moved by others) (sympathetic tendency) (willingness to be in contact with others who have problems)	33	"It makes me sad to see a lonely stranger in a group." "The people around me have a great influence on my mood."	8-point Likert-type scale ranging from -4 (very strong disagreement) to 4 (very strong agreement)
Questionnaire of Cognitive and Affective Empathy (QCAE)	2010	Self-Report	(perspective taking) (online simulation) (emotion contagion) (proximal responsivity) (peripheral responsivity)	31	"It is hard for me to see why some things upset people so much" "I find it easy to put myself in somebody else's shoes"	4 point Likert-type scale from 1 (strongly disagree) to 4 (strongly agree)
Range and Differentiation of Emotional Experience Scale (RDEES)	2004	Self-Report	(range) (differentiation)	14	"I tend to experience a broad range of different feelings" "I am aware of the different nuances or subtleties of a given emotion"	5-point scale, with 1 indicating that it "does not describe me very well" and 5 indicating that it "describes me very well."
Rating Scales for the Assessment of Empathic Communication in Medical Internviews (REM)	2007	Self-Report	(empathic communication) (confrontation)	9	"Did the physician provide the opportunity for the patient to give his/her opinion?" "Did the physician treat the patient as an equal partner?"	7-point Likert scale
Reading the Mind in the Eyes Test (RMET)	1997	Self-Report	No Subscales	36	"I try to look at everybody's side of a disagreement before I make a decision" "It is easy for me to understand why others become sad when something heartbreaking happens to them"	5 point and 7 point Likert-type scales
Reflective Functioning Questionnaire	2015	Self-Report	(certainty about mental states) (uncertainty about mental states)	101	"I don't become sad when I see other people crying" "My friend's emotions don't affect me much"	6-point Likert-type scale ranging from 1 (completely disagree) to 6 (complete agree)

Instrument	Published Year	Type of Measure	Subscales/ Forms	Total Number of Items	Sample Item(s)	Response set
Relational Care Scale (RCS)	1998	Self-Report	No Subscales	6	"The care aide takes your likes and dislikes into account when he or she is providing care." "The care aide tries to meet your needs, for example, in such ways as listening to you if you need someone to talk to and/or comforting you when something bad or unexpected happens."	Five possible choices: 5 = always, 4 = often, 3 = occasionally, 2 = seldom, and 1 = never
Relational Competence for Community-Dwelling Elderly	1990	Self-Report	No Subscales	15		
Relational Depth (RD) Inventory	2011	Self-Report	(respect) (empathy & connectedness) (invigorated/liberating) (transcendence) (scared/vulnerable) (other person empathic/respectful)	54	"My therapist and I understand each other" "I feel my therapist cares about me even when I do things that he/she does not approve of"	5 point Likert-type scale
Relational Engagement of the Sacred for a Transgression (REST) Scale	2010	Self-Report	No Subscales	4	"Concerned" "Compassionate"	6-point Likert-type scale ranging from 0 (not at all) to 5 (extremely)
Relationship Inventory (RI)	1962	Self-Report	(empathy) (congruence) (regard)	69	"He/she usually understands all of what I say to her/him" "He/she understands my words but not the way I feel"	
Response Empathy Rating Scale	1982	Self-Report	(frame) (inference) (accuracy) (here &now) (centrality) (words) (voice) (manner) (impact)	9	"Does the counselor try to perceive the world as it appears to the client" "Is the counselor's voice expressive or empathic and approriate to what the client is expressing"	yes, definitely, perhaps, not sure, no, definitely
Restorative Justice Attitudes Scale (RJAS)	2021	Self-Report	(Empathic Understanding) (Harm and Needs) (Restoration Processes) (Accountability) (Community Engagement)	20	"It is important to empathize with individuals who have caused harm to others" "It is important to show emapthy toward offenders of wrongdoing"	5-point Likert-type scale, ranging from 1 (strongly disagee) to 5 (Strongly agree)
Revised Humane Caring Scale (RHCS)	1998	Self-Report	(relationships) (privacy) (communication) (respect) (promoting physical health) (conditions for humane caring during hospitalization)	46	"My fears are alleviated" "I was able to ask questions concerning my care"	5-point Likert-type scale ranging from 1 (totally disagree) to 5 (totally agree)
Revised Sense of Purpose Scale (SOPS-2)	2018	Self-Report	(Awareness of purpose) (Altruistic purpose) (Awakening to purpose)	14	"My purpose in life is clear" "I have a good understanding of my life's purpose"	1 = strongly disagree to 7 = strongly agree
Safety Skills Questionnaire [Unnamed]	2014	Self-Report	(honesty) (conscientiousness) (technical skills) (crisis management) (awareness of situation) (vigilance) (anticipation/preparedness) (awareness of self) (responsiveness) (common sense) (awareness of team) (organizational skills and efficiency) (confidence) (humility) (teamworking and communication) (awareness of patient) (leadership)	66		
San Diego Wisdom Scale (SD-WISE-7)	2019	Self-Report	(Decisiveness) (Emotional regulation) (Self-reflection) (Pro-social behaviors) (Social advising) (Acceptance of divergent perspectives)	7	"I remain calm under pressure" "I avoid self-reflection"	The items are rated from 1 or "strongly disagree" to 5 or "strongly agree"
San Diego Wisdom Scale (SD-WISE)	2017	Self-Report	No Subscales	24	"I am good at perceiving how others are feeling." "I have trouble making decisions."	Strongly disagree to "Strongly agree"
Santa Clara Brief Compassion Scale (SCBCS)	2005	Self-Report	No Subscales	5	"I tend to feel compassion for people, even though I do not know them" "When I hear about someone (a stranger) going through a difficult time, I feel a great deal of compassion for him/her	7-point scale from 1 being “not at all true of me” to 7 being “very true of me.”
Santa Clara Ethics Questionnaire (SCES)	2019	Self-Report	No Subscales	10	"I tend to feel compassion for people, even though I do not know them" "When I hear about someone (a stranger) going through a difficult time, I feel a great deal of compassion for him/her	7-point scale from 1 being “not at all true of me” to 7 being “very true of me.”
Satisfaction with Simulation Experience Scale (SSES)	2011	Self-Report	(becoming culturally competent) (learning from the debrief) (reflecting on practice)	18	"I believe the debrief has helped me be more understanding of the needs of culturally and linguistically diverse patients" "The facilitator made me feel comfortable and at ease during the debrief"	5-point Likert scale ranging from strongly agree to strongly disagree
Scale of Ethnocultural Empathy (SEE)	1983	Self-Report	(empathetic feeling and expression) (empathetic perspective taking) (acceptance of cultural differences) (empathetic awareness)	62	"When other people struggle with racial or ethnic oppression, I share their frustration" "I know what it feels like to be the only person of a certain race or ethnicity in a group of people"	6-point Likert scale from 1 (strongly disagree that it describes me) to 6 (strongly agree that it describes me)
Scale of Humanistic Nursing Behaviors	1987	Self-Report	(shared decision making) (holistic selves) (status equality) (empathy)	70	Staff include the patient in decisions concerning his or her care	5 point Likert scale that describes the frequency of occurence from 1 to 5

Instrument	Published Year	Type of Measure	Subscales/ Forms	Total Number of Items	Sample Item(s)	Response set
Scale of Teacher Empathy for African American Males (S-TEAAM)	2012	Self-Report	(teacher conceptions of empathy with black male students) (teacher application of empathy with black male students)	9	"I personally consider empathy to be a necessary disposition for teachers of Black students" "I personally consider empathy to be a necessary disposition for teachers of Black male students"	4 point scale from strongly disagree to strongly agree
Schadenfreude Scale	2012	Self-Report	No Subscales	12	"I take pleasure in another's failture" "I think it's funny when I see a person make a fool of himself or herself"	response scale from 1 (strongly disagree) to 9 (strongly agree)
Schema of Partner Empathic Responses to Anger (SOPERA)	1998	Self-Report	(consensus) (satisfaction) (cohesion) (affectional expression)	20		
School Support Scale	2006	Self-Report	(emotional support) (tangible support)	22	"I am encouraged to do my best" "I believe I am cared about at school"	7-point Likert scale format (1 =Never, 2 = Rarely, 3 = A Little, 4 = Sometimes, 5 = Regularly,6 = Often, and 7 = Always)
Schwartz Center Compassionate Care Scale (SCCCS)	1998	Peer/Corollary Report	No Subscales	12	"Showing care and compassion" "Express sensitivity, caring, and compassion for your situation"	5-points Likert scales ranging from “Poor” to “Excellent"
Screening Tool for Care Coordinator Assistants	2014	Peer/Corollary Report	(developing two relationships) (maintaining equanimity) (empathic response) (individual and caregiver education) (critical reasoning) (perceptions of an elderly person)	15		
Screening, Brief Intervention and Referral to Treatment Checklist for Observation in Real-time (SCORe)	2015	Behavioural/Expert Coder	(feedback) (responsibility) (advice) (menu of options) (empathy) (self-efficacy)	59	"Established rapport and introduces the session" "Asks questions as written"	Yes, No, Don't Know, Not Applicable
Secondary Traumatic Stress Scale (STSS)	2004	Self-Report	(Intrusion) (Avoidance) (Arousal)	17	"My heart started pounding when I thought about my work with patients." "It seemed as if I was reliving the trauma(s) experienced by my patient(s)."	five-point Likert scale ranging from “Never” to “Very Often”
Security of “I” Assessment (SofIA)	2016	Peer/Corollary Report	(emotions) (personality) (rumination) (worry)	13		
Self and Interpersonal Functioning Scale (SIFS)	1980	Self-Report	(identity) (self-direction) (empathy) (intimacy)	24	"I often have tender, concerned feeling for people less fortunate than me" "When I see someone being taken advantage of, I feel kind of protective toward them"	7 point LIkert scale
Self Dyadic Perspective-Taking Scale (SDPT)	1990	Self-Report	(strategies) (cognizance)	23	"In my relationship with my partner I believe that there are two sides to every question, and I try to look and think about both sides." "When involved in an argument with me, my partner is the type of person who will consider and take into account my point of view and compare that with his/her own."	5-point Likert-type scale ranging from 0 (totally disagree) to 4 (totally agree)
Self versus Other Interest Implicit Association Test (SOI-IAT)	2020	Self-Report	(altruistic personality scale) (prosocial personality battery) (interpersonal reactivity index) (hypothetical dictator game)	40	“I try to look at everybody’s side of a disagreement before I make a decision” “I am quite touched by things that I see happen”	5 point and 7 point Likert-type scales
Self-Care Counseling Rubric (SCCR)	2013	Peer/Corollary Report	(attitude and attentiveness) (current complaint assessment) (treatment suggestions) (medication overview) (closure and teach-back)	14	"No empathy or made insincere statements of empathy, encouragement, and concern. Did not state the intention to help. No relationship with the patient." "Little or no eye contact. Constant nervous movement. Did not maintain an appropriate distance from the patient."	Four-point, Likert-type scale: 1 = unsatisfactory, 2 = needs development, 3 = satisfactory, and4 = commendable.
Self-Compassion and Self-Criticism Scales (SCCS)	2015	Self-Report	(self-compassion) (self-criticism)	5	"I try to see my failing as part of the human condition" "When i am going through a very hard time, I give myself the caring and tenderness I need"	5-point Likert Scale of frequency (1 = almost never; 5 = almost always)
Self-Compassion Scale - Short Form (SCS-SF)	2003	Self-Report	(Self-kindness) (Self-judgement) (Common humanity) (Isolation) (Mindfulness) (Over-identification)	12	"How empathetic is this child?" "Shows concern for others."	5-point Likert-scale response format (1 = 'Almost Never' to 5 = 'Almost Always')
Self-Compassion Scale - Youth Version (SCS-Y)	2003	Self-Report	(self-kindness) (mindfulness) (common humanity) (self-judgment) (isolation) (over-identification)	36	"When I fail at something important to me, I become consumed by feelings of inadequacy." "I try to see my failing as part of the human condition."	5-point Likert-type scale ranging from 1 (almost never) to 5 (almost always)
Self-Compassion Scale (SCS)	2003	Self-Report	(self-kindness) (self-judgment) (common humanity) (isolation) (mindfulness) (over-identification)	N/a	"When I fail at something important to me, I become consumed by feelings of inadequacy." "I try to see my failing as part of the human condition."	5-point Likert-type scale ranging from 1 (almost never) to 5 (almost always)
Self-Connection Scale (SCS)	2021	Self-Report	(awareness) (acceptance) (alignment)	12	“I have a deep understanding of myself” “It is easy for me to identify and understand how I am feeling in any given moment”	seven-point scale (1= strongly disagree; 7=strongly agree)

Instrument	Published Year	Type of Measure	Subscales/ Forms	Total Number of Items	Sample Item(s)	Response set
Self-Efficacy for Mindfulness Meditation Practice Scale	1989	Self-Report	(attention) (compassion) (emotion)	9	"I am able to maintain compassion toward myself" "I am able to be compassionate with myself when my mind wanders"	9-point response scale that went from never (1), rarely (3), sometimes (5), usually (7), always (9)
Self-focused Practice Questionnaire (SfPQ)	2015	Self-Report	(the therapist-self) (the personal-self)	30	"Right now, my understanding of the reasons for patients' emotions and behaviors in therapy is better than it was" "SPSR/SFP/PT made me a better therapist when working with clients"	4-point Likert scale: 0 (do not agree), 1 (slightly agree), 2 (definitely agree) and 3 (strongly agree)
Self-Other Four Immeasurables Scale (SOFI)	2009	Self-Report	(positive qualities toward self) (positive qualities toward others) (negative qualities toward self) (negative qualities toward others)	8	"Indicate to what extent you have thought, felt, or acted this way toward yourself and others during the past WEEK: Friendly—toward myself" Friendly—toward others"	5-point Likert-type scale ranging from 1 (very slightly or not at all) to 5 (extremely)
Semi-Structured Interview for Personality Functioning DSM–5 (STiP-5.1)	2013	Self-Report	No Subscales	28		
SERVPERF-R	1988	Peer/Corollary Report	(tangibles) (reliability) (responsiveness) (assurance) (empathy)	15	"They have my best interests at heart" "Receiving personal attention"	7-point Likert scale(1 =strongly disagree, 7 = strongly agree)
SERVQUAL (SERVice QUALity)	1988	Peer/Corollary Report	(empathy) (relationship of mutual respect) (dignity) (understanding of illness) (religious needs) (food and physical environment)	15	"They have my best interests at heart" "Receiving personal attention"	7-point Likert scale(1 =strongly disagree, 7 = strongly agree)
Severity Indices of Personality Problems-Short Form (SIPP-SF)	2008	Self-Report	(Self-Control) (Identity Integration) (Social Concordance) (Responsibility) (Relational Capacities)	60		
Sex Trafficking Attitudes Scale (STAS)	2016	Self-Report	(attitudes toward ability to leave sex trafficking) (efficacy to reduce sex trafficking) (knowledge about sex trafficking) (empathic reactions toward sex trafficking) (attitudes toward helping survivors) (awareness of sex trafficking)	27	"I become emotional thinking about trafficking" "I can make a difference for trafficked persons"	Likert-type scale ranging from 1 (strongly disagree) to 6 (strongly agree)
Sexual Narcissism Scale	2010	Self-Report	(sexual exploitation) (sexual entitlement) (low sexual empathy,and sexual skill)	20	"The feelings of my sexual partners don't usually concern me" "When I sleep with someone, I rarely know what they are thinking or feeling"	5-point scale (1 = Strongly Disagree to 5 = Strongly Agree)
Shared Care Instrument (SCI)	2005	Self-Report	(communication) (decision making) (reciprocity) (balance)	26	"When my family member is not feeling well, s/he decides when to call the doctor." "I sacrifice my own needs."	a 6-point Likert-type scale ranging from strongly disagree (1) to strongly agree (6)
Shared Care Instrument-Revised (SCI-R)	2005	Self-Report	(communication) (decision making) (reciprocity)	19	"When my family member is not feeling well, s/he decides when to call the doctor." "I sacrifice my own needs."	a 6-point Likert-type scale ranging fromstrongly disagree (1) to strongly agree (6)
Short-Form Five Facet Mindfulness Questionnaire (FFMQ-SF)	2013	Self-Report	(describing experience with language) (observe) (acting with awareness) (nonjudgment of experience) (nonreactivity to inner experience)	20	"I criticize myself for having irrational or inappropiate emotions." "When I have distressing thoughts or images, I feel calm soon after."	5-point Likert-type scale ranging from 1 (never or very rarely true) to 5 (very often or always true)
Sinclair Compassion Questionnaire (SCQ)	2021	Peer/Corollary Report	No Subscales	15	Condensed: "Feel cared for" "Genuine concern"	5-point Likert scale of agreement (1=strongly disagree, 2=disagree, 3=neutral, 4=agree, 5=strongly agree)
Single Item Trait Empathy Scale (SITES)	2018	Self-Report	No Subscales	1	"When I see someone being taken advantage of, I feel kind of protective towards them" "When I see someone who badly needs help in an emergency, I go to pieces"	5-point Likert scale ranging from "Does not describe me well" to "Describes me very well".
Single-Item Measure of Depersonalization	2009	Self-Report	No Subscales	1	"I have become more callous toward people since I took this job"	7-point Likert scale with response options ranging from "Never" to "Daily"
Single-Item Measure of Emotional Exhaustion	2009	Self-Report	No Subscales	1	"I feel burned out from my work"	7-point Likert scale with response options ranging from "Never" to "Daily"
Single-Item Mindfulness Scale (SIMS)	2022	Self-Report	No Subscales	1	To what extent do you agree with this statement: "I am a mindful person." (Note: a mindfulperson pays attention to the present, is less concerned about the past or future, and accepts things as they are).	1= not at all, 4 = moderately agree, 7 = strongly agree
Single-Item Self-Compassion Scale (SISC)	2022	Self-Report	No Subscales	1	I have high self-compassion	
Situational Judgment Tests (SJTs)	2014	Self-Report	No Subscales	40		
Social Anhedonia Scale (SAS)	1976	Self-Report	No Subscales	40	"I have warm relationships with some people" "I feel family decisions need to be made jointly"	3-point scale ranging from strongly like you (1) to essentially unlike you (3)

Instrument	Published Year	Type of Measure	Subscales/ Forms	Total Number of Items	Sample Item(s)	Response set
Social Cognition and Functioning (SCAF)	2013	Behavioural/Expert Coder	(basic biological motion) (emotion in biological motion) (self-referential memory) (empathetic accuracy) (facial affect identification)	N/a		
Social Competence Interview	1991	Peer/Corollary Report	(interpersonal skills) (goal-oriented strivings in coping social impact)	36		
Social Emotional Health Survey Higher Education (SEHS-HE)	2017	Self-Report	(self-efficacy) (persistence) (self-awareness) (belief-in-others) (family support) (institutional support) (peer support) (emotional competence) (cognitive reappraisal) (empathy) (self-regulation) (engaged living) (gratitude) (zest) (optimism)	36	"I am aware of others' hardships"<br>"I feel badly when my friends are put down"	6 point Likert-type scale from 1 (very much unlike me) to 6 (very much like me)
Social Issues Advocacy Scale (SIAS)	2013	Self-Report	(political and social advocacy) (confronting discrimination) (political awareness) (social issue awareness)	21	"I participate in demonstrations or rallies about social issues that are important to me"<br>"I volunteer for political causes and candidates I believe in"	Likert-type scale (1 = strongly disagree that it describes me; 6 = strongly agree that itdescribes me)
Social Justice and Empathy Assessment (SJEA)	2021	Self-Report	(social justice) (Empathy) (CAPE Educational Outcomes)	17	"I plan to stay abreast of current laws and policies affecting populations with which I work"<br>"I am aware that there are effects of oppression on clients/patients"	six-point Likert scale ranging from strongly agree to strongly disagree and high competence to low competence
Social Relations Survey (SRS)	1990	Self-Report	(social assertiveness) (directiveness) (defense of rights) (confidence) (empathy)	128	"When a friend expresses anxlety over some problem, I too become troubled"<br>"I usually maintain a cool and detached feeling toward people"	6 point Likert-type scale from very much (I), moderately (2), slightly (3), and slightly (4), moderately (5), and very much (6)
Social Self-Compassion Scale (SSCS)	2020	Self-Report	(self-criticism) (self-compassion)	12	"When I'm having a hard time in social situations, I give myself the caring and tenderness I need"<br>"I'm intolerant and impatient towards myself when socially anxious"	
Social Skills Imporvement System Rating Scales Parent Form (SSiS)	2008	Self-Report	(empathy and prosocial skills) (engagement and relationship skills) (cooperation) (self-control)	46	NA	4-point scale ("never," "seldom," "often," "almost always")
Social Skills Improvement System (SSIS)	2008	Peer/Corollary Report	(self-regulation/responsibility) (social competence) (empathy)	39	"Accepts responsibility when he/she needs to."<br>"Makes friends easily."	Four-point Likert-type scale
Social-Emotional Assets and Resilience Scale - Parent Form (SEARS-P)	2008	Peer/Corollary Report	(self-regulation/responsibility) (social competence) (empathy)	39	"Accepts responsibility when he/she needs to."<br>"Makes friends easily."	Four-point Likert-type scale
Social-Emotional Responding Task (SERT)	2017	Self-Report	(aggressive acts) (prosocial omission) (social exclusion)	6	Imagine that you are at school. Anotherboy in your class, Mark, shows you a chocolate bar he brought fromhome. He then puts it back in his backpack. You really likechocolate. When Mark leaves, you wait until nobody is watching,and you take the chocolate bar from his backpack so you can eat it."Anticipated emotions were elicited by the question"How would youfeel if you had done this?."	
Solidarity Scale	2016	Peer/Corollary Report	(altruism) (solidarity)	22	"If violence against people of my race is done by police or other people in New York City, I should speak up"<br>"If violence against people of my race is done by police or other people in New York City, I should offer support to the victim or his/her family"	1: disagree strongly; 2: disagree somewhat; 3: neither disagree nor agree; 4: agree somewhat; 5: agree strongly (6: not applicable; 7: don't know; 8: refused to answer/missing)
Spatial Perspective Task (SPT)	2008	Self-Report	No Subscales	N/a	Reading the mind inthe eyes task (RMET) - not questionaire-based study	
Spiritual Modeling Inventory of Life Environments (SMILE)	2009	Self-Report	No Subscales	18		
Sports Injury Clinic Athlete Satisfaction Scale (SICASS)	1995	Self-Report	(evaluation of empathy) (information given) (competence)	16	"I felt free to talk to my physiologist about the things that were bothering me"<br>"The physiologists was very sensitive towards me needs as an active person"	5-point Likert-type scale ranging from 1 (strongly disagree) to 5 (strongly agree)
Staff Attitudes Towards Patients who Attempt Suicide [Unnamed]	1998	Self-Report	(emotional attitudes) (empathic or rejecting) (attitudes toward further training) (attitudes toward the need for psychiatric care)	100	"A patient who has tried to commit suicide usually takes up a great deal of staff time"<br>"Once people have made up their minds to commit suicide, you cannot stop them"	
Staff-Patient Interaction Response Scale for Palliative Care Nursing (SPIRS-PCN)	1990	Behavioural/Expert Coder	No Subscales	10	"Frank is a patient in his mid-60s. He was admitted to hospital 4 days ago for chemotherapy for advanced prostate cancer."<br>"Anne is a patient in her mid-20s with ahistory of intravenous drug use who was admitted tohospital 2 days ago for a liver biopsy. She is positive for Hepatitis B and HIV."	Short written response
Standardized Assessment of Severity of Personality Disorder (SASPD)	2018	Self-Report	No Subscales	9		

Instrument	Published Year	Type of Measure	Subscales/ Forms	Total Number of Items	Sample Item(s)	Response set
State Empathy Scale [Unnamed]	2010	Self-Report	(affective empathy) (cognitive empathy) (associative empathy)	12	"I experienced the same emotions as the character when watching this message" "I can feel the character's emotions"	5-point Likert scale anchored at both poles (0 = “not at all,” and 4 = “completely”)
State Moral Elevation Scale (SMES)	2022	Self-Report	No Subscales	9	To what extent did you expereince each of the followingn statements while [insert time/context specifier]: "Motivated to live in a nobler or virtuous way" "Somehow 'lifted up' or in touch with the better parts of myself"	Likert-type scale was used with ratings from 0 (not at all) to 4 (extremely)
State Self-Compassion Scale-Long (SSCS-L)	2020	Self-Report	(Self-Kindness) (Common Humanity) (Mindfulness) (Self-Judgment) (Isolation) (Overidentification)	18	"I'm giving myself the caring and tenderness I need" "I see my difficulties as part of life that everyone goes through"	1 (not at all true for me) to 5 (very true for me)
State Self-Compassion Scale-Short Form (SSCS-S)	2020	Self-Report	(Self-Kindness) (Common Humanity) (Mindfulness) (Self-Judgment) (Isolation) (Overidentification)	6	"I'm giving myself the caring and tenderness I need" "I'm remembering that there are lots of others in the world feeling like I am"	1 (not at all true for me) to 5 (very true for me)
State-Empathic Concern Scale	2019	Self-Report	(perspective taking) (empathic concern) (fantasy) (personal distress)	5	"I tend to lose control during emergencies" "When I am reading an interesting story or novel, I imagine how I would feel if the events in the story were happening to me"	5-point Likert-type scale ranging from 0 (does not describe me well) to 4 (describes me very well)
State-Interpersonal Reactivity Index (S-IRI)	1980	Self-Report	(State empathic concern) (State perspective taking) (State personal distress)	14	"I had tender, concerned feelings for my client when he/she discussed his/her concerns." "It was difficult to see things from my client's point of view."	
State-Trait Scales for Distinct Positive Emotions (STS-DPE)	2020	Self-Report	(Admiration) (Awe) (Gratitude) (Sympathy) (Tenderness) (Empathy) (Interest) (Hope) (Enthusiasm) (Schadenfreude) (Amusement) (Contentment) (Nurturant Love) (Romantic Love) (Attachment Love)	101	To what extent does each of the following statements characterize your experience of [TARGET EMOTION] "I felt admiration" "I felt a desire to become more like a specific person"	Anchors: 1 = not at all, 3 = somewhat, 5 = very much
Suicidal Patients Scale (USP Scale)	1998	Self-Report	(emotional attitudes) (empathic or rejecting) (attitudes toward further training) (attitudes toward the need for psychiatric care)	100	"A patient who has tried to commit suicide usually takes up a great deal of staff time" "Once people have made up their minds to commit suicide, you cannot stop them"	
Suicide Attitude Vignette Experience (SAVE) Scale	1984	Self-Report	(failure to achieve academically) (break-up of a relationship) (parental disapproval) (rejection because of physical appearance) (mental loss caused by use of drugs) (physical loss of the use of legs) (terminal illness associated with pain) (parental discord and divorce) (death of a parent) (guilt over causing an accident)	10	"Carol has had leukemia since she was 13. She is now 17. She has known for over a year that the disease is most likely going to kill her. Lately the pain has increased to the point where the drugs no longer control it. Carol attempts suicide" "Tom is an 18-year-old who was in an automobile accident last year. He had been a member of the football team and was very active in school activities. Now he is paralyzed from the waist down. He attempts suicide"	5-point Likert-type scale to how much they sympathize, empathize, and agree with action
Sulliman Scale of Social Interest (SSSI)	1994	Self-Report	(social interest) (empathy) (interpersonal contact) (happiness) (narcissism) (fantasy and personal distress empathy)	50	"He/she listens well in a conversation." "He/she is cooperative with others."	True/False
Survey of Physician Humanistic Approaches to Patient Care	1993	Peer/Corollary Report	No Subscales	5		
Sussex-Oxford Compassion for Others Scale (SOCS-O)	2020	Self-Report	(recognizing suffering) (understanding the universality of suffering) (feeling for the person suffering) (tolerating uncomfortable feelings) (motivation to act/acting to alleviate)	20	"I notice when others are feeling distressed" "I understand that everyone experiences suffering at some point in their lives."	5-point Likert-type scale ranging from 1 (not at all true) to 5 (always true)
Sussex-Oxford Compassion for the Self Scale (SOCS-S)	2020	Self-Report	(recognizing suffering) (understanding the universality of suffering) (feeling for the person suffering) (tolerating uncomfortable feelings) (motivation to act/acting to alleviate)	20	"I notice when others are feeling distressed" "I understand that everyone experiences suffering at some point in their lives."	5-point Likert-type scale ranging from 1 (not at all true) to 5 (always true)
Systemizing Quotient - Child (SQ-C)	2009	Self-Report	No Subscales	28	“I find it hard to understand how to behave in a social situation” "In a conversation, I tend to focus on my own thoughts rather than on what my listener might be thinking."	5-point Likert-type scale ranging from 0 (strongly disagree) to 4 (strongly agree)
Tape Excerpt Response Procedure (TERPs)	1972	Behavioural/Expert Coder	No Subscales	64		
Taxonomy of Compassionate Physician Behaviours [Unnamed]	2011	Behavioural/Expert Coder	(recognition of the patient's suffering) (emotional resonance and movement towards addressing suffering)	N/a		
Test of Accurate Perception of Patients’ Affect (TAPPA)	2014	Self-Report	No Subscales	48	What was the patient thinking or feeling the moment the tape stopped?	different answer choices specific to each question

Instrument	Published Year	Type of Measure	Subscales/ Forms	Total Number of Items	Sample Item(s)	Response set
Test of Self-Conscious Affect - Adolescent (TOSCA-A)	1991	Self-Report	(guilt) (shame)	64	"It makes me sad to see a girl who can't find anyone to play with" "Seeing a boy who is crying makes me feel like crying"	4-point scale ranging from 1 = Strongly Disagree to 4 = Strongly Agree
Therapeutic Environment Scales (TESS)	2014	Self-Report	(respondent's experience with staff) (experience with non-staff members) (other residents or patients) (processes that do not relate to interactions within the community)	9		
Therapist Empathy Scale (TES)	2015	Behavioural/Expert Coder	No Subscales	9	A therapist conveys concern by showing a regard for and interest in the client. The therapist seems engaged and involved with the client and attentive to what the client has said. The therapist's voice has a soft resonance that supports and enhances the client's concerned expressions.	7-point Likert-type scale (1 = not at all, to 7 = extensively)
Therapists' Systemic Response Rating Scale [Unnamed]	2000	Behavioural/Expert Coder	(interactional sequences) (consideration of context)	N/a		
Thought Impact Scale (TIS)	2020	Self-Report	(physical sensations/reactions in response to thoughts or emotions) (spontaneous vivid imagery in response to specific thoughts or situations) (spontaneous influence on behavior by imagination/nonconscious thought content) (spontaneous superimposing of a sense of meaning on neutral or ambiguous external stimuli) (spontaneous emotional responses to imagery) (spontaneous useful memory modulation) (effortless completion of complex familiar tasks with no conscious attention) (narrowed awareness of or dissociation from here-and-now in response to compelling thoughts) (personally valuable knowledge emerging spontaneously in consciousness)	39	"Other people's misfortunes do not disturb me a great deal" "When someone else is feeling excited, I tend to get excited too"	5 point response scale of never, rarely, sometimes, often, and always
Tool to Assess Inpatient Satisfaction with Care from Hospitalists (TAISCH)	2012	Self-Report	No Subscales	17	"Compared to all other physicians that you know, how do you rate Dr. X's compassion, empathy, and concern for you" "Dr. X showed interest in my views and opinions about my health"	5-point Likert scales
Toronto Empathy Questionnaire (TEQ)	2009	Self-Report	No Subscales	16	"Other people's misfortunes do not disturb me a great deal" "When someone else is feeling excited, I tend to get excited too"	5-point Likert scale (i.e., never, rarely, sometimes, often, always)
Trait Emotional Intelligence Questionnaire Short Form (TEIQue-SF)	2003	Self-Report	(self-confidence) (empathy) (uncertainty) (social connection)	30	"I'm normally able to "get into someone's shoes" and experience their emotions" "I don't seem to have any power at all over other people's feelings"	7-point Likert scale ranging from 1 (completey disagree) to 7 (completely agree)
Trait Sympathy Scales (TSS)	2008	Self-Report	(sympathy for the disempowered) (sympathy for the feelings of others) (sympathy for others)	18	"It breaks my heart to hear about people with disabilities getting made fun of for their disabilities" "To see an elderly person fall down and get hurt would really break my heart"	7-point scale ranging from 1 (strongly disagree) to 7 (strongly agree)
Transactional Analysis Ego States	1980	Self-Report	(thrill and adventureseeking) (experience seeking) (disinhibition) (boredom susceptibilit)	300		
Transpersonal Gratitude Scale	2014	Self-Report	(expression of gratitude) (value of gratitude) (transcendent gratitude) (spiritual connection)	16	"Thinking of the blessings that I've received helps me to appreciate life" "I show appreciation to others when they have positively influenced my life."	6-point Likert scale ranging from 1 (strongly disagree) to 6 (strongly disagree)
Trauma-Informed Self-Care Measure	2008	Self-Report	(utilizing organisational resources) (organizational practices) (professional self-care)	10	"I would suggest mental health counseling for a coworker needing additional support" "I balance my caseloads so that I am not dealing only with traumatized children and their families"	Likert response of not at all = 0; to a slight extent = 1; to a moderate extent = 2; to a great extent = 3; and to a very great extent = 4
Trauma-Related Shame Inventory - Short Form (TRSI-SF)	2022	Self-Report	(internal shame) (external shame)	10	"I am ashamed of myself because of what happened to me." "If others knew what happened to me, they would be disgusted with me"	4-point scale (0 to 3) where higher scores indicate elevated levels of trauma-related shame

Instrument	Published Year	Type of Measure	Subscales/ Forms	Total Number of Items	Sample Item(s)	Response set
Truax Accurate Empathy Scale (AE)	1961	Behavioural/Expert Coder	(activity of therapist) (uncertain attention to emotion) (attention to relationship betweenclient's actions andemotion) (uncertain attention to meaning) (verbosity) (use of unusual verbal style) (attention to client'sactions)	23		
Twelve Step Facilitation Adherence Competence Empathy Scale (TSF ACES)	2012	Self-Report	No Subscales	8		
Unconditional Self-Kindness Scale	2018	Self-Report	No Subscales	6	“How much are you loving and kind to yourself when you are criticized or rejected by another person” “How much are you patient and tolerant with yourself when you fail and make a mistake”	6-point Likert-type scale ranging from 0 (not at all) to 6 (a great deal)
Undergraduate Nursing Palliative Care Knowledge Survey (UNPCKS)	2020	Self-Report	(Principles of Primary Palliative care) (Pain and Provider Self-Care)	27	NA	a multiple-choice format similar to the ELNEC-KAT(Lange et al., 2009)
Unit Manager Support Scale (UMSS)	2003	Peer/Corollary Report	No Subscales	6	"My unit manager recognizes the standards of care I try to deliver" "My unit manager knows me well enough to know when I have concerns about patient care and tries to understand my point of view."	5-point Likert-type scale ranging from 1 (Never) to 5 (Always)
Universal Worth Scale (UWS)	2010	Self-Report	(personal value) (noncontingence) (universality)	12	"When I fail at something, I try to remember that everybody fails sometimes too" "When I'm going through a very hard time, I'm really nice to myself"	5-point Likert scale from 1 (strongly disagree), 2 (disagree), 3 (neutral), 4 (agree), and 5 (strongly agree)
Verona Coding Definitions of Emotional Sequences (VR-CoDES)	2009	Self-Report	No Subscales	10		
Vicarious Posttraumatic Growth Inventory (VPTGI)	2023	Self-Report	(Negative response) (Changes in world view) (Creating meaning to change self) (Changes in interpersonal relationships) (Engaging in efforts of support and self-care) (Client progress impacting growth)	32	"As a result of my work with clients or patients who have experienced trauma, I express more kindness in my relationships." "As a result of my work with clients or patients who have experienced trauma, I am more empathic toward othres and their experiences."	6-point Likert scale of agreement
Vicarious Resilience Scale (VRS)	2015	Self-Report	(increased resourcefulness) (changes in life goals) (increased self-awareness) (client inspired hope) (increased recognition of spirituality) (increased consciousness of power) (increased capacity to remain present)	27	"More compassion for people" "Better at self-care"	6-point response scale: Did not experience this (0), Experienced this to avery small degree (1), Experienced this to a small degree (2),Experienced this to a moderate degree (3), Experienced this to a great degree (4), Experienced this to avery great degree (5)
Vicarious Trauma Organizational Readiness Guide (VT-ORG)	2013	Self-Report	(leadership and mission) (management and supervision) (employee empowerment and work environment) (training and professional development) (staff health and wellness)	63	"Leadership demonstrates a clear and decisive vision that supports and articulates the mission of the organization" "Leadership models, values, and promotes open and respectful communication among staff"	Likert-type scale (1 = never, 2 = rarely, 3 = sometimes, 4 = often, 5 = always)
Vicarious Trauma Scale (VTS)	2008	Self-Report	No Subscales	8	"I find myself distressed by listening to my clients' stories and situations" "Sometimes it is hard to stay positive and optimistic given some of the things I encounter in my work"	5-point Likert scale from 1 (strongly disagree), 2 (disagree), 3 (neutral), 4 (agree), and 5 (strongly agree)
Victim Concern Scale (VCS)	2006	Self-Report	(general concern for victims) (concern for victims of violent crimes) (concern for victims of property crimes/theft) (concern for culpable victims)	100		
Victim Empathy Response Assessment (VERA)	2008	Self-Report	(non-offending focus) (sexual offense focus)	5	"Worried" "Sad"	five-point Likert scale ranging from 0 (not at all) to 4 (very much so)
Visual CARE Measure	2002	Self-Report	No Subscales	10		
Visual CARE Measure 10Q	2012	Peer/Corollary Report	No Subscales	10	How was the therapist at... "...making you feel happy and relaxed? (being friendly and caring and making you feel calm)" "...asking questions and letting you talk? (being interested in you and giving you time to speak)"	a visual 5-point scale and symbols to support understanding of each questions
Visual CARE Measure 10Q Parent	2012	Peer/Corollary Report	No Subscales	10		a visual 5-point scale and symbols to support understanding of each questions

Instrument	Published Year	Type of Measure	Subscales/ Forms	Total Number of Items	Sample Item(s)	Response set
Visual CARE Measure 5Q	2012	Peer/Corollary Report	No Subscales	5	How was the therapist at... "...making you feel happy and relaxed? (being friendly and caring and making you feel calm)" "...asking questions and letting you talk? (being interested in you and giving you time to speak)"	a visual 5-point scale and symbols to support understanding of each questions
Warmth/Affection Coding System (WACS)	2023	Behavioural/Expert Coder	(Tone/Pitch of Voice) (Vocally Expressed Affection) (Facial Expression) (Physical Touch) (Reciprocal Play)	N/A		
Working Alliance Inventory - General Pracitce (WAI-GP)	2021	Self-Report	(Goals) (Tasks) (Bond)	12	"I believe my GP cares about me." "I feel that my GP understands me."	5-point Likert scale, ranging from 'strongly agree' (1) to 'strongly disagree' (5)
Workplace Compassion Scale	2023	Self-Report	(noticing) (empathizing) (sensemaking) (acting)	12	"I experience pain from people's distress in my workplace." "When I see someone in distress in my workplace, I try to act as quickly as possible."	7-point response scale from almost never true to almost always true
Young Children's Empathy Measure (YCEM)	1990	Self-Report	(cognitive perspective taking) (affective perspective taking)	6	"A child had just lost its best friend" "A child really wants to go out but is not allowed"	response to the question "How do you feel about this?" from 4 = exact match to the intended emotion; 3 = sirmlar emotion; 2 = some emotion; 1 = nonemotional response; and, 0 = no response
Youth Suicide Scale (YSS)	1980	Self-Report	No Subscales	11	"Belief about the psychological disturbance of the child" "How difficult to express sympathy toward the parent"	7 point Likert scale

S3 Table. Measures of compassion and empathy. S3 Table includes all of the compassion and empathy measures that were identified with subscales and sample items, sorted alphabetically.
